# Supplementary material for: Are there researcher allegiance effects in diagnostic validation studies of the PHQ-9? A systematic review and meta-analysis
Source: BMJ Open. 2017 Sep 29;7(9):e015247. doi: 10.1136/bmjopen-2016-015247 (PMC5640143; doi:10.1136/bmjopen-2016-015247)

Appendices to: Manea L, Boehnke JR, Gilbody S, Moriarty AS, McMillan D, Are there researcher allegiance effects in diagnostic validation studies of the PHQ-9? A systematic review and meta-analysis. Manuscript submitted for publication at BMJOpen.

Appendix 1: Search terms used in Embase, MEDLINE and PsycINFO

(phq adj5 “9”).ti,ab.

(phq adj5 item$).ti,ab.

(patient health questionnaire adj5 “9”).ti,ab.

(patient health questionnaire adj5 item$).ti,ab.

(prime md adj5 “9”).ti,ab.

(prime md adj5 item$).ti,ab.

Appendix 2

Figure 1: PRISMA flowchart - search and selection of included diagnostic accuracy studies for the systematic review of studies reporting diagnostic accuracy of the PHQ-9 at using the summed items scoring method (Manea et al, 2014)

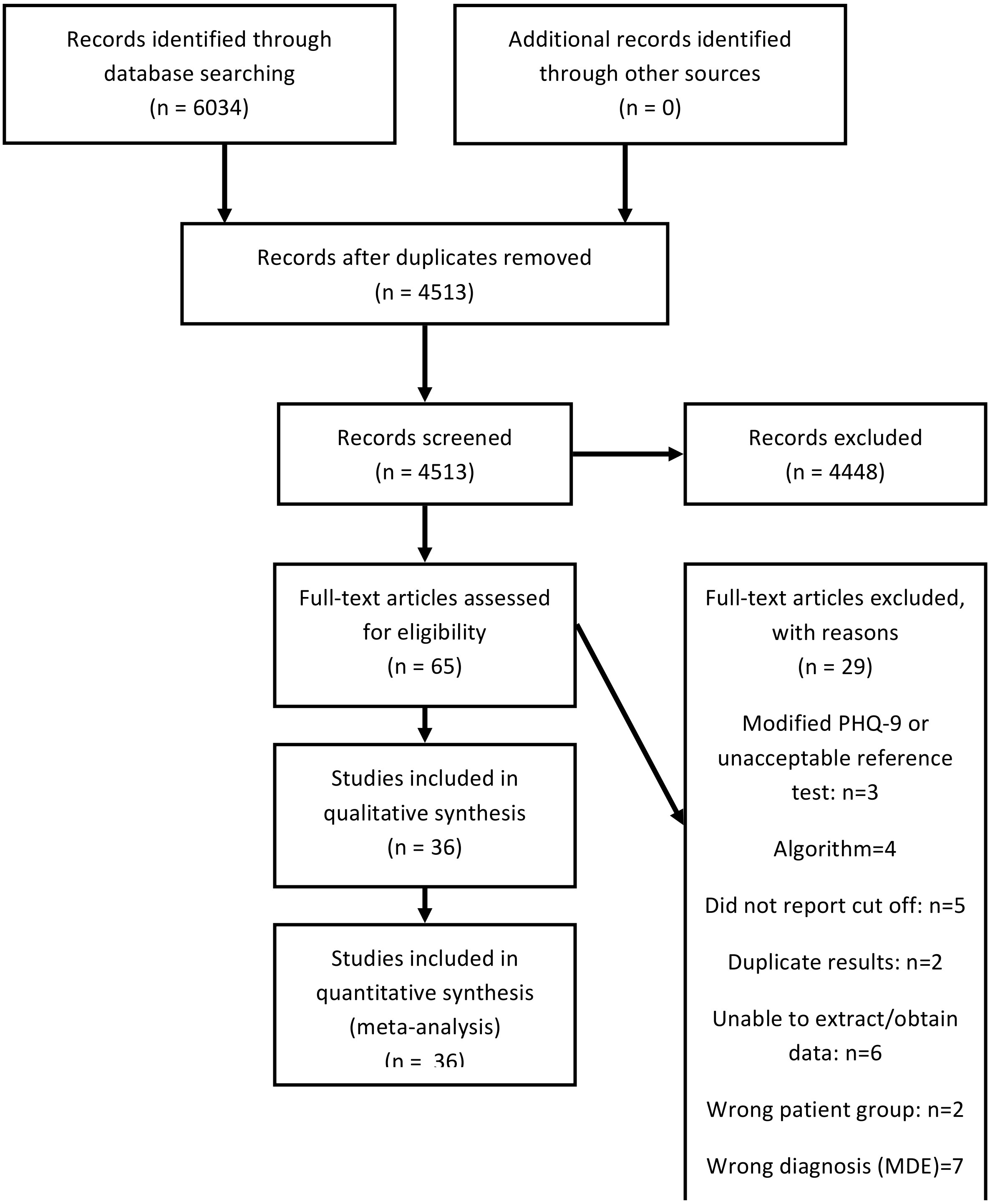

Figure 2: PRISMA flowchart - search and selection of included diagnostic accuracy studies for the systematic review of studies reporting diagnostic accuracy of the PHQ-9 at using the algorithm scoring method (Moriarty et al., 2015)

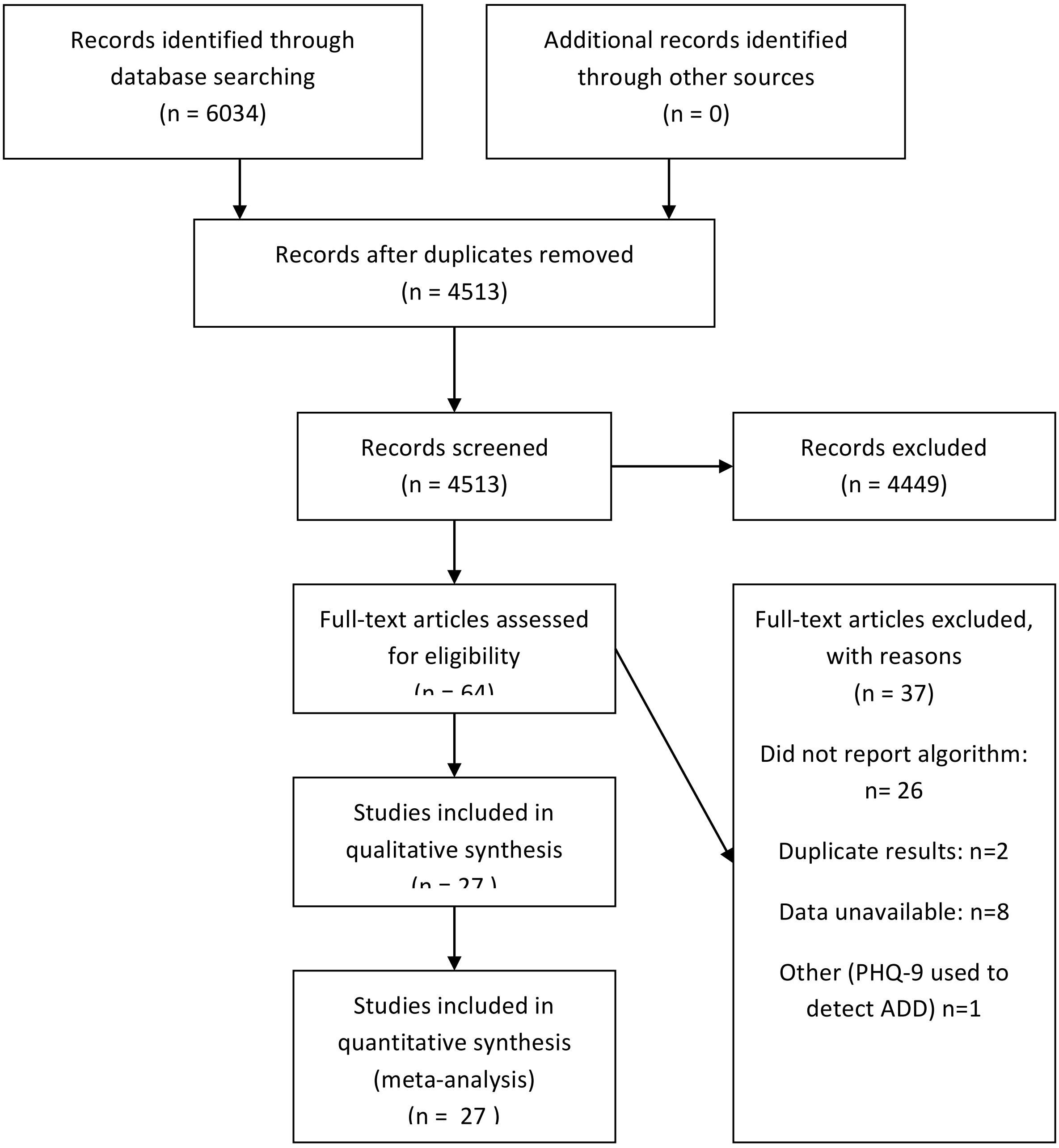

Supplement: Supplementary file 1 [file bmjopen-2016-015247supp001.pdf]
