# Supplementary material for: Are there researcher allegiance effects in diagnostic validation studies of the PHQ-9? A systematic review and meta-analysis
Source: BMJ Open. 2017 Sep 29;7(9):e015247. doi: 10.1136/bmjopen-2016-015247 (PMC5640143; doi:10.1136/bmjopen-2016-015247)
Supplement: Author's manuscript [file bmjopen-2016-015247.draft_revisions.pdf]

BMJ Open is committed to open peer review. As part of this commitment we make the peer review history of every article we publish publicly available.

When an article is published we post the peer reviewers' comments and the authors' responses online. We also post the versions of the paper that were used during peer review. These are the versions that the peer review comments apply to.

The versions of the paper that follow are the versions that were submitted during the peer review process. They are not the versions of record or the final published versions. They should not be cited or distributed as the published version of this manuscript.

BMJ Open is an open access journal and the full, final, typeset and author-corrected version of record of the manuscript is available on our site with no access controls, subscription charges or pay-per-view fees (http://bmjopen.bmj.com).

If you have any questions on BMJ Open's open peer review process please email info.bmjopen@bmj.com

BMJ Open

Are there researcher allegiance effects in diagnostic validation studies of the PHQ-9? A systematic review and meta-analysis

Journal:	*BMJ Open*
Manuscript ID	bmjopen-2016-015247
Article Type:	Research
Date Submitted by the Author:	23-Nov-2016
Complete List of Authors:	Manea, Laura; University of York, Health Sciences<br>Boehnke, Jan; University of York<br>Gilbody, Simon; The University of York, Department of Health Sciences<br>Moriarty, Andrew; University of York, Health Sciences<br>McMillan, Dean; University of York, Department of Health Sciences
**Primary Subject Heading**:	Mental health
Secondary Subject Heading:	Diagnostics
Keywords:	Depression & mood disorders < PSYCHIATRY, Screening, PHQ-9, diagnostic meta-analysis, allegiance effect

SCHOLARONE™ Manuscripts

For peer review only

Are there researcher allegiance effects in diagnostic validation studies of the PHQ-9? A systematic review and meta-analysis

Laura Manea MMedSci MRCPsych*, Jan Boehnke PhD, Simon Gilbody DPhil FRCPsych FRSA, Andrew Moriarty MSc, Dean McMillan PhD

*Corresponding Author

Hull York Medical School and Department of Health Sciences, ARRC Building, University of York, YO10 5DD

Email: laura.manea@york.ac.uk

For peer review only

Abstract

Objectives To investigate whether an authorship effect is found that leads to better performance in studies conducted by the original developers of the PHQ-9 (non-independent studies).

Design Systematic review with random effects bivariate diagnostic meta-analysis. Search strategies included electronic databases, examination of reference lists, and forward citation searches.

Inclusion criteria Included studies provided sufficient data to calculate the diagnostic accuracy of the PHQ-9 against a gold standard diagnosis of major depression using the algorithm or the summed item scoring method at cut-off point 10.

Data extraction Descriptive information, methodological quality criteria, and 2×2 contingency tables.

Results

Seven non-independent and twenty independent studies reported the diagnostic performance of the PHQ-9 using the algorithm scoring method. Pooled diagnostic odds ratio (DOR) for the first group was 64.40, and 15.05 for independent studies group. The allegiance status was a significant predictor of DOR variation ($p < 0.0001$).

Five non-independent studies and twenty-six independent studies reported the performance of the PHQ-9 at recommended cut-off point of 10. Pooled DOR for the non-independent group was 49.31, and 24.96 for the independent studies. The allegiance status was a significant predictor of DOR variation ($P = 0.015$).

Some potential alternative explanations for the observed authorship effect including differences in study characteristics and quality were found, though it is not clear how some of them account for the observed differences

Conclusions

Non-independent studies reported better performance of the PHQ-9. Allegiance status was predictive of variation in the DOR. Based on the observed differences between independent

For peer review only

and non-independent studies we were unable to conclude or exclude that allegiance effects are present in studies examining the diagnostic performance of the PHQ-9. This study highlights the need for future meta-analyses of diagnostic validation studies of psychological measures to evaluate the impact of researcher allegiance in the primary studies.

Strengths and limitations of this study

- An original study–the first meta-analysis of diagnostic validation studies of psychological measures to evaluate the impact of researcher allegiance.
- Using rigorous methodology–strict inclusion/exclusion and quality assessment criteria.
- We found that the allegiance effect was a significant predictor of the variation of the diagnostic odds ratio in the meta-regression analysis.
- Substantial variability observed in methodological quality of included studies.
- Based on the observed methodological differences between the independent and non-independent studies we were unable to conclude or exclude that allegiance effects are present in studies examining the diagnostic performance of the PHQ-9.

For peer review only

Research on allegiance effects has a long tradition in psychotherapy research. In this context *allegiance* describes the phenomenon that researchers and clinicians who developed a treatment approach or are for other reasons invested in it tend to find larger effect sizes in favour of their treatment than for comparison groups. (Luborsky et al., 2006) This finding has been extensively replicated (Dragioti, Dimoliatis, & Evangelou, 2015; Munder, Brütsch, Leonhart, Gerger, & Barth, 2013) and is also robust when the quality of research is controlled for. Researcher allegiance is subject of on-going debates about the design of efficacy studies as well as implications for policy. (Dragioti et al., 2015; McLeod, 2010; Winter, 2010) Researcher allegiance is also discussed widely in the literature on experimental as well as evaluation research. (Staines & Cleland, 2007) Since the motivational underpinnings of allegiance effects are potentially far more ingrained into human behaviour and decision making than previously thought (e.g., (Markman & Hirt, 2002)), they may occur commonly in clinical research in general.

Although it has been suggested that allegiance effects may play a role in the validation of psychological screening and case-finding tools (e.g., O'Shea et al., in press), systematic evaluations of this hypothesis are rare and studies that acknowledge potential allegiance effects in such studies mainly come from forensic psychology and psychiatry backgrounds. (Blair, Marcus, & Boccaccini, 2008; Lilienfeld & Jones, 2008; Singh, Grann, & Fazel, 2013; Walters, 2009) Diagnostic validation studies are geared at establishing the sensitivity and specificity of a screening or case finding tool, which is used in practice to differentiate cases from non-cases or to decide about whether further assessment or treatment is indicated or will be offered An allegiance effect in such studies would be seen in systematically higher sensitivities or specificities if the original author(s) is(are) part of the team of such a study. Such a bias would have a deleterious affect on practice through promising over-optimistic accuracy of the screening or case finding tool or in evaluating the cost-effectiveness of the measure in a screening or case-finding context.

The depression module of the Patient Health Questionnaire (PHQ-9) is a widely used depression-screening instrument in non-psychiatric settings. The PHQ-9 was developed by a team of researchers, with its development underwritten by an educational grant from Pfizer US Pharmaceuticals. (Kroenke, Spitzer, & Williams, 2001) The PHQ-9 can be scored using different methods, including an algorithm based on DSM-IV criteria and a cut-off based on summed-item scores. The psychometric properties of these two approaches have been summarised in two recently published meta-analyses. (Manea, Gilbody, & McMillan, 2015;

For peer review only

Moriarty, Gilbody, McMillan, & Manea, 2015)The goal of the current review is to investigate, based on an established database of PHQ-9 diagnostic validation studies (Manea et al., 2015; Moriarty et al., 2015), whether an allegiance effect is found that leads to an increased sensitivity and specificity in studies that were conducted by researchers closely connected to the original developers of the instrument.

METHODS

Study Selection

Similar search strategies were used in both systematic reviews. (For full details please see Manea et al. (2014) and Moriarty et al. (2015)). Embase, MEDLine and PSYCHInfo were searched from 1999 (when the PHQ-9 was first developed) to August 2013 (Manea et al., 2015) and September 2013 (Moriarty et al., 2015) respectively, using the terms "PHQ-9", "PHQ", "PHQ$" and "patient health questionnaire". The reference lists of studies fitting the inclusion criteria were manually searched and a reverse citation search in Web of Science was performed. Authors of unpublished studies were contacted and conference abstracts were reviewed in an attempt to minimise publication bias.

The following inclusion-exclusion criteria were used:

Population: Adult population. *Instrument:* Studies that used the PHQ-9. *Comparison (reference standard):* The accuracy of the PHQ-9 had to be assessed against a recognised gold-standard instrument for the diagnosis of either Diagnostic and Statistical Manual (DSM) or International Classification of Disease (ICD) criteria for major depression. Studies were included if the diagnoses were made using a standardised diagnostic structured interview schedule (e.g. Mini International Neuropsychiatric Interview (MINI), Structured Clinical Interview for DSM Disorders (SCID)). Unguided clinician diagnoses with no reference to a standard structured diagnostic schedule or comparisons of the PHQ-9 with other self-report measures were excluded. Studies were also excluded if the target diagnosis was not major depressive disorder (MDD, e.g. any depressive disorder). *Outcome:* Studies had to report sufficient information to calculate a 2*2 contingency table for the algorithm or the recommended cut-off point 10. *Study design:* Any design. *Additional criterion:* We avoided double counting of evidence by ensuring that only one study of those that reported overlapping datasets in different journals were included in the meta-analysis. Citations with

For peer review only

143 overlapping samples were examined to establish whether they contained information relevant to the research question that was not contained in the included report.

145 *Quality assessment*

146 Quality assessment was performed using the QUADAS-2 tool, a tool for evaluating the risk of bias and applicability of primary diagnostic accuracy studies when conducting diagnostic systematic reviews. (Whiting et al., 2011) It covers the areas of: patient selection, index test, reference standard and flow and timing. (Mann, Hewitt, & Gilbody, 2009) This tool was adapted for the two reviews and quality assessments were carried out by two independent reviewers for all studies included in the reviews.

152 *Data synthesis and statistical analysis*

153 We constructed 2x2 tables for cut-off point 10 (Moriarty et al., 2015) and the algorithm scoring method (Manea et al., 2015) Pooled estimates of sensitivity, specificity, positive/negative likelihood ratios, and diagnostic odds ratios were calculated using random effects bivariate meta-analysis. (Reitsma et al., 2005) Summary Receiver Operator Characteristic curves (sROC) were constructed using the bivariate model to produce a 95% confidence ellipse within ROC space. (Walter, 2002) Each data point in the summary ROC space represents a separate study, unlike a traditional ROC plot, which explores the effect of varying thresholds on sensitivity and specificity in a single study.

161 We undertook a meta-regression analysis of logit diagnostic odds ratio using research allegiance as covariate in the meta-regression model. (Lijmer, Bossuyt, & Heisterkamp, 2002; S. G. Thompson & Higgins, 2002) Analyses were conducted using STATA version 12, with the metan, metandi and metareg user-written commands.

165 *Allegiance Rating*

166 We rated authorship on a paper of any of the developers of the PHQ-9 - Kurt Kroenke, MD, Robert L Spitzer, MD, and Janet B W Williams – as an indicator of potential allegiance. We also rated as evidence of allegiance as acknowledged collaborations with the developers of the PHQ-9, even if they were not listed as co-authors or if the authors acknowledged funding from Pfizer to conduct the study.

For peer review only

RESULTS

Overview of included studies

31 studies reported the diagnostic properties of the PHQ-9 at cut-off point 10 and were included in this analysis (Moriarty et al., 2015) 27 studies were included in the algorithm review (Manea et al., 2015). The study selection flowcharts can be found in Appendix 1 (figures 1 and 2). The characteristics of these studies are reported in tables 1 and 2 and the results of the methodological assessment are presented in tables 3 and 4.

Algorithm scoring method

Descriptive characteristics

The descriptive characteristics of the included studies are presented in table 1. Seven individual studies that reported the diagnostic performance of the PHQ-9 using the algorithm scoring method were co-authored by the original developers of the PHQ-9 (Diez-Quevedo, Rangil, Sanchez-Planell, Kroenke, & Spitzer, n.d.; Gräfe, Zipfel, Herzog, & Löwe, 2004; Löwe et al., 2004; Spitzer, Kroenke, & Williams, 1999; Thekkumpurath et al., 2011), specifically acknowledged one of the developers and support by an educational grant from Pfizer US (Muramatsu et al., 2007), or were co-authored by the first author of a previous study that had also been co-authored by one of the developers (Navinés et al., 2012). Twenty independent studies reported the diagnostic properties of the PHQ-9 using the algorithm scoring method.

Three (43%, 3/7) of the non-independent studies were conducted exclusively in hospital settings (Diez-Quevedo et al., n.d.; Navinés et al., 2012; Thekkumpurath et al., 2011). The remaining four studies (67%, 4/7) were conducted in different settings or non-exclusively hospital settings: one in primary care (Spitzer et al., 1999) and three in mixed settings: psycho-somatic walk in clinics and family practices (Gräfe et al., 2004)[1], outpatient clinics

[1] This study provided separate estimates for the two settings in which it was conducted; therefore separate psychometric estimates were generated for each sample for both algorithm scoring method and summed items scoring method at cut-off point 10 (see below).

For peer review only - http://bmjopen.bmj.com/site/about/guidelines.xhtml

and family practices (Löwe et al., 2004) and primary care and hospital settings (Muramatsu et al., 2007). In the independent group, thirteen (65%, 13/20) studies were conducted in hospital settings (Eack, Greeno, & Lee, 2006; Fann et al., n.d.; Gelaye et al., 2013; Hyphantis et al., 2011; Inagaki et al.; Khamseh et al., 2011; Persoons, Luyckx, Desloovere, Vandenberghe, & Fischler, n.d.; Picardi et al., 2005; Stafford, Berk, & Jackson, 2007; Thombs, Ziegelstein, & Whooley, 2008; A. W. Thompson et al., 2011; Turner et al., 2012; van Steenbergen-Weijenburg et al., 2010). Of the remaining seven studies, six were conducted in primary care settings (Arroll et al., 2010; Ayalon, Goldfracht, & Bech, 2010; Henkel et al., 2004; Lamers et al., 2008; Lotrakul, Sumrithe, & Saipanish, 2008; Zuithoff et al., 2010) and one in a community sample (Gjerdingen, Crow, McGovern, Miner, & Center, 2009).

In both groups (independent and non-independent studies), the majority of studies validated a translated version of the PHQ-9. Two of the studies authored by developers (28%, 2/7) (Spitzer et al., 1999; Thekkumpurath et al., 2011), and eight (40%, 8/20) independent studies (Arroll et al., 2010; Eack et al., 2006; Fann et al., n.d.; Gjerdingen et al., 2009; Stafford et al., 2007; Thombs et al., 2008; A. W. Thompson et al., 2011; Turner et al., 2012) were conducted in English.

The mean prevalence of major depressive disorder in the group of studies co-authored by PHQ-9 developers was 13.4 (range 6.1% - 29.2%); in the independent group it was 15.5% (range 3.9% - 32.4%). The mean age of patients in the PHQ-9 developers group was 45.75; all but one study had a mean age in the range of 40 to 50 years. In the independent group the mean age was 54.6 (range 29.3 – 75.0), with almost half (8) of the studies reporting a mean age of over 60. The percentage of females in the PHQ-9 developers was 56.8% (range 28.6% - 67.8%) and in the independent group was 59.1 (18% -100%).

All of the non-independent studies used a self-reported PHQ-9, whereas in 7 independent studies (30%, 6/20) the PHQ-9 was administered by a researcher (Ayalon et al., 2010; Fann et al., n.d.; Gelaye et al., 2013; Gjerdingen et al., 2009; Hyphantis et al., 2011; Inagaki et al.). Apart from Muramatsu et al. (2007) all of the non-independent studies used the SCID as a gold standard; the independent studies used a wider range of gold standards including SCAN, CIDI, MINI, and C-DIS, though the SCID was also frequently used by the independent studies as well (45%, 9/20 studies).

For peer review only

Four out of the seven non-independent studies (57%) did not include a conflict of interests statement (Diez-Quevedo et al., n.d.; Gräfe et al., 2004; Muramatsu et al., 2007; Spitzer et al., 1999). Also, four (57%) of the non-independent studies acknowledged funding from Pfizer (Gräfe et al., 2004; Löwe et al., 2004; Muramatsu et al., 2007; Spitzer et al., 1999). Only one study (Muramatsu et al., 2007) acknowledged the collaboration with one of the developers of the PHQ-9.

Of the independent studies, twelve (60%) did not include a conflict of interests statement (Eack et al., 2006; Fann et al., n.d.; Gelaye et al., 2013; Gjerdingen et al., 2009; Henkel et al., n.d.; Hyphantis et al., 2011; Lamers et al., 2008; Lotrakul et al., 2008; Persoons et al., n.d.; Picardi et al., 2005; Stafford et al., 2007; A. W. Thompson et al., 2011). It appears that newer studies were more likely to include a conflict of interest statement, which may reflect a recent change in reporting. Funding was acknowledged by most studies (18/20) and most received funding from academic or/and health research institutions. Two studies received funding from pharmaceutical companies – Lundbeck (Ayalon et al., 2010) and Pfizer (Persoons et al., n.d.) and one study acknowledged that Pfizer Italia provided the Italian version of PHQ-9 and gave the authors permission to use it (Picardi et al., 2005).

Diagnostic test accuracy

Pooled sensitivity and specificity was calculated separately for the independent and non-independent studies. Pooled sensitivity for the non-independent studies of the PHQ-9 was 0.77 (95% CI = 0.70 – 0.84), pooled specificity was 0.94 (95% CI = 0.90 – 0.97) and the pooled diagnostic odds ratio was 64.40 (95% CI = 34.15 – 121.43). Heterogeneity was high (I^2 = 78.9%). Figure 1 represents the summary ROCs for this set of studies.

For peer review only

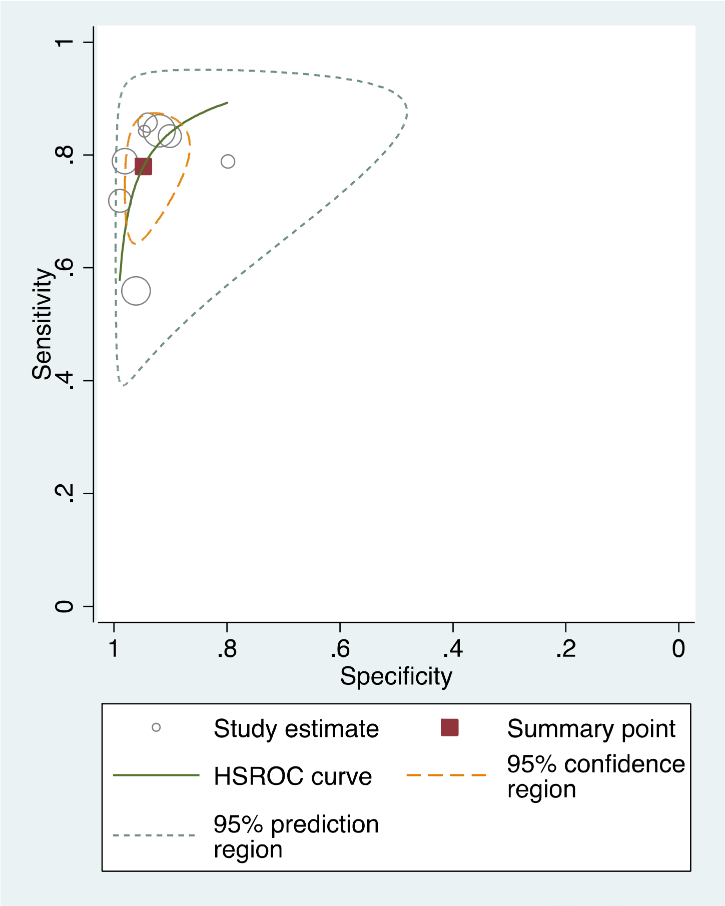

Figure 1. PHQ-9 algorithm scoring method summary ROC plot of diagnosis of major depressive disorder in non-independent studies. Pooled sensitivity and specificity using a bi-variate meta-analysis.

Pooled sensitivity for the independent studies was lower compared to the developer authored studies group at 0.48 (95% CI = 0.41 – 0.91); whereas pooled specificity was the same at 0.94 (95% CI = 0.91 – 0.95). The pooled diagnostic odds ratio was approximately four times lower at 15.05 (95% CI = 11.03 – 20.52) (see figure 2 or sROC). Heterogeneity was substantial at $I^2 = 68.1$ %.

For peer review only - http://bmjopen.bmj.com/site/about/guidelines.xhtml

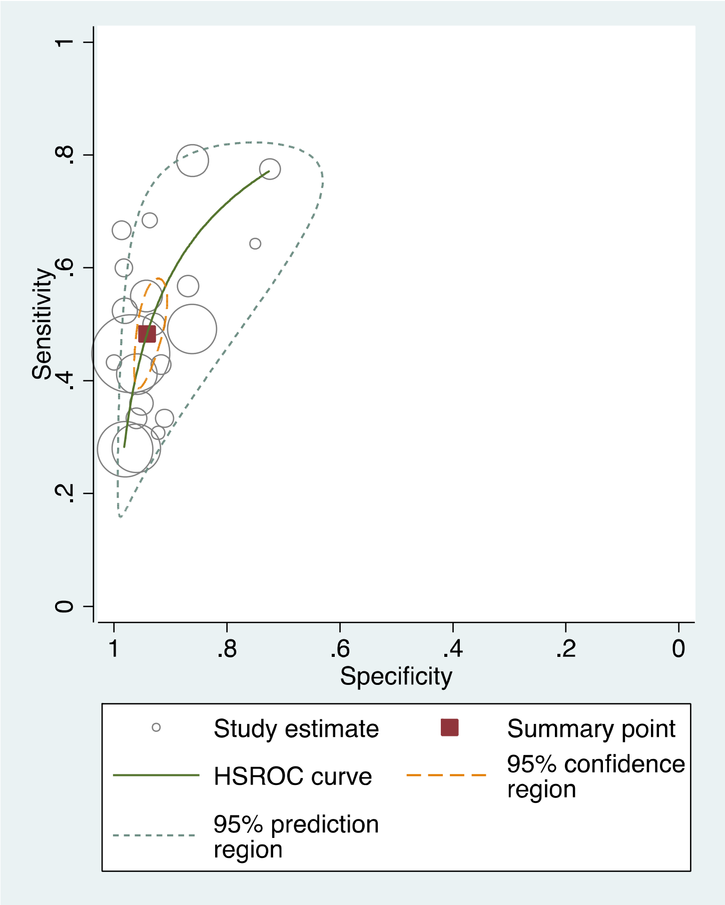

Figure 2. PHQ-9 algorithm scoring method summary ROC plot of diagnosis of major depressive disorder in independent studies. Pooled sensitivity and specificity using a bi-variate meta-analysis.

The meta-regression analysis for algorithm studies with independent status as the predictor of the diagnostic odds ratio showed that independent status was a significant predictor of the diagnostic odds ratio ($p < 0.0001$) and explained a substantial amount of the observed heterogeneity (51.54%).

Quality assessment

The results of the quality assessment using QUADAS-2 are given in table 3 for the studies reporting on the diagnostic performance of the algorithm scoring method. In the patient selection domain, more of the independent studies (65%, 13/20) than the non-independent (29%, 2/7) met the criterion for consecutive referrals. There were no marked differences on the other two criteria in this domain (avoid case-control design, avoid inappropriate exclusions). In the index test domain, the proportion of studies reporting that the PHQ-9 was conducted blind to the reference test was comparable between the two groups. There were differences in this domain for those studies using a translated version of the test. All non-English non-independent studies (5/5) used an appropriately translated version of the PHQ-9; whereas just over a half of the independent studies reported this (55%, 6/11). However, the majority of both sets of studies did not report details of psychometric properties of the translated version. For the reference test domain, nearly all studies in both groups were rated as using a reference test that would correctly classify the condition. While most studies conducted by the developers of the PHQ-9 reported that the reference test was interpreted blind to the PHQ-9 score (86%, 6/7), this was reported in only 60% (12/20) of the independent studies.

The two sets of studies that used translated versions of the reference test were broadly comparable. There was a slight indication that the non-independent studies were more likely to use an appropriately translated version of the reference test and report data on the psychometric properties of the translated version, though the numbers for the translated comparison are very low. There were, however, some more notable differences on the flow and timing domain. Most of the studies conducted by the developers ensured that the time between the index and reference test was under two weeks (86%, 6/7) in comparison to 70% (14/20) of the independent studies. More non-independent studies met the criterion for 'all participants included in the analysis' (57%, 4/7) than the independent studies (25%).

Summed items scoring method (cut-off point 10)

Descriptive characteristics

Table 2 presents the sample characteristics of the thirty-one PHQ-9 validation studies that reported the psychometric properties of the PHQ-9 at cut-off point 10. Five of these studies

For peer review only

were co-authored by the original developers of the instrument or acknowledged collaboration (Gräfe et al., 2004; Kroenke et al., 2001; Thekkumpurath et al., 2011; Williams et al., 2005) or were co-authored by the first author of a previous study that had also been co-authored by one of the developers (Navinés et al., 2012). Twenty-six studies were conducted by independent researchers.

Three (60%, 3/5) of the non-independent studies (Navinés et al., 2012; Thekkumpurath et al., 2011; Williams et al., 2005) and eleven independent studies (42%, 11/26) (Chagas et al., 2013; Elderon, Smolderen, Na, & Whooley, 2011; Fann et al., n.d.; Gelaye et al., 2013; Hyphantis et al., 2011; Khamseh et al., 2011; Rooney et al., 2013; Stafford et al., 2007; Thombs et al., 2008; Watnick, Wang, Demadura, & Ganzini, 2005; Zhang et al., 2013) were conducted in hospital settings.

Three (60%, 3/5) non-independent studies(Kroenke et al., 2001; Thekkumpurath et al., 2011; Williams et al., 2005) and thirteen independent studies (13/26) (Adewuya, Ola, & Afolabi, 2006; Arroll et al., 2010; Elderon et al., 2011; Fann et al., n.d.; Fine et al., 2013; Gilbody, Richards, Brealey, & Hewitt, 2007; Gjerdingen et al., 2009; Phelan et al., 2010; Rooney et al., 2013; Sidebottom, Harrison, Godecker, & Kim, 2012; Stafford et al., 2007; Thombs et al., 2008; Watnick et al., 2005), were conducted in English.

The mean prevalence of major depressive disorder in the group of studies authored by PHQ-9 developers was 13.2% (range 6.1% - 33.5%) and in the independent group was 16.1% (range 2.5% - 43.2%). The mean age of patients in the PHQ-9 developers group studies was 48.1 (range 41.9 -61.0) and in the 26 independent studies that reported these data was 49.1 (range 23.0 – 78.0). The percentage of females in the PHQ-9 developers studies that reported these data(Gräfe et al., 2004; Kroenke et al., 2001; Navinés et al., 2012; Thekkumpurath et al., 2011) was 56.3% (range 28.6% – 67.8%) and in the independent group was 64.9 % (range 12% -100%).

For peer review only

Three of the non-independent studies used the self-reported mode of administration and two of them did not specify how the PHQ-9 was administered. In 9 independent studies (34%, 9/26) the PHQ-9 was administered by the researcher (de Lima Osório, Vilela Mendes, Crippa, & Loureiro, 2009; Fann et al., n.d.; Fine et al., 2013; Gelaye et al., 2013; Gjerdingen et al., 2009; Hyphantis et al., 2011; Patel et al., 2008; Phelan et al., 2010; Sidebottom et al., 2012). All studies authored by developers used SCID as a gold standard; the independent studies used a wider range of gold standards including SCAN, CIDI, MINI, CIS-R, C-DIS, though the SCID was used in half of the studies (50%, 13/26 studies).

Three non-independent studies (60%) did not include a conflict of interests statement (Gräfe et al., 2004; Kroenke et al., 2001; Williams et al., 2005). Two of these studies (Gräfe et al., 2004; Kroenke et al., 2001) acknowledged funding from Pfizer. None of the non-independent studies acknowledged collaboration or authorship of one of the developers of the PHQ-9.

Of the independent studies, thirteen (42%) did not include a conflict of interests statement (Adewuya et al., 2006; Arroll et al., 2010; Azah et al., 2005; de Lima Osório et al., 2009; Fann et al., n.d.; Gelaye et al., 2013; Gjerdingen et al., 2009; Hyphantis et al., 2011; Liu et al., 2011; Lotrakul et al., 2008; Stafford et al., 2007; Watnick et al., 2005; Wittkampf et al., 2009). Similar to the algorithm studies, the newer studies were more likely to include a conflict of interest statement. Funding was acknowledged by most studies (27/31) and most received funding from academic or/and health research institutions. One study (Gilbody et al., 2007) acknowledged that the last author involved in the development of one of the instruments (CORE-OM), 'but does not gain financially from its use'. One study (Elderon et al., 2011) acknowledged funding from industry, AHA Pharmaceuticals Roundtable, but stated that 'the funding organisations had no role in the design or conduct of the study, collection, management, analysis or interpretation of data; or preparation, review or approval of the manuscript. Fine et al., 2013 disclosed that the last author had financial and consulting interests (Pfizer was not cited as one of them).

Diagnostic test accuracy

Pooled sensitivity for the studies linked to the developers of the PHQ-9 was 0.87 (95% CI = 0.77 – 0.93), pooled specificity was 0.87 (95% CI = 0.76 – 0.94) and the pooled diagnostic odds ratio was 49.31 (95% CI = 25.74 – 94.48) – see table 5. Heterogeneity was moderate (I^2 = 55.1%). Figure 4 represents the summary ROCs for this group.

For peer review only

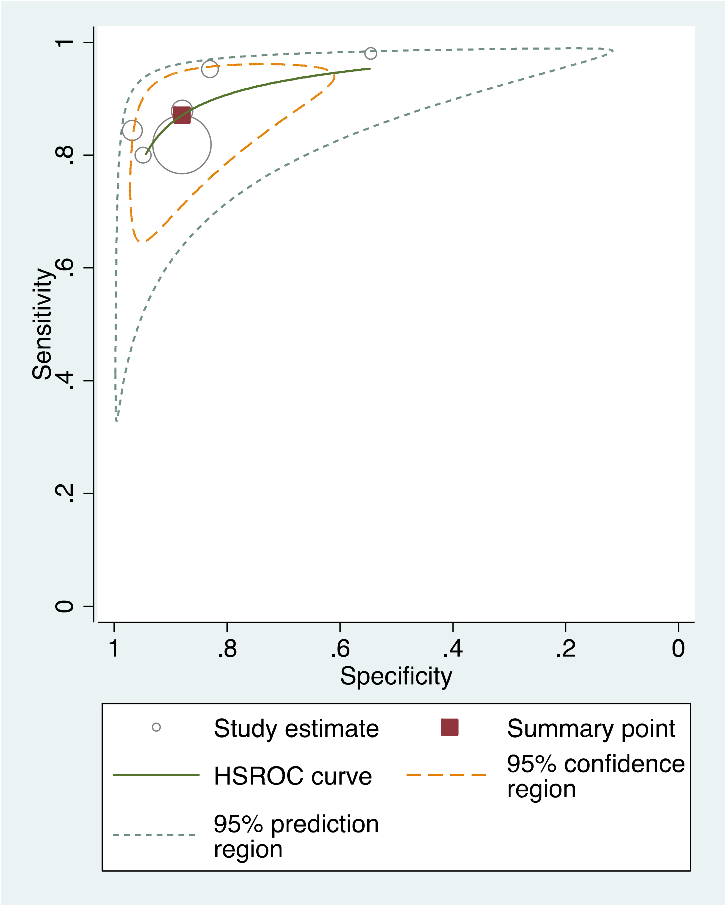

Figure 3. PHQ-9 summed items scoring method at cut-off point 10 summary ROC plot of diagnosis of major depressive disorder in non-idependent studies. Pooled sensitivity and specificity using a bi-variate meta-analysis.

Pooled sensitivity for the independent studies was 0.76 (95% CI, 0.67 – 0.83), pooled specificity was 0.88 95% CI (0.85 – 0.91) and the pooled diagnostic odds ratio was 24.96 (95% CI 14.81 – 42.08), approximately half that of the non-independent studies (table 5). Heterogeneity was high at $I^2 = 81.5$ %. Figure 5 represents the summary ROCs for this group.

For peer review only - http://bmjopen.bmj.com/site/about/guidelines.xhtml

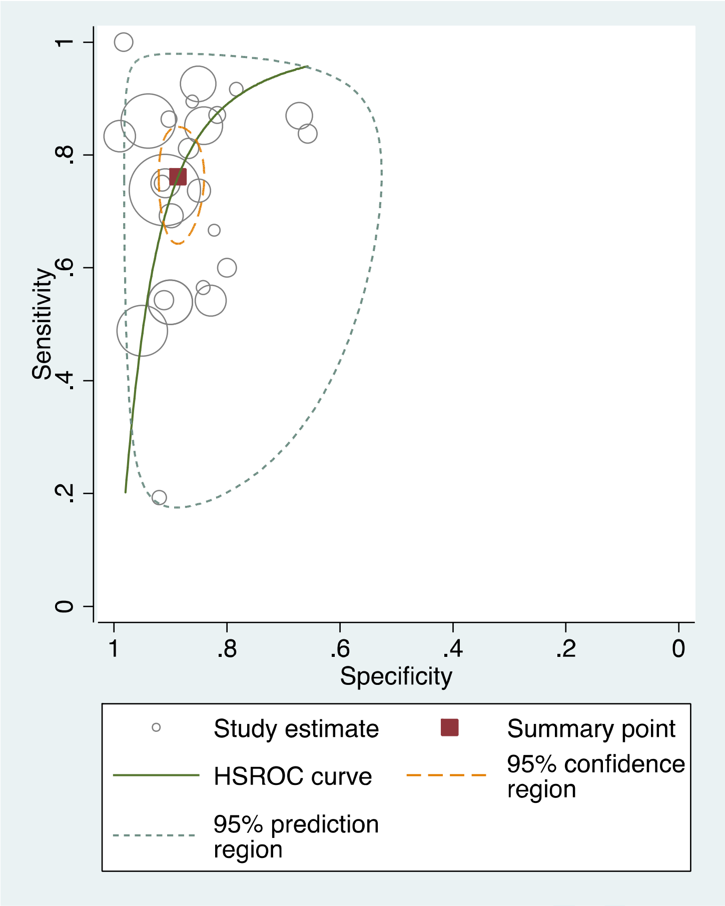

Figure 4. PHQ-9 summed items scoring method at cut-off point 10 summary ROC plot of diagnosis of major depressive disorder in independent studies. Pooled sensitivity and specificity using a bi-variate meta-analysis.

The meta-regression for the studies using a cut-off point of 10 with allegiance status of the predictor showed that allegiance status was a significant predictor of the diagnostic odds ratio (P = 0.015) and explained 18.95% of observed heterogeneity.

Quality assessment

The results of the quality assessment using the QUADAS-2 are given in table 4. For the patient selection domain, the two groups of studies were broadly comparable on two items (consecutive or random sample, avoid case-control design). However, all of the studies from the non-independent studies were rated as avoiding inappropriate exclusions (5/5) in contrast to 58% (15/26) of the independent studies.

On the index test domain, there were a number of differences between the two groups of studies. More of the independent studies (81%, 21/26) reported that the PHQ-9 was interpreted blind to the reference test compared to 60% (3/5) of the studies conducted by the developers of the PHQ-9. All (5/5) of the studies from the PHQ-9 developers were rated as pre-specifying the threshold on the PHQ-9 compared to 73% (19/26) of the independent studies. The two sets of studies were broadly comparable in terms of two items from the reference test domain (correctly classify target condition, reference test interpreted blind). Only one of the studies from the developers of the PHQ-9 used a translated version of the index test or reference test, so it is not possible to comment on differences between the two sets of studies in terms of these items from the index or reference test domains. For the flow and timing domain, the two groups of studies were broadly comparable for two of the criteria (interval of two weeks or less, all participants receive same reference test). However, fewer than half of the independent studies met the criterion for ‘all participants included in the analysis’ (42%, 11/26); whereas all of the studies by the developers of the PHQ-9 met this criterion.

Discussion

This is to our knowledge the first systematic examination of a possible ‘allegiance’ or authorship effect in the validation of screening or case finding psychological instrument for a

For peer review only

common mental health disorder. We reviewed diagnostic validation studies of the PHQ-9, a widely used depression screening-instrument. We found that non-independent studies reported higher sensitivity paired with similar specificity compared to studies conducted by independent researchers. When entered as a covariate in meta-regression analyses, independence status was predictive of variation in the DOR for both the algorithm scoring method and the summed-item scoring method at a cut-off point of 10.

Previous research has proposed several possible explanations for the allegiance effect (Blair et al., 2008; Lilienfeld & Jones, 2008; Singh et al., 2013). One possibility is the advertent bias that may serve to inflate the performance of a test when evaluated by those who have developed it. However, before concluding that the differences are due to this, it is important to explore and rule out alternative explanations. First, it is possible that any observed differences are a result of differences in study characteristics of the two sets of studies (e.g., setting, clinical population). Secondly, differences in the methodological quality of the studies may also account for any differences. These possibilities are examined below.

Difference in study characteristics as potential alternative explanations

The two sets of studies were broadly comparable in terms of gender and the prevalence of depression, so these variables are unlikely to offer an explanation for the differences. While there were some indications from both sets of comparisons that the PHQ-9 may have been researcher-administered more often in the independent studies, it is not immediately clear how this would lead to lowered diagnostic performance.

The diagnostic meta-analyses of the PHQ-9 (Manea et al., 2015; Moriarty et al., 2015) have shown that the sensitivity and DOR of the PHQ-9 tends to be lower in hospital settings for both algorithm and summed-item scoring methods. Whilst the fact that proportionally more independent algorithm studies were conducted in secondary care could explain the lower sensitivity and DOR values in the algorithm studies, in the studies that reported the cut-off point of 10 this would not be the case as proportionally more studies authored by developers were conducted in hospital settings.

For peer review only

Similarly, differences in the proportions of studies using translated versions of the PHQ-9 are also unlikely to offer an obvious explanation of the difference in diagnostic performance, because in the algorithm set of studies more of the non-independent studies used a translated version of the test, but the proportions were in the opposite direction for the studies using a cut off of 10. A similar conclusion is also likely to apply to the age of the samples. There were more older adults studies in the independent than non-independent studies in the algorithm comparison. Depression could be more difficult to identify in older adults due to physical co-morbidities that may present with similar symptomatology to depression and could account for the lower diagnostic performance in the independent studies. However, the independent samples in the studies that reported the psychometric properties at cut-off point 10 had younger samples than the non-independent studies, so this would not support this interpretation.

The SCID was used as the gold standard in nearly all of the non-independent studies. The fact that some independent studies used other gold standards could potentially explain the poorer psychometric properties of the PHQ-9 in these studies. The SCID is often regarded as the most valid of the available semi-structured interviews used in depression diagnostic validity studies as the reference standard. If we assume that this is the case and, furthermore, that the PHQ-9 is an accurate method of screening for depression, then the PHQ-9 may be more likely to agree with the SCID than other reference standards.

Differences in methodological quality as potential alternative explanations

The quality of the studies was evaluated using the QUADAS-2. Although there were several potential methodological differences between the two groups of studies from the algorithm papers, not all of these offer obvious explanations of the observed differences and some are unlikely as explanations. For example, more of the studies from the developers of the PHQ-9 ensured that the reference test was interpreted blind to the index test. This is unlikely to account for the observed differences, because a lack of blinding is typically associated with artificially increased diagnostic performance, which is in the opposite direction to the pattern of results observed here. The impact of some other differences is less clear-cut. For example,

For peer review only

a higher number of the independent studies met the criterion for consecutive referrals. For this to provide an explanation of the of the observed differences, the non-consecutive nature of the referrals in the studies by those who had developed the PHQ-9 would need to have led to the over-inclusion of true positives or under-inclusion of false negatives given that these studies tended to report higher sensitivity relative to the independent studies (and vice versa for the independent studies). It is not immediately obvious how this would occur. The studies by the developers of the PHQ-9 were more likely to have met the criterion of 'included all participants in the analysis'. It is possible that the greater loss of participants from the independent studies may have artificially reduced the observed diagnostic accuracy, though, again, it is not immediately obvious how this would have affected the true positive and false negative rates. Although there is not an obvious explanation of how these differences in methodological quality could account for the observed differences in diagnostic performance, it is important to recognise that they cannot on that basis be ruled out.

There are, however, two differences in methodological quality among the algorithm studies that are clearer potential alternative explanations. The higher rate of appropriate translations among the studies conducted by the developers of the PHQ-9 is potentially important, because lower diagnostic estimates may be expected from studies that have poorly translated versions of the index test. In the flow and timing domain, more of the studies by the developers of the PHQ-9 ensured that there was a less than two-week interval between the index and reference test. This is consistent with lower diagnostic performance in the independent studies: as the interval increases it is likely that depression status may change and this would lead to lower levels of agreement between the index test and the reference test.

There were also differences on some quality assessment items between the two sets of studies in the summed item scoring method comparison. The threshold was reported as pre-specified in all of the studies by the developers of the PHQ-9 in contrast to approximately three quarters of the independent studies. On the face of it, this is unlikely to explain the observed differences, because the use of a pre-specified cut-off point is likely to be associated with lower not higher diagnostic test performance. One possibility, however, is that studies that performed poorly at this cut-off point were less likely to be reported by those who had

For peer review only

developed the measure. As discussed in more detail in the limitations section, we were unable to explore this possibility through the use of formal tests for publication bias.

All non-independent studies avoided inappropriate exclusions compared to approximately half of the independent studies. While this is a potential alternative explanation of the differences it is not immediately obvious how this would explain the differences in diagnostic performance between the two sets of studies. Fewer than half of the independent studies met the criterion for 'all participants included in the analysis' in contrast to all of the studies by the developers of the PHQ-9 met this criterion, but again this difference should if at usually work against the inclusive studies, not those excluding cases. More of the independent studies reported that the PHQ-9 was interpreted blind to the reference test. This does offer a potential explanation, because the absence of blinding may artificially inflate diagnostic accuracy.

Limitations

The results of this review need to be viewed in the light of the limitations of the primary studies that contributed to the review and the review itself. An important consideration is to establish whether any observed differences between the diagnostic performance of the independent and non-independent studies are better accounted for by study characteristic or methodological differences. Caution, however, is needed in interpreting any differences, because of the small number of non-independent studies in both the algorithm and cut-off 10 comparisons. The small number of non-independent studies also meant that we were also unable to explore the potential role of publication bias in the independent and non-independent studies. At least 10 studies are required to use standard methods of examining publication bias, but the number of non-independent studies in both the algorithm and cut-off 10 comparisons were fewer than this.

Conclusions and implications for further research.

For peer review only

The aims of the review was to investigate whether an allegiance effect is found that leads to an increased diagnostic performance in diagnostic validation studies that were conducted by teams connected to the original developers of the PHQ-9. Our analyses showed that diagnostic studies conducted by independent researchers had lower sensitivity paired with similar specificity compared to studies that were classified as non-independent. This conclusion held for both the algorithm and cut-off 10 studies. We explored a range of possible alternative explanations for the observed allegiance effect including both differences in study characteristics and study quality. A number of potential differences were found, though for some of these it is not clear how they would necessarily account for the observed differences. However, there were a number of differences that offered potential alternative explanations unconnected to allegiance effects. These included the greater use of the SCID in the studies rated as non-independent in both the algorithm and the cut-off 10 studies. In the algorithm studies, the studies rated as non-independent were also more likely to use an appropriate translation of the PHQ-9 and were also more likely to ensure that the index and reference test were conducted within two weeks of each other, both of which may be associated with an improvement in observed diagnostic performance of an instrument. The majority of studies in both meta-analyses did not provide clear statements about potential conflict of interest and/or funding, however the newer studies were more likely to provide such statements, which may reflect increasing transparency in this area of research.

We cannot, therefore, conclude that allegiance effects are present in studies examining the diagnostic performance of the PHQ-9; but nor can we rule them out. Conflicts of interest are an important area of investigation in medical and behavioural research, particularly due to concerns about trial results being influenced by industry sponsorship. Future diagnostic validity in this area should as a matter of routine present clear statements about potential conflicts of interest and funding, particularly relating to the development of the instrument under evaluation. Future meta-analyses of diagnostic validation studies of psychological measures should routinely evaluate the impact of researcher allegiance in the primary studies examined in the meta-analysis.

For peer review only

Contributors LM led on all stages of the review and is the guarantor. We used an established database of diagnostic validation studies of the PHQ-9 (Manea et al., 2015; Moriarty et al., 2015) SG provided expert advice on methodology and approaches to assessment of the evidence base. AM carried out the literature searches, screened the studies, extracted data and assessed the quality of the included studies for one of the systematic reviews (Moriarty et al., 2015) . LM carried out the literature searches, screened the studies, extracted data and assessed the quality of the included studies for the other systematic review (Manea et al., 2015), analysed the data for both systematic reviews and drafted the report. JB involved in the development of the study, wrote the introduction section of the review and contributed to the production of the final report. DM supervised the quality assessment, methodology and approaches to evidence synthesis, provided senior advice and support throughout and contributed to the production of the final report. All parties were involved in drafting and/or commenting on the report.

Competing interests None declared.

Provenance and peer review Not commissioned; externally peer reviewed.

Data sharing statement No additional data are available.

REFERENCES

1. Adewuya, A. O., Ola, B. A., & Afolabi, O. O. (2006). Validity of the patient health questionnaire (PHQ-9) as a screening tool for depression amongst Nigerian university students. *Journal of Affective Disorders*, *96*(1–2), 89–93. http://doi.org/10.1016/j.jad.2006.05.021

2. (2010). Validation of PHQ-2 and PHQ-9 to Screen for Major Depression in the Primary Care Population. *The Annals of Family Medicine*, *8*(4), 348–353. http://doi.org/10.1370/afm.1139

For peer review only

3. Ayalon, L., Goldfracht, M., & Bech, P. (2010). “Do you think you suffer from depression?” Reevaluating the use of a single item question for the screening of depression in older primary care patients. *International Journal of Geriatric Psychiatry*, *25*(5), 497–502. http://doi.org/10.1002/gps.2368

4. Azah, M. N. N., Shah, M. E. M., Juwita, S., Bahri, I. S., Rushidi, W. M. W. M., & Jamil, Y. M. (2005). Validation of the Malay Version Brief Patient Health Questionnaire (PHQ-9) among Adult Attending Family Medicine Clinics. *International Medical Journal*.

5. Blair, P. R., Marcus, D. K., & Boccaccini, M. T. (2008). Is There an Allegiance Effect for Assessment Instruments? Actuarial Risk Assessment as an Exemplar. *Clinical Psychology: Science and Practice*, *15*(4), 346–360. http://doi.org/10.1111/j.1468-2850.2008.00147.x

6. Chagas, M. H. N., Tumas, V., Rodrigues, G. R., Machado-de-Sousa, J. P., Filho, A. S., Hallak, J. E. C., & Crippa, J. A. S. (2013). Validation and internal consistency of Patient Health Questionnaire-9 for major depression in Parkinson’s disease. *Age and Ageing*, *42*(5), 645–649. http://doi.org/10.1093/ageing/aft065

7. de Lima Osório, F., Vilela Mendes, A., Crippa, J. A., & Loureiro, S. R. (2009). Study of the Discriminative Validity of the PHQ-9 and PHQ-2 in a Sample of Brazilian Women in the Context of Primary Health Care. *Perspectives in Psychiatric Care*, *45*(3), 216–227. http://doi.org/10.1111/j.1744-6163.2009.00224.x

8. Validation and utility of the patient health questionnaire in diagnosing mental disorders in 1003 general hospital Spanish inpatients. *Psychosomatic Medicine*, *63*(4), 679–86. Retrieved from http://www.ncbi.nlm.nih.gov/pubmed/11485122

9. Dragioti, E., Dimoliatis, I., & Evangelou, E. (2015). Disclosure of researcher allegiance in meta-analyses and randomised controlled trials of psychotherapy: a systematic appraisal. *BMJ Open*, *5*(6), e007206–e007206. http://doi.org/10.1136/bmjopen-2014-007206

10. Eack, S. M., Greeno, C. G., & Lee, B.-J. (2006). Limitations of the Patient Health Questionnaire in Identifying Anxiety and Depression: Many Cases Are Undetected. *Research on Social Work Practice*, *16*(6), 625–631. http://doi.org/10.1177/1049731506291582

For peer review only

11. Elderon, L., Smolderen, K. G., Na, B., & Whooley, M. A. (2011). Accuracy and prognostic value of American Heart Association: recommended depression screening in patients with coronary heart disease: data from the Heart and Soul Study. *Circulation. Cardiovascular Quality and Outcomes*, *4*(5), 533–40. http://doi.org/10.1161/CIRCOUTCOMES.110.960302

12. Fann, J. R., Bombardier, C. H., Dikmen, S., Esselman, P., Warms, C. A., Pelzer, E., … Temkin, N. (n.d.). Validity of the Patient Health Questionnaire-9 in assessing depression following traumatic brain injury. *The Journal of Head Trauma Rehabilitation*, *20*(6), 501–11. Retrieved from http://www.ncbi.nlm.nih.gov/pubmed/16304487

13. Fine, T. H., Contractor, A. A., Tamburrino, M., Elhai, J. D., Prescott, M. R., Cohen, G. H., … Calabrese, J. R. (2013). Validation of the telephone-administered PHQ-9 against the in-person administered SCID-I major depression module. *Journal of Affective Disorders*, *150*(3), 1001–1007. http://doi.org/10.1016/j.jad.2013.05.029

14. Gelaye, B., Williams, M. A., Lemma, S., Deyessa, N., Bahretibeb, Y., Shibre, T., … Andrew Zhou, X. H. (2013). Validity of the patient health questionnaire-9 for depression screening and diagnosis in East Africa. *Psychiatry Research*, *210*(2). http://doi.org/10.1016/j.psychres.2013.07.015

15. Gilbody, S., Richards, D., Brealey, S., & Hewitt, C. (2007). Screening for depression in medical settings with the Patient Health Questionnaire (PHQ): A diagnostic meta-analysis. *Journal of General Internal Medicine*, *22*, 1596–1602. http://doi.org/10.1007/s11606-007-0333-y

16. Gjerdingen, D., Crow, S., McGovern, P., Miner, M., & Center, B. (2009). Postpartum depression screening at well-child visits: validity of a 2-question screen and the PHQ-9. *Annals of Family Medicine*, *7*(1), 63–70. http://doi.org/10.1370/afm.933

17. Gräfe, K., Zipfel, S., Herzog, W., & Löwe, B. (2004). Screening psychischer Störungen mit dem “Gesundheitsfragebogen für Patienten (PHQ-D)“. *Diagnostica*, *50*(4), 171–181. http://doi.org/10.1026/0012-1924.50.4.171

18. Henkel, V., Mergl, R., Kohnen, R., Allgaier, A.-K., Möller, H.-J., & Hegerl, U. (n.d.). Use of brief depression screening tools in primary care: consideration of heterogeneity in performance in different patient groups. *General Hospital Psychiatry*, *26*(3), 190–8.

For peer review only

http://doi.org/10.1016/j.genhosppsych.2004.02.003

19. Hyphantis, T., Kotsis, K., Voulgari, P. V., Tsifetaki, N., Creed, F., & Drosos, A. A. (2011). Diagnostic accuracy, internal consistency, and convergent validity of the Greek version of the patient health questionnaire 9 in diagnosing depression in rheumatologic disorders. *Arthritis Care & Research*, *63*(9), 1313–1321. http://doi.org/10.1002/acr.20505

20. Inagaki, M., Ohtsuki, T., Yonemoto, N., Kawashima, Y., Saitoh, A., Oikawa, Y., … Yamada, M. Validity of the Patient Health Questionnaire (PHQ)-9 and PHQ-2 in general internal medicine primary care at a Japanese rural hospital: a cross-sectional study. *General Hospital Psychiatry*, *35*(6), 592–7. http://doi.org/10.1016/j.genhosppsych.2013.08.001

21. Khamseh, M. E., Baradaran, H. R., Javanbakht, A., Mirghorbani, M., Yadollahi, Z., & Malek, M. (2011). Comparison of the CES-D and PHQ-9 depression scales in people with type 2 diabetes in Tehran, Iran. *BMC Psychiatry*, *11*(1), 61. http://doi.org/10.1186/1471-244X-11-61

22. Kroenke, K., Spitzer, R. L., & Williams, J. B. (2001). The PHQ-9: validity of a brief depression severity measure. *Journal of General Internal Medicine*, *16*(9), 606–13. Retrieved from http://www.ncbi.nlm.nih.gov/pubmed/11556941

23. Lamers, F., Jonkers, C. C. M., Bosma, H., Penninx, B. W. J. H., Knottnerus, J. A., & van Eijk, J. T. M. (2008). Summed score of the Patient Health Questionnaire-9 was a reliable and valid method for depression screening in chronically ill elderly patients. *Journal of Clinical Epidemiology*, *61*(7), 679–687. http://doi.org/10.1016/j.jclinepi.2007.07.018

24. Lijmer, J. G., Bossuyt, P. M. M., & Heisterkamp, S. H. (2002). Exploring sources of heterogeneity in systematic reviews of diagnostic tests. *Statistics in Medicine*, *21*(11), 1525–1537. http://doi.org/10.1002/sim.1185

25. Lilienfeld, S. O., & Jones, M. K. (2008). Allegiance Effects in Assessment: Unresolved Questions, Potential Explanations, and Constructive Remedies. *Clinical Psychology: Science and Practice*, *15*(4), 361–365. http://doi.org/10.1111/j.1468-2850.2008.00148.x

26. Liu, S.-I., Yeh, Z.-T., Huang, H.-C., Sun, F.-J., Tjung, J.-J., Hwang, L.-C., … Yeh, A. W.-C. (2011). Validation of Patient Health Questionnaire for depression screening

For peer review only

For peer review only - http://bmjopen.bmj.com/site/about/guidelines.xhtml

among primary care patients in Taiwan. *Comprehensive Psychiatry*, *52*(1), 96–101. http://doi.org/10.1016/j.comppsych.2010.04.013

27. Lotrakul, M., Sumrithe, S., & Saipanish, R. (2008). Reliability and validity of the Thai version of the PHQ-9. *BMC Psychiatry*, *8*(1), 46. http://doi.org/10.1186/1471-244X-8-46

28. Löwe, B., Spitzer, R. L., Gräfe, K., Kroenke, K., Quenter, A., Zipfel, S., … Herzog, W. (2004). Comparative validity of three screening questionnaires for DSM-IV depressive disorders and physicians' diagnoses. *Journal of Affective Disorders*, *78*(2), 131–40. Retrieved from http://www.ncbi.nlm.nih.gov/pubmed/14706723

29. Luborsky, L., Diguer, L., Seligman, D. A., Rosenthal, R., Krause, E. D., Johnson, S., … Schweizer, E. (2006). The Researcher's Own Therapy Allegiances: A "Wild Card" in Comparisons of Treatment Efficacy. *Clinical Psychology: Science and Practice*, *6*(1), 95–106. http://doi.org/10.1093/clipsy.6.1.95

30. Manea, L., Gilbody, S., & McMillan, D. (2015). A diagnostic meta-analysis of the Patient Health Questionnaire-9 (PHQ-9) algorithm scoring method as a screen for depression. *General Hospital Psychiatry*, *37*(1), 67–75. http://doi.org/10.1016/j.genhosppsych.2014.09.009

31. Mann, R., Hewitt, C. E., & Gilbody, S. M. (2009). Assessing the quality of diagnostic studies using psychometric instruments: applying QUADAS. *Social Psychiatry and Psychiatric Epidemiology*, *44*(4), 300–307. http://doi.org/10.1007/s00127-008-0440-z

32. Markman, K. D., & Hirt, E. R. (2002). Social Prediction and the "Allegiance Bias." *Social Cognition*, *20*(1), 58–86. http://doi.org/10.1521/soco.20.1.58.20943

33. McLeod, J. (2010). Taking allegiance seriously–implications for research policy and practice. *European Journal of Psychotherapy and Counselling*. Retrieved from http://www.tandfonline.com/doi/abs/10.1080/13642531003637791?journalCode=rejp20#.WC8nwktA-ao.mendeley

34. Moriarty, A. S., Gilbody, S., McMillan, D., & Manea, L. (2015). Screening and case finding for major depressive disorder using the Patient Health Questionnaire (PHQ-9): a meta-analysis. *General Hospital Psychiatry*, *37*(6), 567–576. http://doi.org/10.1016/j.genhosppsych.2015.06.012

For peer review only

35. Munder, T., Brütsch, O., Leonhart, R., Gerger, H., & Barth, J. (2013). Researcher allegiance in psychotherapy outcome research: An overview of reviews. *Clinical Psychology Review*, *33*(4), 501–511. http://doi.org/10.1016/j.cpr.2013.02.002

36. Muramatsu, K., Miyaoka, H., Kamijima, K., Muramatsu, Y., Yoshida, M., Otsubo, T., & Gejyo, F. (2007). The patient health questionnaire, Japanese version: validity according to the mini-international neuropsychiatric interview-plus. *Psychological Reports*, *101*(3 Pt 1), 952–60. http://doi.org/10.2466/pr0.101.3.952-960

37. Navinés, R., Castellví, P., Moreno-España, J., Gimenez, D., Udina, M., Cañizares, S., … Martín-Santos, R. (2012). Depressive and anxiety disorders in chronic hepatitis C patients: Reliability and validity of the Patient Health Questionnaire. *Journal of Affective Disorders*, *138*(3), 343–351. http://doi.org/10.1016/j.jad.2012.01.018

38. Patel, V., Araya, R., Chowdhary, N., King, M., Kirkwood, B., Nayak, S., … Weiss, H. A. (2008). Detecting common mental disorders in primary care in India: a comparison of five screening questionnaires. *Psychological Medicine*, *38*(2). http://doi.org/10.1017/S0033291707002334

39. Persoons, P., Luyckx, K., Desloovere, C., Vandenberghe, J., & Fischler, B. (n.d.). Anxiety and mood disorders in otorhinolaryngology outpatients presenting with dizziness: validation of the self-administered PRIME-MD Patient Health Questionnaire and epidemiology. *General Hospital Psychiatry*, *25*(5), 316–23. Retrieved from http://www.ncbi.nlm.nih.gov/pubmed/12972222

40. Phelan, E., Williams, B., Meeker, K., Bonn, K., Frederick, J., LoGerfo, J., & Snowden, M. (2010). A study of the diagnostic accuracy of the PHQ-9 in primary care elderly. *BMC Family Practice*, *11*(1), 63. http://doi.org/10.1186/1471-2296-11-63

41. Picardi, A., Adler, D. A., Abeni, D., Chang, H., Pasquini, P., Rogers, W. H., & Bungay, K. M. (2005). Screening for depressive disorders in patients with skin diseases: a comparison of three screeners. *Acta Dermato-Venereologica*, *85*(5), 414–9. http://doi.org/10.1080/00015550510034966

42. Reitsma, J. B., Glas, A. S., Rutjes, A. W. S., Scholten, R. J. P. M., Bossuyt, P. M., & Zwinderman, A. H. (2005). Bivariate analysis of sensitivity and specificity produces informative summary measures in diagnostic reviews. *Journal of Clinical Epidemiology*,

For peer review only

58(10), 982–990. http://doi.org/10.1016/j.jclinepi.2005.02.022

43. Rooney, A. G., McNamara, S., Mackinnon, M., Fraser, M., Rampling, R., Carson, A., & Grant, R. (2013). Screening for major depressive disorder in adults with cerebral glioma: an initial validation of 3 self-report instruments. *Neuro-Oncology*, *15*(1), 122–129. http://doi.org/10.1093/neuonc/nos282

44. Sidebottom, A. C., Harrison, P. A., Godecker, A., & Kim, H. (2012). Validation of the Patient Health Questionnaire (PHQ)-9 for prenatal depression screening. *Archives of Women's Mental Health*, *15*(5), 367–374. http://doi.org/10.1007/s00737-012-0295-x

45.Singh, J. P., Grann, M., & Fazel, S. (2013). Authorship Bias in Violence Risk Assessment? A Systematic Review and Meta-Analysis. *PLoS ONE*, *8*(9), e72484. http://doi.org/10.1371/journal.pone.0072484

46. Spitzer, R. L., Kroenke, K., & Williams, J. B. (1999). Validation and utility of a self-report version of PRIME-MD: the PHQ primary care study. Primary Care Evaluation of Mental Disorders. Patient Health Questionnaire. *JAMA*, *282*(18), 1737–44. Retrieved from http://www.ncbi.nlm.nih.gov/pubmed/10568646

47. Stafford, L., Berk, M., & Jackson, H. J. (2007). Validity of the Hospital Anxiety and Depression Scale and Patient Health Questionnaire-9 to screen for depression in patients with coronary artery disease. *General Hospital Psychiatry*, *29*(5), 417–424. http://doi.org/10.1016/j.genhosppsych.2007.06.005

48. Staines, G. L., & Cleland, C. M. (2007). Bias in meta-analytic estimates of the absolute efficacy of psychotherapy. *Review of General Psychology*, *11*(4), 329–347. http://doi.org/10.1037/1089-2680.11.4.329

49. Thekkumpurath, P., Walker, J., Butcher, I., Hodges, L., Kleiboer, A., O'Connor, M., … Sharpe, M. (2011). Screening for major depression in cancer outpatients: the diagnostic accuracy of the 9-item patient health questionnaire. *Cancer*, *117*(1), 218–27. http://doi.org/10.1002/cncr.25514

50. Thombs, B. D., Ziegelstein, R. C., & Whooley, M. A. (2008). Optimizing detection of major depression among patients with coronary artery disease using the patient health questionnaire: data from the heart and soul study. *Journal of General Internal Medicine*, *23*(12), 2014–7. http://doi.org/10.1007/s11606-008-0802-y

For peer review only

51. Thompson, A. W., Liu, H., Hays, R. D., Katon, W. J., Rausch, R., Diaz, N., … Vickrey, B. G. (2011). Diagnostic accuracy and agreement across three depression assessment measures for Parkinson's disease. *Parkinsonism & Related Disorders*, *17*(1), 40–45. http://doi.org/10.1016/j.parkreldis.2010.10.007

52. Thompson, S. G., & Higgins, J. P. T. (2002). How should meta-regression analyses be undertaken and interpreted? *Statistics in Medicine*, *21*(11), 1559–1573. http://doi.org/10.1002/sim.1187

53. Turner, A., Hambridge, J., White, J., Carter, G., Clover, K., Nelson, L., & Hackett, M. (2012). Depression screening in stroke: a comparison of alternative measures with the structured diagnostic interview for the diagnostic and statistical manual of mental disorders, fourth edition (major depressive episode) as criterion standard. *Stroke; a Journal of Cerebral Circulation*, *43*(4), 1000–5. http://doi.org/10.1161/STROKEAHA.111.643296

54. van Steenbergen-Weijenburg, K. M., de Vroege, L., Ploeger, R. R., Brals, J. W., Vloedbeld, M. G., Veneman, T. F., … van der Feltz-Cornelis, C. M. (2010). Validation of the PHQ-9 as a screening instrument for depression in diabetes patients in specialized outpatient clinics. *BMC Health Services Research*, *10*(1), 235. http://doi.org/10.1186/1472-6963-10-235

55. Walter, S. D. (2002). Properties of the summary receiver operating characteristic (SROC) curve for diagnostic test data. *Statistics in Medicine*, *21*(9), 1237–1256. http://doi.org/10.1002/sim.1099

56. Walters, G. D. (2009). The Psychological Inventory of Criminal Thinking Styles and Psychopathy Checklist: Screening version as incrementally valid predictors of recidivism. *Law and Human Behavior*, *33*(6), 497–505. http://doi.org/10.1007/s10979-008-9167-3

57. Watnick, S., Wang, P.-L., Demadura, T., & Ganzini, L. (2005). Validation of 2 depression screening tools in dialysis patients. *American Journal of Kidney Diseases : The Official Journal of the National Kidney Foundation*, *46*(5), 919–24. http://doi.org/10.1053/j.ajkd.2005.08.006

58. Whiting, P. F., Rutjes, A. W. S., Westwood, M. E., Mallett, S., Deeks, J. J., Reitsma, J.

For peer review only

For peer review only - http://bmjopen.bmj.com/site/about/guidelines.xhtml

B., … QUADAS-2 Group. (2011). QUADAS-2: a revised tool for the quality assessment of diagnostic accuracy studies. *Annals of Internal Medicine*, *155*(8), 529–36. http://doi.org/10.7326/0003-4819-155-8-201110180-00009

59. Williams, L. S., Brizendine, E. J., Plue, L., Bakas, T., Tu, W., Hendrie, H., & Kroenke, K. (2005). Performance of the PHQ-9 as a screening tool for depression after stroke. *Stroke; a Journal of Cerebral Circulation*, *36*(3), 635–8. http://doi.org/10.1161/01.STR.0000155688.18207.33

60. Winter, D. A. (2010, May 7). Editorial. Routledge. Retrieved from http://www.tandfonline.com/doi/abs/10.1080/13642531003637726?needAccess=true&journalCode=rejp20#.WC8nUSu6MP8.mendeley

61. Wittkampf, K., van Ravesteijn, H., Baas, K., van de Hoogen, H., Schene, A., Bindels, P., … van Weert, H. (2009). The accuracy of Patient Health Questionnaire-9 in detecting depression and measuring depression severity in high-risk groups in primary care. *General Hospital Psychiatry*, *31*(5), 451–459. http://doi.org/10.1016/j.genhosppsych.2009.06.001

62. Zhang, Y., Ting, R., Lam, M., Lam, J., Nan, H., Yeung, R., … Chan, J. C. N. (2013). Measuring depressive symptoms using the Patient Health Questionnaire-9 in Hong Kong Chinese subjects with type 2 diabetes. *Journal of Affective Disorders*, *151*(2), 660–666. http://doi.org/10.1016/j.jad.2013.07.014

63. Zuithoff, N. P., Vergouwe, Y., King, M., Nazareth, I., van Wezep, M. J., Moons, K. G., & Geerlings, M. I. (2010). The Patient Health Questionnaire-9 for detection of major depressive disorder in primary care: consequences of current thresholds in a crosssectional study. *BMC Family Practice*, *11*(1), 98. http://doi.org/10.1186/1471-2296-11-98

For peer review only

Table 1: Descriptive characteristics of algorithm studies (Manea et al., 2014)

Study	Sample characteristics (Country, setting, age, sex)	Sample size and % depressed	PHQ-9 characteristics	Diagnostic standard	a) Conflict of interest (COI) declaration<br>b) Funding<br>c) Relationship with original developers
Diez-Quevedo et al. (2001)	Country: Spain	N = 1003	Administration: Self-report	DSM-III-R	a) a) No COI declaration<br>b) Funding acknowledged (academic institutions)<br>c) Not acknowledged
	Setting: Medical and surgical tertiary hospitals	Depressed: 8.2%	Language: Spanish	SCID	
	Age (yrs): M=43 (SD=14.2)				
	Female: 45.6%				
Gräfe et al. (2004)	Country: Germany	N = 528	Language: German	DSM-IV	a) No COI declaration<br>b) Acknowledged funding from Pfizer<br>c) Not acknowledged

For peer review only

	Setting: psychosomatic walk-in clinics and family practices<br><br>Age (yrs): M = 41.9 (SD = 13.8)<br><br>Female: 67.8%	Depressed: 29.2% psychosomatic patients; 6.16% medical patients	Administration: self-report	SCID	
Lowe et al. (2004)	Country: Germany	N = 501	Administration: Self-report	DSM–IV	a) COI declaration 'This study was supported by unrestricted restricted grants from Pfizer Germany and from the medical faculty of the University of Heidelberg Germany, and there are no COI.'<br>b) Acknowledged funding from Pfizer and academic institution<br>c) Not acknowledged
	Setting: Outpatient clinics and family practices	Depressed: 13.2%	Language: German	SCID	

For peer review only

	Age (yrs): M = 41.7 (SD = 13.8)<br>Female: 67.1%				
Muramatsu et al. (2007)	Country: Japan	N = 131	Administration: Self-report	DSM–IV	a) No COI declaration<br>b) Acknowledged funding from Pfizer<br>c) Acknowledged one of the developers of the PHQ-9: 'The authors acknowledge Dr R L Spitzer'
	Setting: Primary care and general hospital	Depressed: 28.2%	Language: Japanese	MINI	
	Age (yrs): M = 43.3 (SD = 16.4)<br>Female: 59.5%				
Navinés et al. (2012)	Country: Spain	N = 500	Administration: Self-report	DSM–IV	a) All authors declared that they had no COI.<br>b) Role of funding source declared<br>c) Not acknowledged

For peer review only

	Setting: General hospital (patients with chronic HCV)	Depressed: 6.4%	Language: Spanish	SCID	
	Age (yrs): M = 43.4 (SD = 10.2)				
	Female: 28.6%				
Spitzer et al. (1999)	Country: US	N = 3000 (585 received SCID)	Administration: Self-report	DSM-III-R	a) No COI declaration<br>b) Acknowledged funding from Pfizer. 'Drs Spitzer and Williams receive honoraria and consulting money from Pfizer Inc, which has supported this work.'<br>c) N/A
	Setting: Primary care	Depressed: 10%	Language: English	SCID	
	Age (yrs): M = 46 (SD = 17.2)				
	Female: 66%				

For peer review only

Thekkumpurath et al. (2010)	Country: UK<br>Setting: Hospital (cancer patients)<br>Age (yrs): M = 61<br>Female: 63%	N = 782<br>Depressed: 6.3% (of the whole sample)	Administration: Not stated<br>Language: English	DSM-IV<br>SCID	a) COI declaration: ‘Supported by Cancer Research UK’<br>b) As in a)<br>c) Not acknowledged
Ayalon et al. (2010)	Country: Israel	N = 153	Administration: Researcher administered	DSM-IV	a) COI declaration: ‘The project was funded by an Investigator’s Initiated Research Grant from Lundbeck International given to Dr Liat Ayalon. Lundbeck International had no other involvement in the project concept of design or in this paper. Per Bech has occasionally over the past 3 years until August 2008 received funding from and has been speaker or member of advisory boards for pharmaceutical companies with an interest in the drug treatment of affective disorders (Astra-Zeneca, Lilly, H. Lundbeck A/S, Lundbeck Foundation and Organon). ‘<br>b) Acknowledged funding from Lundbeck International

For peer review only

	Age (yrs): M = 75 (SD = 8.1)	Depressed: 3.9 %	Language: Hebrew	SCID	
	Female: 40.5 %				
	Country: US	N = 50	Administration: Self-report	DSM-IV	a) No COI declaration<br>b) Funding acknowledged (academic /health research institutions)
Eack et al. (2006)	Setting: Community mental health centers for children	Depressed: 28%	Language: English	SCID	
	Age (yrs): M = 39.20 (SD 9.63)				
	Female: 100%				
Fann et al. (2005)	Country: US	N = 135	Administration: Telephone-administered	DSM-IV	a) No COI declaration<br>b) Funding acknowledged (academic institutions)

For peer review only

	Setting: Trauma hospital (inpatients with traumatic brain injury)	Depressed: 16.3%	Language: English	SCID	
	Age (yrs): M = 42 (SD=17.9)				
	Female: 29.1%				
Gelaye et al. (2011)	Country: Ethiopia	N = 363	Administration: Researcher-administered	DSM-IV	a) No COI declaration<br>b) Funding acknowledged (academic /health research institutions)
	Setting: General hospital	Depressed: 12.6%	Language: Amharic	SCAN	
	Age (yrs): 34.9 (SD=11.6)				
	Female: 63.1 %				

For peer review only

Gjerdingen et al. (2009)	Country: US	N = 438	Administration: Telephone or self-report	DSM-IV	a) No COI declaration<br>b) Funding acknowledged (academic /health research institutions)
	Setting: Community	Depressed: 4.6%	Language: English	SCID	
	Age (yrs): M = 29.3				
	Female: 100%				
Henkel et al. (2004)	Country: Germany	N = 448	Administration: self-report	DSM-IV	a) No COI declaration<br>b) Funding acknowledged (academic /health research institutions)
	Setting: primary care	Depressed: 10%		CIDI	
	Age (yrs): not reported		Language: German		
	Female: 74%				

For peer review only

					a) No COI declaration<br>b) No funding acknowledgement
Hyphantis et al. (2011)	Country: Greece	N = 213	Administration: Researcher administered	DSM-IV	
	Setting: Hospital – rheumatology patients	Depressed: 32.4%	Language: Greek	MINI	
	Age (yrs): M = 54.2 (SD = 13.5)				
	Female: 74%				
Inagaki et al. (2013)	Country: Japan	N = 104 out of 511 received MINI	Administration: Researcher administered	DSM-IV	a) COI declaration: 'The authors declare that they have no competing interests.'<br>b) Funding acknowledged (academic /health research institutions)
	Setting: General hospital	Depressed: 7.4%	Language: Japanese	MINI	
	Age whole sample (yrs): M = 73.5 (SD = 12.3)				

For peer review only

	Female: 59.3%				
Khamseh et al. (2011)	Country: Iran	N = 185	Administration: Self report	DSM-IV	a) COI declaration: The authors declared no competing interests<br>b) Funding acknowledged (academic /health research institutions)
	Setting: Diabetes clinic	Depressed: 43.2%	Language: Persian	SCID	
	Age (yrs): M = 56.17 (SD = 9.60)				
	Female: 51.9%				
Lamers et al. (2008)	Country: Netherlands	N = 713	Administration: Self report	DSM-IV	a) No COI declaration<br>b) Funding acknowledged (academic /health research institutions)
	Setting: Primary care (elderly)	Depressed: 10.7%	Language: Dutch	MINI	
	Age (yrs): M = 71.4 (SD = 6.90)				
	Female: 48.2%				

For peer review only

Lotrakul et al. (2008)	Country: Thailand<br><br>Setting: Primary care<br><br>Age (yrs): M = 45.0 (SD = 14.30)<br><br>Female: 73.7%	N = 279<br><br>Depressed: 6.8%	Administration: Self report<br><br>Language: Thai	DSM-IV<br><br>MINI	a) No COI declaration<br>b) Funding acknowledged (academic /health research institutions)
Persoons et al. (2003)	Country: Belgium<br><br>Setting: Hospital (otolaryngology patients)<br><br>Age (yrs): M = 48.2 (SD = 12.9)	N = 268 (97 received MINI)<br><br>Depressed: 16.5%	Administration: Self-report<br><br>Language: Dutch	DSM-IV<br><br>MINI	a) No COI declaration<br>b) Funding acknowledged (academic /health research institutions) and Pfizer Belgium

For peer review only

	Female: 65.6%				
Picardi et al. (2005)	Country: Italy	N = 141	Administration: Self-report	DSM-IV	a) No COI declaration<br>b) Funding acknowledged (academic /health research institutions). Acknowledged Pfizer Italia SRL for providing the Italian version of the PHQ-9 and for permission to use it.
	Setting: Hospital (dermatology inpatients)	Depressed: 8.5%	Language: Italian	SCID	
	Age (yrs): M = 37.5				
	Female: 56%				
Stafford et al. (2007)	Country: Australia	N = 193	Administration: Self-report	DSM–IV	a) No COI declaration<br>b) Funding acknowledged (academic/health research institutions)
	Setting: Hospital (cardiology patients)	Depressed: 18%	Language: English	MINI	
	Age (yrs): M = 64.1 (SD = 10.3)				

For peer review only

	Female: 66%				
Thombs et al. (2008)	Country: US<br>Setting: Hospital (outpatients with coronary heart disease)<br>Age (yrs): M = 67 (SD = 11)<br>Female: 18%	N = 1024<br>Depressed: 22%	Administration: Not stated<br>Language: English	DSM<br>C-DIS	a) COI declaration "None disclosed"<br>b) Funding acknowledged (academic/health research institutions)
Thompson et al. (2010)	Country: US	N = 214	Administration: Self administered	DSM-IV<br>SCID	a) No COI declaration<br>b) Funding acknowledged (academic/health research institutions)

For peer review only

Turner et al. (2012)	Setting: Patients with Parkinson Disease<br><br>Age (yrs): 72.5 (SD = 9.6)<br><br>Female: 42%<br><br>Country: Australia	Depressed: 14%<br><br>N = 72	Language: English<br><br>Administration: Self administered	DSM-IV	a) COI declaration: Disclosures 'None'.<br>b) Funding acknowledged (academic/health research institutions)
van Steenbergen-Weijenburg (2010)	Setting: Stroke patients<br><br>Age (yrs): 66.7 (SD = 13.1)<br><br>Female: 47.2%<br><br>Country: Netherlands	Depressed: 18%<br><br>N = 197	Language: English<br><br>Administration: Self administered	SCID<br><br>DSM-IV	a) COI declaration: 'The authors declare that they have no competing interests'.<br>b) Funding acknowledged (academic/health research institutions) – 'this had no influence on the content of this article'.

For peer review only - http://bmjopen.bmj.com/site/about/guidelines.xhtml

	Setting: Diabetes patients<br>Age (yrs): M = 61.8 (SD = 13.6)<br>Female: 48.7%	Depressed: 18.8%	Language: Dutch	SCID	
	Country: Netherlands	N = 1338	Administration: Self-report	DSM-IV	a) COI declaration 'The authors declare that they have no competing interests.'<br>b) Funding acknowledged (academic/health research institutions).
Zuitthoff et al. (2010)	Setting: Primary care<br>Age (yrs): M = 51 (sd = 16.7)<br>Female: 63%	Depressed: 13%	Language: Dutch	CIDI	

For peer review only

Table 2: Descriptive characteristics of the summed items scoring method studies cut-off point 10 (Moriarty et al, 2015)

Study	Sample characteristics	Sample size and % MDD	PHQ-9 characteristics	Diagnostic standard	a) Conflict of interest (COI) declaration<br>b) Funding<br>c) Relationship with original developers
13. Gräfe et al. (2004)	Country: Germany<br><br>Setting: psychosomatic walk-in clinics and family practices<br><br>Mean age: 41.9 (SD = 13.8)<br><br>Female: 67.8%	N = 528<br><br>Depressed: 29.2% psychosomatic patients; 6.16% medical patients	Administration: self-report<br><br>Language: German<br><br>Cut-offs: 10 to 14	DSM-IV SCID	c) No COI declaration<br>d) Acknowledged funding from Pfizer<br>e) Not acknowledged
16. Kroenke et al. (2001)	Country: USA<br><br>Setting: Primary care<br><br>Mean age: 46 (SD=17)<br><br>Female: 66%	N = 580<br><br>7.1% MDD	Administration: Self-report<br><br>Language: English<br><br>Cut-offs: 9 to 15	DSM-IV SCID	a) No COI declaration<br>b) Acknowledged funding from Pfizer<br>c) N/A
22. Navinés et al.	Country: Spain	N = 500	Administration: Self-	DSM–IV	`a) All authors declared

For peer review only

(2012)	Setting: General hospital (patients with chronic HCV)<br><br>Mean age: 43.4 (SD = 10.2)<br><br>Female: 28.6%	6.4% MDD	report<br><br>Language: Spanish<br><br>Cut-offs: 10	SCID	that they had no COI.<br>b) Role of funding source declared<br>c) Not acknowledged
29. Thekkumpurath et al. (2010)	Country: UK<br><br>Setting: Hospital (cancer patients)<br><br>Mean age: 61<br><br>Female: 63%	N = 782<br><br>6.3% MDD (of the whole sample)	Administration: Not stated<br><br>Language: English<br><br>Cut-offs: 5 to 10	DSM-IV SCID	c) COI declaration: 'Supported by Cancer Research UK'<br>d) As in a)<br>e) Not acknowledged
33. Williams et al. (2005)	Country: USA<br><br>Setting: Secondary care (Post-stroke)<br><br>Mean age: Unclear<br><br>Female: Unclear	N = 316<br><br>33.5% MDD	Administration: Unclear<br><br>Language: English<br><br>Cut-offs: 10	DSM-IV SCID	c) No COI declaration<br>d) Funding acknowledged (academic institutions)<br>e) Not acknowledged

Study	Sample characteristics	Sample size and % MDD	PHQ-9 characteristics	Diagnostic standar	d) Conflict of interest declaration<br>e) Funding

For peer review only

				d	
1. Adewuya et al. (2006)	Country: Nigeria<br><br>Setting: community (students)<br><br>Mean age: 24.8 (15-40)<br><br>Female: 41.2%	N = 512<br><br>2.5% MDD	Administration: Self-report<br><br>Language: English<br><br>Cut-offs: 8 to 12	DSM-IV MINI	a) No COI declaration<br>b) No funding declaration
2. Arroll et al. (2010)	Country: New Zealand<br><br>Setting: Primary care<br><br>Mean age: 49 (17-99)<br><br>Female: 61%	N = 2642<br><br>6.2% MDD	Administration: Not stated<br><br>Language: English<br><br>Cut-offs: 8,10,12,15	DSM-IV SCID	a) No COI declaration<br>b) Funding acknowledged (academic /health research institutions)
3. Azah et al. (2005)	Country: Malaysia<br><br>Setting: Primary care<br><br>Mean age: 38.7 (18-79)<br><br>Female: 61.7%	N = 180<br><br>16.6% MDD	Administration: Self-report<br><br>Language: Malay<br><br>Cut-offs: 5 to 12	DSM-IV CIDI	b) No COI declaration<br>c) Funding acknowledged (academic /health research institutions)
4. Chagas et al. (2013)	Country: Brazil	N = 84	Administration: self-report	DSM-IV SCID	a) COI declaration “None declared”

For peer review only

	Setting: Secondary care<br><br>Mean age: Not stated<br><br>Female: 52.7%	25.5% MDD	Language: Brazilian<br><br>Cut-offs: 7 to 10		b) Funding acknowledged (academic/health research institutions)
6. de Lima Osorio et al. (2009)	Country: Brazil<br><br>Setting: Primary care<br><br>Mean age: Unclear<br><br>Female: 100%	N = 177<br><br>34% MDD	Administration: research assistants<br><br>Language: Brazilian Portuguese<br><br>Cut-offs: 10 to 15	DSM-IV SCID	a) No COI declaration<br>b) Funding acknowledged (academic institutions)
7. Elderon et al. (2011)	Country: USA<br><br>Setting: Secondary care<br><br>Mean age: Unclear<br><br>Female: 18%	N = 1022<br><br>18.3% MDD	Administration: self-report<br><br>Language: English<br><br>Cut-offs: 10	C-DIS	a) COI declaration – 'No disclosures'<br>b) Funding acknowledged (academic institutions and industry – AHA Pharmaceuticals Roundtable) – 'The funding organisations had no role in the design or conduct of the study, collection, management, analysis or interpretation of

For peer review only

					data; or preparation, review or approval of the manuscript.'
8. Fann et al. (2005)	Country: US<br><br>Setting: Trauma hospital (inpatients with traumatic brain injury)<br><br>Mean age: 42 (SD=17.9)<br><br>Female: 29.1%	N = 135<br><br>16.3% MDD	Administration: Telephone-administered<br><br>Language: English<br><br>Cut-offs: 10	DSM-IV SCID	b) No COI declaration<br>c) Funding acknowledged (academic institutions)
9. Fine et al. (2013)	Country: USA<br><br>Setting: Primary care (Ohio Army National Guard)<br><br>Mean age: 31 (17-60)<br><br>Female: 12%	N = 498<br><br>21.5% MDD	Administration: Telephone-administered<br><br>Language: English<br><br>Cut-offs: 10,15	DSM-IV SCID-I	a) COI – last author disclosed financial and consulting interests (Pfizer not one of them). All other authors declared that they have no COI.<br>b) Funding acknowledged – DoD Medical Research. ''The sponsor had no role in study design, data collection, analysis,

For peer review only - http://bmjopen.bmj.com/site/about/guidelines.xhtml

					interpretation of results, report writing or manuscript submission.
10. Gelaye et al. (2013)	Country: Ethiopia<br><br>Setting: General hospital<br><br>Mean age: 34.9 (SD=11.6)<br><br>Female: 63.1 %	N = 363<br><br>12.6% MDD	Administration: Researcher-administered<br><br>Language: Amharic<br><br>Cut-offs: 9 to 11	DSM-IV SCAN	c) No COI declaration<br>d) Funding acknowledged (academic /health research institutions)
11. Gilbody et al. (2007)	Country: UK<br><br>Setting: Primary care<br><br>Mean age: 42.5 (SD 13.6)<br><br>Female: 77%	N = 96<br><br>37.5 MDD	Administration: Not stated<br><br>Language: English<br><br>Cut-offs: 9 to 13	DSM-IV SCID	a) COI declaration – last author involved in the development of one of the instruments (CORE-OM), 'but does not gain financially from its use.<br>b) Funding acknowledged (academic /health research institutions)
12. Gjerdingen et	Country: USA	N = 438	Administration:	DSM-IV	c) No COI declaration

For peer review only

al. (2009)	Setting: Community<br><br>Mean age: 29.3<br><br>Female: 100%	4.6% MDD	Telephone or self-report<br><br>Language: English<br><br>Cut-offs: 10	SCID	d) Funding acknowledged (academic /health research institutions)
14. Hyphantis et al. (2011)	Country: Greece<br><br>Setting: Hospital – rheumatology patients<br><br>Mean age: 54.2 (SD = 13.5)<br><br>Female: 74%	N = 213<br><br>32.4% MDD	Administration: Researcher administered<br><br>Language: Greek<br><br>Cut-offs: 4 to 16	DSM-IV MINI	c) No COI declaration<br>d) No funding acknowledgement
15. Khamseh et al. (2011)	Country: Iran<br><br>Setting: Outpatient diabetic clinic<br><br>Mean age: 56.1 (SD=9.6)<br><br>Female: 51.8%	N = 185<br><br>43.2% MDD	Administration: Self-report<br><br>Language: Persian<br><br>Cut-offs: 10,13	DSM-IV SCID	c) COI declaration: The authors declared no competing interests<br>d) Funding acknowledged (academic /health research institutions)

For peer review only

19. Liu et al. (2011)	Country: Taiwan<br><br>Setting: Primary care<br><br>Mean age: Not specified<br><br>Female: 60.9%	N = 1532<br><br>3.3% MDD	Administration: Self-report<br><br>Language: Chinese version<br><br>Cut-offs: 9 to 11	SCAN	a) a) No COI declaration<br>b) Funding acknowledged (academic /health research institutions)
20. Lotrakul et al. (2008)	Country: Thailand<br><br>Setting: Primary care<br><br>Mean age: 45.0 (SD = 14.30)<br><br>Female: 73.7%	N = 279<br><br>6.8% MDD	Administration: Self report<br><br>Language: Thai<br><br>Cut-offs: 7 to 15	DSM-IV MINI	c) No COI declaration<br>d) Funding acknowledged (academic /health research institutions)
23. Patel et al. (2008)	Country: India<br><br>Setting: Primary care<br><br>Mean age: 37.5 (18-83)<br><br>Female: 56.4%	N = 299<br><br>4.3% MDD	Administration: Face-to-face interview<br><br>Language: Not specified<br><br>Cut-offs: 7 to 15	CIS-R	a) COI declaration – No Declaration of Interest<br>b) Funding acknowledged (academic /health research institutions)
24. Phelan et al. (2010)	Country: USA<br><br>Setting: Primary care	N = 71<br><br>12% MDD	Administration: Research assistant	DSM-IV SCID	a) COI declaration – No competing interests

For peer review only

	(elderly)<br><br>Mean age: 78 (SD=7)<br><br>Female: 62%		Language: English<br><br>Cut-offs: 8 to 12		b) Funding acknowledged (academic /health research institutions) . 'The funder had no role in the study design, methods, data collection, analysis or interpretation of data, nor any role in the preparation of the manuscript or decision to submit the manuscript for publication.
25. Rooney et al. (2013)	Country: UK<br><br>Setting: Secondary care (glioma)<br><br>Mean age: 54.2 (SD=12.3)<br><br>Female: 42.6%	N = 129<br><br>13.5% MDD	Administration: Self-report<br><br>Language: English<br><br>Cut-offs: 8 to 11	DSM-IV SCID	a) COI declaration "The authors declare that they have no COI"<br>b) Funding acknowledged (academic/health research institutions)
26. Sherina et al. (2012)	Country: Malaysia<br><br>Setting: Primary care	N= 146<br><br>21.2% MDD	Administration: Self-report	CIDI	a) COI declaration "The authors declare that they

For peer review only

	Mean age: 30.9 (18-81)<br><br>Female: 100%		Language: Malay<br><br>Cut-offs: 10		have no competing interests"<br>b) Funding acknowledged (academic/health research institutions)
27. Sidebottom et al. (2012)	Country: USA<br><br>Setting: Community (prenatal)<br><br>Mean age: 23 (SD=5.5)<br><br>Female: 100%	N = 745<br><br>3.6% MDD	Administration: Interview<br><br>Language: English<br><br>Cut-offs: 10	DSM-IV SCID	a) COI declaration "The authors declare that they have no financial COI"<br>b) Funding acknowledged (academic/health research institutions)
28. Stafford et al. (2007)	Country: Australia<br><br>Setting: Secondary care (cardiac procedures)<br><br>Mean age: 64.14 (38-91)<br><br>Female: 19.2%	N = 193<br><br>18.1% MDD	Administration: Self-report<br><br>Language: English<br><br>Cut-offs: 10	DSM-IV MINI	b) No COI declaration<br>c) Funding acknowledged (academic/health research institutions)
30. Thombs et al. (2008)	Country: US<br><br>Setting: Hospital (outpatients with coronary heart disease)<br><br>Mean age: 67 (SD = 11)	N = 1024<br><br>22% MDD	Administration: Not stated<br><br>Language: English<br><br>Cut-offs: 7 to 10	DSM C-DIS	b) COI declaration "None disclosed"<br>b) Funding acknowledged (academic/health research institutions)

	Female: 18%				
32. Watnick et al. (2005)	Country: USA<br><br>Setting: Secondary care (dialysis)<br><br>Mean age: 63 (SD=15)<br><br>Female: 32.3%	N = 62<br><br>19% MDD	Administration: Self-report<br><br>Language: English<br><br>Cut-offs: 10	DSM-IV SCID	a) No COI declaration<br>b) Funding acknowledged (academic/health research institutions)
34. Wittkampf et al. (2009)	Country: Netherlands<br><br>Setting: Primary care<br><br>Mean age: 49.8<br><br>Female: 66.7%	N = 664<br><br>12.3% MDD	Administration: Self-report<br><br>Language: Not specified<br><br>Cut-offs: 10 and 15	DSM-IV SCIDI	a) No COI declaration<br>b) Funding acknowledged (academic/health research institutions)
35. Zhang et al. (2013)	Country: Hong Kong<br><br>Setting: Secondary care (diabetic outpatients)<br><br>Mean age: 55.1 (SD=9.5)<br><br>Female: 40.8%	N = 99<br><br>23.2% MDD	Administration: Self-report<br><br>Language: Chinese version<br><br>Cut-offs: 15	DSM-IV MINI	a) COI declaration – last author acknowledged financial COI. The other authors declare that they have no competing interests.<br>b)) Funding

					acknowledged (academic/health research institutions)
36. Zuithoff et al. (2010)	Country: Netherlands<br><br>Setting: Primary care<br><br>Age (yrs): M = 51 (sd = 16.7)<br><br>Female: 63%	N = 1338<br><br>Depressed: 13%	Administration: Self-report<br><br>Language: Dutch	DSM-IV CIDI	b) COI declaration "The authors declare that they have no competing interests.<br>b) Funding acknowledged (academic/health research institutions)

Table 3: Quality assessment of included studies in the algorithm meta-analysis (Manea et al., 2014)

For peer review only

**Study**	**Patient selection:** Consecutive or random sample	**Patient selection:** Avoid case-control / avoid artificially inflated base rate	**Patient selection:** Avoided inappropriate exclusions	**Patient selection:** Overall risk of bias	**Index test:** PHQ-9 interpreted blind to reference test	**Index test:** If translated, appropriate translation	**Index test:** If translated, psychometric properties reported	**Index test:** Overall risk of bias
Diez-Quevedo et al. (2001)	×	✓	×	High	?	✓	✓	Unclear
Gräfe et al. (2004)	✓	✓	✓	Low	?	✓	✓	Unclear
Lowe et al. (2004)	×	✓	✓	High	✓	✓	✓	Low
Muramatsu et al. (2007)	?	✓	?	Unclear	✓	✓	?	Unclear
Navines et al. (2012)	✓	✓	✓	Low	✓	✓	?	Unclear
Spitzer et al. (1999)	×	✓	✓	High	✓	n/a	n/a	Low
Thekkumpurath et al. (2010)	×	×	✓	High	✓	n/a	n/a	Low
Arroll et al. (2010)	✓	✓	✓	Low	✓	n/a	n/a	Low
Ayalon et al. (2010)	?	✓	✓	Unclear	?	✓	?	Unclear
Eack et al. (2006)	?	✓	?	Unclear	?	n/a	n/a	Unclear
Fann et al. (2005)	✓	×	×	High	✓	n/a	n/a	Low
Gelaye et al. (2013)	?	×	?	High	✓	✓	?	Unclear
Gjerdingen et al. (2009)	✓	✓	✓	Low	?	n/a	n/a	Unclear
Henkel et al. (2004)	✓	✓	✓	Low	?	n/a	n/a	Unclear
Hyphantis et al. (2011)	✓	✓	×	High	✓	?	?	Unclear

For peer review only

For peer review only - http://bmjopen.bmj.com/site/about/guidelines.xhtml

Inagaki et al. (2013)	✓	×	✓	High	✓	?	?	Unclear
Khamseh et al. (2011)	✓	✓	?	Unclear	✓	✓	?	Unclear
Lamers et al (2008)	✓	×	×	High	✓	?	?	Unclear
Lotrakul et al. (2008)	×	✓	?	High	✓	✓	?	Unclear
Persoons et al. (2003)	✓	✓	✓	Low	✓	✓	n/a	Low
Picardi et al. (2005)	✓	✓	✓	Low	✓	?	?	Unclear
Stafford et al. (2007)	✓	✓	✓	Low	✓	n/a	n/a	Low
Thombs et al. (2008)	×	✓	?	Unclear	?	n/a	n/a	Unclear
Thomspon et al. (2011)	?	✓	✓	Unclear	?	n/a	n/a	Unclear
Turner et al. (2012)	✓	✓	✓	Low	✓	n/a	n/a	Low
Van Steenbergen-Wijenburg (2010)	?	✓	✓	Unclear	?	?	?	Unclear
Zuithoff et al. (2010)	✓	✓	✓	Low	✓	✓	?	Unclear

✓ = criterion met; × = criterion not met; ? = insufficient information to code whether criterion met; n/a = not applicable

[1]If studies reported multiple cut-off points, 'threshold pre-specified' is coded as not applicable.

For peer review only

For peer review only - http://bmjopen.bmj.com/site/about/guidelines.xhtml

Table 3: Quality assessment of included studies in the algorithm meta-analysis (Manea et al., 2015) (continued)

Study	Reference test: Reference test correctly classifies target condition	Reference test: Reference test interpreted blind to PHQ-9	Reference test: If translated, appropriate translation	Reference test: If translated, psychometric properties reported	Reference test: Overall risk of bias	Flow / timing: Interval of two weeks or less	Flow / timing: All participants receive same reference test	Flow / timing: All participants included in analysis?	Flow / timing: Overall risk of bias
Diez-Quevedo et al. (2001)	✓	✓	✓	?	Unclear	✓	✓	✓	Low
Gräfe et al. (2004)	✓	?	n/a	n/a	Unclear	✓	✓	✓	Low
Lowe et al. (2004)	✓	✓	n/a	n/a	Low	✓	✓	✓	Low
Muramatsu et al. (2007)	✓	✓	✓	✓	Low	✓	✓	?	Unclear
Navines et al. (2012)	✓	✓	?	?	Unclear	✓	✓	✓	Low
Spitzer et al. (1999)	✓	✓	n/a	n/a	Low	✓	✓	×	High
Thekkumpurath et al. (2010)	✓	✓	n/a	n/a	Low	?	✓	×	High
Arroll et al. (2010)	✓	✓	n/a	n/a	Low	✓	✓	✓	Low
Ayalon et al. (2010)	✓	?	✓	?	Unclear	?	✓	✓	Unclear
Eack et al. (2006)	✓	?	n/a	n/a	Unclear	?	✓	?	Unclear
Fann et al. (2005)	✓	?	n/a	n/a	Unclear	✓	✓	×	High
Gelaye et al. (2013)	✓	✓	✓	✓	Low	✓	✓	×	High
Gjerdingen et al. (2009)	✓	?	n/a	n/a	Unclear	✓	✓	×	High
Henkel et al. (2004)	✓	?	n/a	n/a	Unclear	✓	✓	×	High

For peer review only

Table 4: Quality assessment of included studies in the summed item scoring method cut-off point 10 meta-analysis (Moriarty et al., 2015)

Hyphantis et al. (2011)	✓	✓	?	?	Unclear	✓	✓	×	High
Inagaki et el. (2013)	✓	✓	✓	?	Unclear	✓	✓	×	High
Khamseh et al (2011)	✓	✓	✓	?	Unclear	✓	✓	?	Unclear
Lamers et al. (2008)	✓	✓	?	?	Unclear	?	✓	×	High
Lotrakul et al. (2008)	✓	✓	✓	✓	Low	?	✓	×	High
Persoons et al. (2003)	✓	✓	?	?	Unclear	✓	✓	✓	Low
Picardi et al. (2005)	✓	✓	✓	?	Unclear	✓	✓	×	High
Stafford et al. (2007)	✓	✓	n/a	n/a	Low	✓	✓	×	High
Thombs et al. (2008)	?	✓	n/a	n/a	Unclear	✓	✓	✓	Low
Thompson et al. (2011)	✓	?	n/a	n/a	Unclear	✓	✓	×	High
Turner et al. (2012)	✓	?	n/a	n/a	Unclear	?	✓	×	High
Van Steenbergen-Wijenburg (2010)	✓	×	?	?	High	✓	✓	×	High
Zuithoff et al. (2010)	✓	✓	?	?	Unclear	?	✓	✓	Unclear

✓ = criterion met; × = criterion not met; ? = insufficient information to code whether criterion met; n/a = not applicable

For peer review only

Study	Patient selection: Consecutive or random sample	Patient selection: Avoid case-control / avoid artificially inflated base rate	Patient selection: Avoided inappropriate exclusions	Patient selection: Overall risk of bias	Index test: PHQ-9 interpreted blind to reference test	Index test: Was a threshold pre-sepecifed?	Index test: If translated, appropriate translation	Index test: If translated, psychometric properties reported	Index test: Overall risk of bias
13. Gräfe et al. (2004)	✓	✓	✓	Low	?	✓	✓	✓	Unclear
16. Kroenke et al. (2011)	✓	✓	✓	Low	✓	✓	n/a	n/a	Low
22. Navinés et al. (2012)	✓	✓	✓	Low	✓	✓	✓	?	Unclear
29. Thekkumpurath et al. (2010)	✗	✗	✓	High	✓	✓	n/a	n/a	Low
33. Williams et al. (2005)	✓	✓	✓	Low	?	✓	n/a	n/a	Unclear
1. Adewuya et al. (2006)	✓	✓	✗	Unclear	✓	✓	n/a	n/a	Low
2. Arroll et al. (2010)	✓	✓	✓	Low	✓	✓	n/a	n/a	Low
3. Azah et al. (2005)	✓	✗	?	High	✓	✓	✓	✓	Low
4. Chagas et al. (2013)	✓	✓	✓	Low	✓	✓	✓	✓	Low
6. de Lima Osorio et al. (2009)	✓	✗	✓	High	?	✗	n/a	n/a	High
7. Elderon et al.	✓	✓	✓	Low	✓	✓	n/a	n/a	Low

For peer review only

(2011)									
8. Fann et al. (2005)	✓	✗	✗	High	✓	✓	n/a	n/a	Low
9. Fine et al. (2013)	✓	✓	✓	Low	?	✓	n/a	n/a	Unclear
10. Gelaye et al. (2013)	?	✗	?	High	✓	✗	✓	?	High
11. Gilbody et al. (2007)	?	✓	?	Unclear	✓	✓	n/a	n/a	Low
12. Gjerdingen et al. (2009)	✓	✓	✓	Low	?	✓	n/a	n/a	Unclear
14. Hyphantis et al. (2011)	✓	✗	✓	High	✓	✓	?	?	Unclear
15. Khamseh et al. (2011)	✓	✓	?	Unclear	✓	✓	✓	?	Unclear
19. Liu et al. (2011)	✓	✓	?	Unclear	✓	✗	✓	?	High
20. Lotrakul et al. (2008)	✗	✓	?	Unclear	✓	✓	✓	?	Unclear
23. Patel et al. (2008)	✓	✓	✓	Low	✓	✓	?	?	Unclear
24. Phelan et al. (2010)	✗	✓	✓	High	✓	✗	n/a	n/a	High
25. Rooney et al. (2013)	✓	✓	✓	Low	?	✗	n/a	n/a	High
26. Sherina et al. (2012)	✓	✓	✗	High	✓	✓	✓	✓	Low
27. Sidebottom	✓	✓	✓	Low	✓	✓	n/a	n/a	Low

For peer review only

et al. (2012)									
28. Stafford et al. (2007)	✓	✓	✓	Low	✓	✓	n/a	n/a	Low
30. Thombs et al. (2008)	✗	✓	?	High	✓	?	n/a	n/a	Unclear
32. Watnick et al. (2005)	?	✗	✓	High	✓	✓	n/a	n/a	Low
34. Wittkampf et al. (2009)	✓	✓	✓	Low	✓	?	n/a	n/a	Unclear
35. Zhang et al. (2013)	✓	✓	?	Unclear	?	✓	?	?	Unclear
36. Zuithoff et al. (2010)	✓	✓	✓	Low	✓	✓	✓	?	Unclear

Table 4: Quality assessment of included studies in the summed item scoring method cut-off point 10 meta-analysis (Moriarty et al., 2015)

Study	Reference test: Reference test correctly classifies target condition	Reference test: Reference test interpreted blind to PHQ-9	Reference test: If translated, appropriate translation	Reference test: If translated, psychometric properties reported	Reference test: Overall risk of bias	Flow / timing: Interval of two weeks or less	Flow / timing: All participants receive same reference test	Flow / timing: All participants included in analysis?	Flow / timing: Overall risk of bias
13. Gräfe et al. (2004)	✓	?	n/a	n/a	Unclear	✓	✓	✓	Low
16. Kroenke et	✓	✓	n/a	n/a	Low	✓	✓	✓	Low

For peer review only

al. (2011)									
22. Navinés et al. (2012)	✓	✓	?	?	Unclear	✓	✓	✓	Low
29. Thekkumpurath et al. (2010)	✓	✓	n/a	n/a	Low	?	✓	✓	Unclear
33. Williams et al. (2005)	✓	?	n/a	n/a	Unclear	?	✓	✓	Unclear
1. Adewuya et al. (2006)	✓	✓	n/a	n/a	Low	✓	✓	✓	Low
2. Arroll et al. (2010)	✓	✓	n/a	n/a	Low	?	✓	✓	Unclear
3. Azah et al. (2005)	✓	✓	✓	✓	Low	✓	✓	✗	High
4. Chagas et al. (2013)	✓	✓	?	?	Unclear	✓	✓	✗	High
6. de Lima Osorio et al. (2009)	✓	?	n/a	n/a	Unclear	?	✓	✓	Unclear
7. Elderon et al. (2011)	✓	✓	n/a	n/a	Low	✓	✓	✓	Low
8. Fann et al. (2005)	✓	?	n/a	n/a	Unclear	✓	✓	✗	High
9. Fine et al. (2013)	✓	?	n/a	n/a	Unclear	?	✓	✓	Unclear
10. Gelaye et al. (2013)	✓	✓	✓	✓	Low	✓	✓	✗	High

For peer review only

11. Gilbody et al. (2007)	✓	✓	n/a	n/a	Low	?	✓	✓	Unclear
12. Gjerdingen et al. (2009)	✓	?	n/a	n/a	Unclear	✓	✓	✗	High
14. Hyphantis et al. (2011)	✓	✓	?	?	Unclear	✓	✓	✗	High
15. Khamseh et al. (2011)	✓	✓	✓	?	Unclear	✓	✓	?	Unclear
19. Liu et al. (2011)	✓	✓	✓	✓	Low	✓	✓	?	Unclear
20. Lotrakul et al. (2008)	✓	✓	✓	✓	Low	?	✓	✗	High
23. Patel et al. (2008)	✓	✓	✓	?	Unclear	?	✓	✗	High
24. Phelan et al. (2010)	✓	✓	n/a	n/a	Low	✓	✓	✓	Low
25. Rooney et al. (2013)	✓	?	n/a	n/a	Unclear	?	✓	✗	High
26. Sherina et al. (2012)	✓	✓	✓	✓	Low	✓	✓	✓	Low
27. Sidebottom et al. (2012)	✓	✓	n/a	n/a	Low	✓	✓	✗	High
28. Stafford et al. (2007)	✓	✓	n/a	n/a	Low	✓	✓	✗	High
30. Thombs et al. (2008)	?	✓	n/a	n/a	Unclear	✓	✓	✓	Low
32. Watnick et al. (2005)	✓	✓	n/a	n/a	Low	✓	✓	✓	Low
34. Wittkampf et al. (2009)	✓	✓	n/a	n/a	Low	?	✓	✗	High

For peer review only

35. Zhang et al. (2013)	✓	?	✓	✓	Unclear	✗	✓	✗	High
36. Zuithoff et al. (2010)	✓	✓	?	?	Unclear	?	✓	✓	Unclear

Table 5. Pooled estimates of diagnostic properties of the PHQ-9 at cut-off point 10 and using algorithm scoring method in the non-independent vs independent studies groups

Settings	No of studies	No of patients	Sensitivity (95% CI)	Specificity (95% CI)	Pooled positive likelihood ratio (95% CI)	Pooled negative likelihood ratio (95% CI)	Diagnostic odds ratio (95% CI)	Heterogeneity: I^2
**Manea et al, 2014 SR – RA group**	7	4,065	0.77 (0.70 – 0.84)	0.94 (0.90 – 0.97)	14.97 (8.39 – 26.71)	0.23 (0.17 - 0.31)	64.40 (34.15 – 121.43)	78.9%
**Manea et al, 2014 SR Independent studies**	21	9,900	0.48 (0.41 – 0.91)	0.94 (0.91 – 0.95)	8.26 (6.15 – 11.09)	0.54 (0.48 – 0.62)	15.05 (11.03 – 20.52)	68.1%
**Moriarty et al., 2015 SR – RA group**	5	6,188	0.87 (0.77 – 0.93)	0.87 (0.76 – 0.94)	7.24 (3.74 – 14.03)	0.14 (0.08 - 0.25)	49.31 (25.74 – 94.48)	55.1%
**Moriarty et al., 2015 SR Independent studies**	26	13,164	0.76 (0.67 – 0.83)	0.88 (0.85 – 0.91)	6.72 (5.06 – 8.92)	0.26 (0.19 - 0.37)	24.96 (14.81 – 42.08)	81.5%

For peer review only - http://bmjopen.bmj.com/site/about/guidelines.xhtml

For peer review only

Appendix 1

Figure 1: PRISMA flowchart - search and selection of included diagnostic accuracy studies for the systematic review of studies reporting diagnostic accuracy of the PHQ-9 at using the summed items scoring method (Manea et al, 2014)

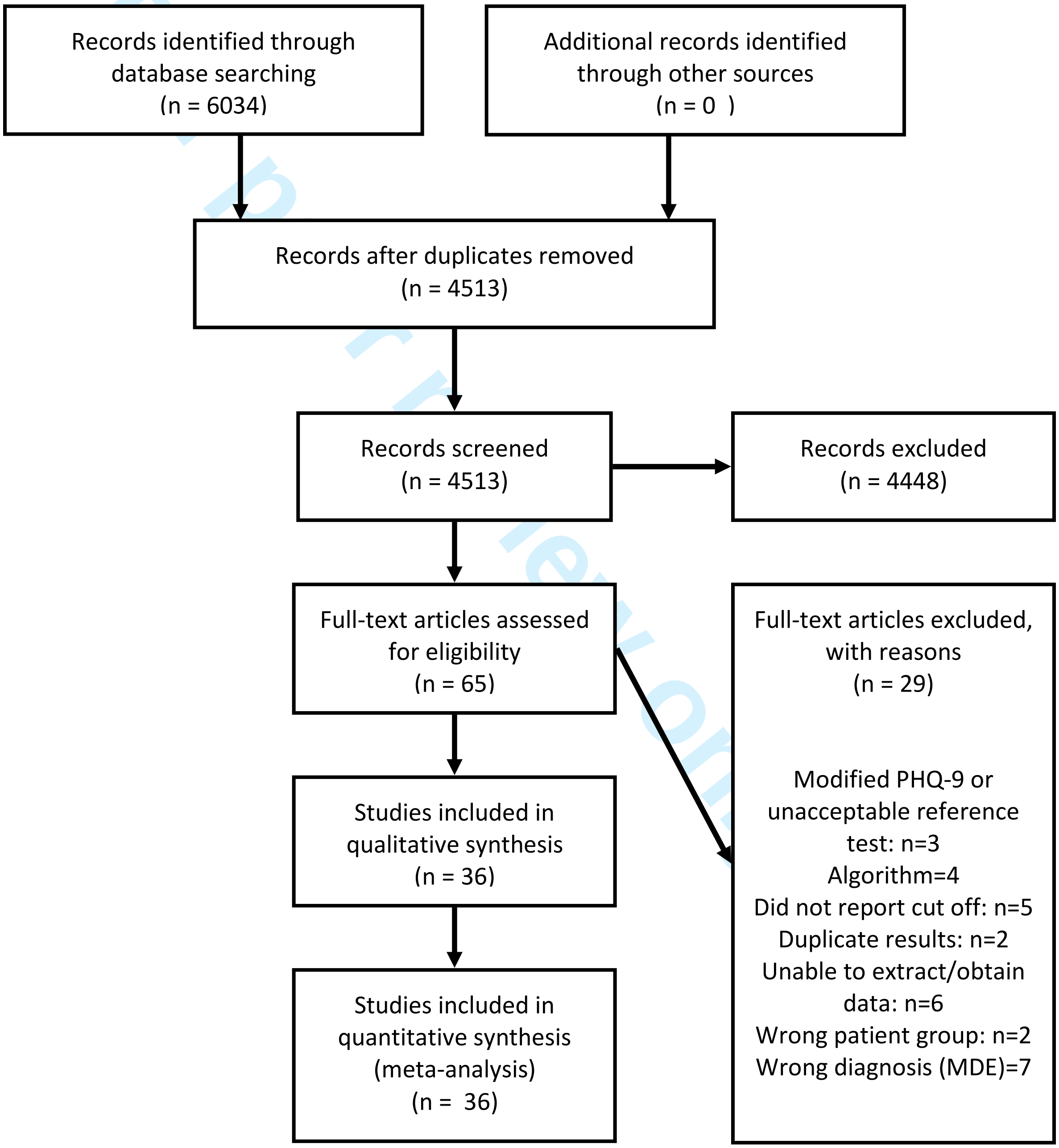

Figure 2: PRISMA flowchart - search and selection of included diagnostic accuracy studies for the systematic review of studies reporting diagnostic accuracy of the PHQ-9 at using the algorithm scoring method (Moriarty et al., 2015)

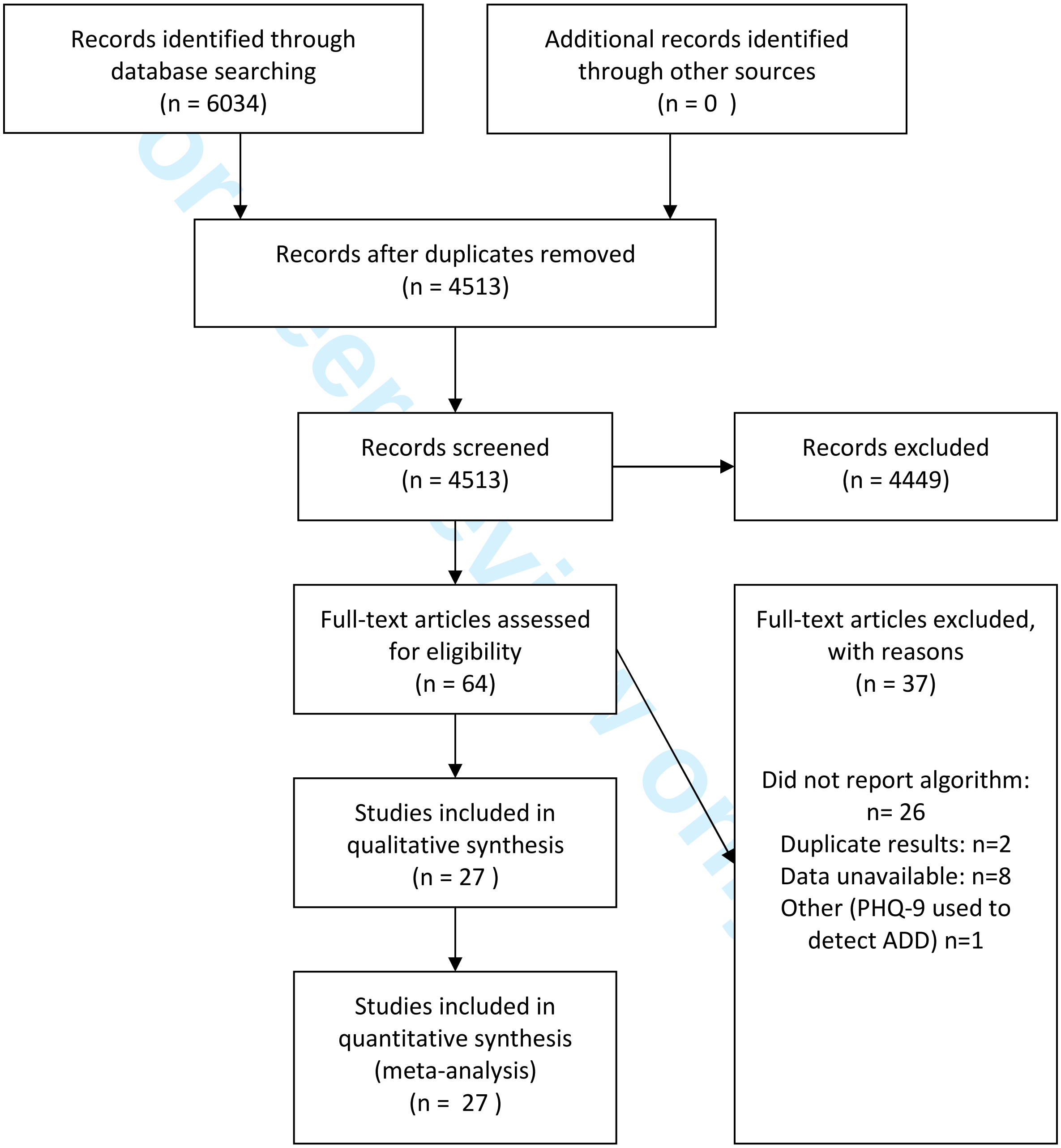

For peer review only - http://bmjopen.bmj.com/site/about/guidelines.xhtml

PRISMA 2009 Checklist

Section/topic	#	Checklist item	Reported on page #
**TITLE**			
Title	1	Identify the report as a systematic review, meta-analysis, or both.	1
**ABSTRACT**			
Structured summary	2	Provide a structured summary including, as applicable: background; objectives; data sources; study eligibility criteria, participants, and interventions; study appraisal and synthesis methods; results; limitations; conclusions and implications of key findings; systematic review registration number.	2
**INTRODUCTION**			
Rationale	3	Describe the rationale for the review in the context of what is already known.	4-5
Objectives	4	Provide an explicit statement of questions being addressed with reference to participants, interventions, comparisons, outcomes, and study design (PICOS).	5
**METHODS**			
Protocol and registration	5	Indicate if a review protocol exists, if and where it can be accessed (e.g., Web address), and, if available, provide registration information including registration number.	No
Eligibility criteria	6	Specify study characteristics (e.g., PICOS, length of follow-up) and report characteristics (e.g., years considered, language, publication status) used as criteria for eligibility, giving rationale.	5
Information sources	7	Describe all information sources (e.g., databases with dates of coverage, contact with study authors to identify additional studies) in the search and date last searched.	5
Search	8	Present full electronic search strategy for at least one database, including any limits used, such that it could be repeated.	Available online (see Manea et al., 2015; Moriarty et al., 2015)
Study selection	9	State the process for selecting studies (i.e., screening, eligibility, included in systematic review, and, if applicable, included in the meta-analysis).	5
Data collection process	10	Describe method of data extraction from reports (e.g., piloted forms, independently, in duplicate) and any processes for obtaining and confirming data from investigators.	6
Data items	11	List and define all variables for which data were sought (e.g., PICOS, funding sources) and any assumptions and simplifications made.	5-6
Risk of bias in individual studies	12	Describe methods used for assessing risk of bias of individual studies (including specification of whether this was done at the study or outcome level), and how this information is to be used in any data synthesis.	6

For peer review only

PRISMA 2009 Checklist

Summary measures	13	State the principal summary measures (e.g., risk ratio, difference in means).	6
Synthesis of results	14	Describe the methods of handling data and combining results of studies, if done, including measures of consistency (e.g., I^2) for each meta-analysis.	6

Section/topic	#	Checklist item	Reported on page #
Risk of bias across studies	15	Specify any assessment of risk of bias that may affect the cumulative evidence (e.g., publication bias, selective reporting within studies).	6, 21
Additional analyses	16	Describe methods of additional analyses (e.g., sensitivity or subgroup analyses, meta-regression), if done, indicating which were pre-specified.	6
**RESULTS**			
Study selection	17	Give numbers of studies screened, assessed for eligibility, and included in the review, with reasons for exclusions at each stage, ideally with a flow diagram.	Appendix
Study characteristics	18	For each study, present characteristics for which data were extracted (e.g., study size, PICOS, follow-up period) and provide the citations.	Tables 1 and 2
Risk of bias within studies	19	Present data on risk of bias of each study and, if available, any outcome level assessment (see item 12).	Tables 3 and 4
Results of individual studies	20	For all outcomes considered (benefits or harms), present, for each study: (a) simple summary data for each intervention group (b) effect estimates and confidence intervals, ideally with a forest plot.	N/A
Synthesis of results	21	Present results of each meta-analysis done, including confidence intervals and measures of consistency.	Table 5
Risk of bias across studies	22	Present results of any assessment of risk of bias across studies (see Item 15).	Tables 3 and 4
Additional analysis	23	Give results of additional analyses, if done (e.g., sensitivity or subgroup analyses, meta-regression [see Item 16]).	11 and 17
**DISCUSSION**			
Summary of evidence	24	Summarize the main findings including the strength of evidence for each main outcome; consider their relevance to key groups (e.g., healthcare providers, users, and policy makers).	17-21
Limitations	25	Discuss limitations at study and outcome level (e.g., risk of bias), and at review-level (e.g., incomplete retrieval of identified research, reporting bias).	21
Conclusions	26	Provide a general interpretation of the results in the context of other evidence, and implications for future research.	21-22
**FUNDING**			

For peer review only

PRISMA 2009 Checklist

Funding	27	Describe sources of funding for the systematic review and other support (e.g., supply of data); role of funders for the systematic review.	23

From: Moher D, Liberati A, Tetzlaff J, Altman DG, The PRISMA Group (2009). Preferred Reporting Items for Systematic Reviews and Meta-Analyses: The PRISMA Statement. PLoS Med 6(6): e1000097. doi:10.1371/journal.pmed1000097

For more information, visit: **www.prisma-statement.org**.

Page 2 of 2

For peer review only

BMJ Open

Are there researcher allegiance effects in diagnostic validation studies of the PHQ-9? A systematic review and meta-analysis

Journal:	*BMJ Open*
Manuscript ID	bmjopen-2016-015247.R1
Article Type:	Research
Date Submitted by the Author:	08-May-2017
Complete List of Authors:	Manea, Laura; University of York, Health Sciences<br>Boehnke, Jan Rasmus; University of York<br>Gilbody, Simon; The University of York, Department of Health Sciences<br>Moriarty, Andrew; University of York, Health Sciences<br>McMillan, Dean; University of York, Department of Health Sciences
**Primary Subject Heading**:	Mental health
Secondary Subject Heading:	Diagnostics
Keywords:	Depression & mood disorders < PSYCHIATRY, Screening, PHQ-9, diagnostic meta-analysis, allegiance effect

SCHOLARONE™
Manuscripts

For peer review only

Are there researcher allegiance effects in diagnostic validation studies of the PHQ-9? A systematic review and meta-analysis

Laura Manea MMedSci MRCPsych*, Jan R. Boehnke PhD, Simon Gilbody DPhil FRCPsych FRSA, Andrew S. Moriarty MRes, Dean McMillan PhD

*Corresponding Author

Hull York Medical School and Department of Health Sciences, ARRC Building, University of York, YO10 5DD

Email: laura.manea@york.ac.uk

For peer review only

For peer review only - http://bmjopen.bmj.com/site/about/guidelines.xhtml

Abstract

Objectives To investigate whether an authorship effect is found that leads to better performance in studies conducted by the original developers of the PHQ-9 (allegiant studies).

Design Systematic review with random effects bivariate diagnostic meta-analysis. Search strategies included electronic databases, examination of reference lists, and forward citation searches.

Inclusion criteria Included studies provided sufficient data to calculate the diagnostic accuracy of the PHQ-9 against a gold standard diagnosis of major depression using the algorithm or the summed item scoring method at cut-off point 10.

Data extraction Descriptive information, methodological quality criteria, and 2×2 contingency tables.

Results

For peer review only - http://bmjopen.bmj.com/site/about/guidelines.xhtml

Seven allegiant and twenty independent studies reported the diagnostic performance of the PHQ-9 using the algorithm scoring method. Pooled diagnostic odds ratio (DOR) for the allegiant group was 64.40, and 15.05 for non-allegiant studies group. The allegiance status was a significant predictor of DOR variation ($p < 0.0001$).

Five allegiant studies and twenty-six non-allegiant studies reported the performance of the PHQ-9 at recommended cut-off point of 10. Pooled DOR for the allegiant group was 49.31, and 24.96 for the non-allegiant studies. The allegiance status was a significant predictor of DOR variation ($P = 0.015$).

Some potential alternative explanations for the observed authorship effect including differences in study characteristics and quality were found, though it is not clear how some of them account for the observed differences

Conclusions

Allegiant studies reported better performance of the PHQ-9. Allegiance status was predictive of variation in the DOR. Based on the observed differences between independent and non-independent studies we were unable to conclude or exclude that allegiance effects are present in studies examining the diagnostic performance of the PHQ-9. This study highlights the need for future meta-analyses of diagnostic validation studies of psychological measures to evaluate the impact of researcher allegiance in the primary studies.

Strengths and limitations of this study

For peer review only - http://bmjopen.bmj.com/site/about/guidelines.xhtml

a) An original study–the first meta-analysis of diagnostic validation studies of psychological measures to evaluate the impact of researcher allegiance.
b) Using rigorous methodology–strict inclusion/exclusion and quality assessment criteria.
c) We found that the allegiance effect was a significant predictor of the variation of the diagnostic odds ratio in the meta-regression analysis.
d) Substantial variability observed in methodological quality of included studies.
e) Based on the observed methodological differences between the independent and non-independent studies we were unable to conclude or exclude that allegiance effects are present in studies examining the diagnostic performance of the PHQ-9.

For peer review only - http://bmjopen.bmj.com/site/about/guidelines.xhtml

Research on allegiance effects has a long tradition in psychotherapy research. In this context *allegiance* describes the phenomenon that researchers and clinicians who developed a treatment approach or are for other reasons invested in it tend to find larger effect sizes in favour of their treatment than for comparison groups. [1] This finding has been extensively replicated [2], [3] and is also robust when the quality of research is controlled for. Researcher allegiance is subject of on-going debates about the design of efficacy studies as well as implications for policy. [2], [4], [5] Researcher allegiance is also discussed widely in the literature on experimental as well as evaluation research. [6] Since the motivational underpinnings of allegiance effects are potentially far more ingrained into human behaviour and decision making than previously thought (e.g., [7], they may occur commonly in clinical research in general.

Although it has been suggested that allegiance effects may play a role in the validation of psychological screening and case-finding tools (e.g., O'Shea et al., in press), systematic evaluations of this hypothesis are rare and studies that acknowledge potential allegiance effects in such studies mainly come from forensic psychology and psychiatry backgrounds. [8]–[11] Diagnostic validation studies are geared at establishing the sensitivity and specificity of a screening or case finding tool, which is used in practice to differentiate cases from non-cases or to decide about whether further assessment or treatment is indicated or will be offered. An allegiance effect in such studies would be seen in systematically higher sensitivities or specificities if the original author(s) is (are) part of the team of such a study. Such a bias would have a deleterious affect on practice through promising over-optimistic accuracy of the screening or case finding tool or in evaluating the cost-effectiveness of the measure in a screening or case-finding context.

The depression module of the Patient Health Questionnaire (PHQ-9) is a widely used depression-screening instrument in non-psychiatric settings. The PHQ-9 was developed by a team of researchers, with its development underwritten by an educational grant from Pfizer US Pharmaceuticals. [12] The PHQ-9 can be scored using different methods, including an algorithm based on DSM-IV criteria and a cut-off based on summed-item scores. The psychometric properties of these two approaches have been summarised in two recently published meta-analyses. [13], [14] The goal of the current review is to investigate, based on an established database of PHQ-9 diagnostic validation studies [13], [14],

For peer review only - http://bmjopen.bmj.com/site/about/guidelines.xhtml

whether an allegiance effect is found that leads to an increased sensitivity and specificity in studies that were conducted by researchers closely connected to the original developers of the instrument.

METHODS

Study Selection

Similar search strategies were used in both systematic reviews. (For full details please see Manea et al. (2014) and Moriarty et al. (2015)). Embase, MEDLine and PSYCHInfo were searched from 1999 (when the PHQ-9 was first developed) to August 2013 [13] and September 2013 [14] respectively, using the terms "PHQ-9", "PHQ", "PHQ$" and "patient health questionnaire". The search strategy is presented in Appendix 2. The reference lists of studies fitting the inclusion criteria were manually searched and a reverse citation search in Web of Science was performed. Authors of unpublished studies were contacted and conference abstracts were reviewed in an attempt to minimise publication bias.

The following inclusion-exclusion criteria were used:

Population: Adult population. *Instrument:* Studies that used the PHQ-9. *Comparison (reference standard):* The accuracy of the PHQ-9 had to be assessed against a recognised gold-standard instrument for the diagnosis of either Diagnostic and Statistical Manual (DSM) or International Classification of Disease (ICD) criteria for major depression. Studies were included if the diagnoses were made using a standardised diagnostic structured interview schedule (e.g. Mini International Neuropsychiatric Interview (MINI), Structured Clinical Interview for DSM Disorders (SCID)). Unguided clinician diagnoses with no reference to a standard structured diagnostic schedule or comparisons of the PHQ-9 with other self-report measures were excluded. Studies were also excluded if the target diagnosis was not major depressive disorder (MDD, e.g. any depressive disorder). *Outcome:* Studies had to report sufficient information to calculate a 2*2 contingency table for the algorithm or the recommended cut-off point 10. *Study design:* Any design. *Additional criterion:* We avoided double counting of evidence by ensuring that only

For peer review only - http://bmjopen.bmj.com/site/about/guidelines.xhtml

one study of those that reported overlapping datasets in different journals were included in the meta-analysis. Citations with overlapping samples were examined to establish whether they contained information relevant to the research question that was not contained in the included report.

Quality assessment

Quality assessment was performed using the QUADAS-2 tool, a tool for evaluating the risk of bias and applicability of primary diagnostic accuracy studies when conducting diagnostic systematic reviews. [15] It covers the areas of: patient selection, index test, reference standard and flow and timing. [16] This tool was adapted for the two reviews and quality assessments were carried out by two independent reviewers for all studies included in the reviews.

Data synthesis and statistical analysis

We constructed 2x2 tables for cut-off point 10 [14] and the algorithm scoring method [13] Pooled estimates of sensitivity, specificity, positive/negative likelihood ratios, and diagnostic odds ratios were calculated using random effects bivariate meta-analysis. [17] Heterogeneity was assessed using I^2 for the diagnostic odds ratio, an estimate of the proportion of study variability that is due to between-study variability rather than sampling error. We considered values of ≥50% to indicate substantial heterogeneity.[18] Summary Receiver Operator Characteristic curves (sROC) were constructed using the bivariate model to produce a 95% confidence ellipse within ROC space. [19] Each data point in the summary ROC space represents a separate study, unlike a traditional ROC plot, which explores the effect varying thresholds on sensitivity and specificity in a single study.

We undertook a meta-regression analysis of logit diagnostic odds ratio using research allegiance as covariate in the meta-regression model. [20], [21] Analyses were conducted using STATA version 12, with the metan, metandi and metareg user-written commands.

Allegiance Rating

For peer review only - http://bmjopen.bmj.com/site/about/guidelines.xhtml

We rated authorship on a paper if any of the developers of the PHQ-9 - Kurt Kroenke, MD, Robert L Spitzer, MD, and Janet B W Williams – as an indicator of potential allegiance. We also rated as evidence of allegiance as acknowledged collaborations with the developers of the PHQ-9, even if they were not listed as co-authors or if the authors acknowledged funding from Pfizer to conduct the study.

RESULTS

Overview of included studies

31 studies reported the diagnostic properties of the PHQ-9 at cut-off point 10 or above and were included in this analysis. [14] 27 studies were included in the algorithm review [13]. The study selection flowcharts can be found in Appendix 1 (figures 1 and 2). The characteristics of these studies are reported in tables 1 and 2 and the results of the methodological assessment are presented in tables 3 and 4.

Algorithm scoring method

Descriptive characteristics

The descriptive characteristics of the included studies are presented in table 1. Seven individual studies that reported the diagnostic performance of the PHQ-9 using the algorithm scoring method were co-authored by the original developers of the PHQ-9 [22]–[26], specifically acknowledged one of the developers and support by an educational grant from Pfizer US [27], or were co-authored by the first author of a

For peer review only

For peer review only - http://bmjopen.bmj.com/site/about/guidelines.xhtml

previous study that had also been co-authored by one of the developers [28]. Twenty non-allegiant studies reported the diagnostic properties of the PHQ-9 using the algorithm scoring method.

Three (43%, 3/7) of the allegiant studies were conducted exclusively in hospital settings [22], [26], [28]. The remaining four studies (67%, 4/7) were conducted in different settings or non-exclusively hospital settings: one in primary care [25] and three in mixed settings: psycho-somatic walk in clinics and family practices [23][1], outpatient clinics and family practices [24] and primary care and hospital settings [27]. In the non-allegiant group, thirteen (65%, 13/20) studies were conducted in hospital settings [29]–[41]. Of the remaining seven studies, six were conducted in primary care settings [42]–[47] and one in a community sample [48].

In both groups (non-allegiant and allegiant studies), the majority of studies validated a translated version of the PHQ-9. Two of the studies authored by developers (28%, 2/7) [25], [26], and eight (40%, 8/20) allegiant studies [29], [30], [37]–[40], [42], [48] were conducted in English.

The mean prevalence of major depressive disorder in the group of allegiant studies was 13.4 % (range 6.1% - 29.2%); in the non-allegiant group it was 15.5% (range 3.9% - 32.4%). The mean age of patients in the PHQ-9 developers group was 45.7; all but one study had a mean age in the range of 40 to 50 years. In the non-allegiant group the mean age was 54.6 (range 29.3 – 75.0), with almost half (8) of the studies reporting a mean age of over 60. The percentage of females in the PHQ-9 developers was 56.8% (range 28.6% - 67.8%) and in the non-allegiant group was 59.1 (18% -100%).

[1] This study provided separate estimates for the two settings in which it was conducted; therefore separate psychometric estimates were generated for each sample for both algorithm scoring method and summed items scoring method at cut-off point 10 (see below).

For peer review only - http://bmjopen.bmj.com/site/about/guidelines.xhtml

All allegiant studies used a self-reported PHQ-9, whereas in 7 non-allegiant studies (30%, 6/20) the PHQ-9 was administered by a researcher [30]–[33], [43], [48]. Apart from Muramatsu et al. (2007) all allegiant studies used the SCID as a gold standard; the non-allegiant studies used a wider range of gold standards including SCAN, CIDI, MINI, and C-DIS, though the SCID was also frequently used by the independent studies as well (45%, 9/20 studies).

Four out of the seven allegiant studies (57%) did not include a conflict of interests statement [22], [23], [25], [27]. Also, four (57%) of the allegiant studies acknowledged funding from Pfizer [23]–[25], [27]. Only one study [27] acknowledged the collaboration with one of the developers of the PHQ-9.

Of the non-allegiant studies, twelve (60%) did not include a conflict of interests statement [29]–[32], [35]–[37], [39], [45], [46], [48], [49]. It appears that newer studies were more likely to include a conflict of interest statement, which may reflect a recent change in reporting. Funding was acknowledged by most studies (18/20) and most received funding from academic or/and health research institutions. Two studies received funding from pharmaceutical companies – Lundbeck [43] and Pfizer [35] and one study acknowledged that Pfizer Italia provided the Italian version of PHQ-9 and gave the authors permission to use it [36].

Diagnostic test accuracy

Pooled sensitivity and specificity was calculated separately for the non-allegiant and allegiant studies. Pooled sensitivity for the allegiant studies of the PHQ-9 was 0.77 (95% CI = 0.70 – 0.84), pooled specificity was 0.94 (95% CI = 0.90 – 0.97), and the pooled diagnostic odds ratio was 64.40 (95% CI = 34.15 – 121.43). Heterogeneity was high (I^2 = 78.9%). Figure 1 represents the summary ROCs for this set of studies.

For peer review only - http://bmjopen.bmj.com/site/about/guidelines.xhtml

----

Figure 1. PHQ-9 algorithm scoring method summary ROC plot for the diagnosis of major depressive disorder in allegiant studies (Panel A) and non-allegiant studies (Panel B). Pooled sensitivity and specificity estimates using a bi-variate meta-analysis (*HSROC* hierarchical receiver-operating characteristic).

Pooled sensitivity for the non-allegiant studies was lower compared to the developer authored studies group at 0.48 (95% CI = 0.41 – 0.91), pooled specificity was the same at 0.94 (95% CI = 0.91 – 0.95). The pooled diagnostic odds ratio was approximately four times lower at 15.05 (95% CI = 11.03 – 20.52) (see figure 1). Heterogeneity was substantial at $I^2 = 68.1$ %.

The meta-regression analysis for algorithm studies with non-allegiant status as the predictor of the diagnostic odds ratio showed that non-allegiant status was a significant predictor of the diagnostic odds ratio ($p < 0.0001$) and explained a substantial amount of the observed heterogeneity (51.5%).

<u>*Quality assessment*</u>

For peer review only

For peer review only - http://bmjopen.bmj.com/site/about/guidelines.xhtml

The results of the quality assessment using QUADAS-2 are given in table 3 for the studies reporting on the diagnostic performance of the algorithm scoring method. In the patient selection domain, more non-allegiant studies (65%, 13/20) than allegiant (29%, 2/7) met the criterion for consecutive referrals. There were no marked differences on the other two criteria in this domain (avoid case-control design, avoid inappropriate exclusions). In the index test domain, the proportion of studies reporting that the PHQ-9 was conducted blind to the reference test was comparable between the two groups. There were differences in this domain for those studies using a translated version of the test. All non-English allegiant studies (5/5) used an appropriately translated version of the PHQ-9; whereas just over a half of the non-allegiant studies reported this (55%, 6/11). However, the majority of both sets of studies did not report details of psychometric properties of the translated version. For the reference test domain, nearly all studies in both groups were rated as using a reference test that would correctly classify the condition. While most allegiant studies reported that the reference test was interpreted blind to the PHQ-9 score (86%, 6/7), this was reported in only 60% (12/20) of the non-allegiant studies.

The two sets of studies that used translated versions of the reference test were broadly comparable. There was a slight indication that the allegiant studies were more likely to use an appropriately translated version of the reference test and report data on the psychometric properties of the translated version, though the numbers for the translated comparison are very low. There were, however, some more notable differences on the flow and timing domain. Most allegiant studies ensured that the time between the index and reference test was under two weeks (86%, 6/7) in comparison to 70% (14/20) of the non-allegiant studies. More allegiant studies met the criterion for 'all participants included in the analysis' (57%, 4/7) than non-allegiant studies (25%).

Summed items scoring method (cut-off point 10 or above)

For peer review only - http://bmjopen.bmj.com/site/about/guidelines.xhtml

Descriptive characteristics

Table 2 presents the sample characteristics of the thirty-one PHQ-9 validation studies that reported the psychometric properties of the PHQ-9 at cut-off point 10 or above. Five of these studies were co-authored by the original developers of the instrument or acknowledged collaboration [12], [23], [26], [50] or were co-authored by the first author of a previous study that had also been co-authored by one of the developers [28]. Twenty-six studies were conducted by independent researchers.

Three (60%, 3/5) allegiant studies [26], [28], [50] and eleven non-allegiant studies (42%, 11/26) [30]–[32], [34], [37], [38], [51]–[55] were conducted in hospital settings.

Three (60%, 3/5) allegiant studies[12], [26], [50] and thirteen non-allegiant studies (13/26) [30], [37], [38], [42], [48], [52]–[54], [56]–[60], were conducted in English.

The mean prevalence of major depressive disorder in the allegiant group was 13.2% (range 6.1% - 33.5%) and in the non-allegiant group was 16.1% (range 2.5% - 43.2%). The mean age of patients in the allegiant group studies was 48.1 (range 41.9 -61.0) and in the 26 non-allegiant studies that reported these data was 49.1 (range 23.0 – 78.0). The percentage of females in the allegiant studies that reported these data [12], [23], [26], [28] was 56.3% (range 28.6% – 67.8%) and in the non-allegiant group was 64.9 % (range 12% -100%).

For peer review only - http://bmjopen.bmj.com/site/about/guidelines.xhtml

Three allegiant studies used the self-reported mode of administration and two of them did not specify how the PHQ-9 was administered. In 9 non-allegiant studies (34%, 9/26) the PHQ-9 was administered by the researcher [30]–[32], [48], [57], [59]–[62]. All allegiant studies used SCID as a gold standard; the non-allegiant studies used a wider range of gold standards including SCAN, CIDI, MINI, CIS-R, C-DIS, though the SCID was used in half of the studies (50%, 13/26 studies).

Three allegiant studies (60%) did not include a conflict of interests statement [12], [23], [50]. Two of these studies [12], [23] acknowledged funding from Pfizer. None of the allegiant studies acknowledged collaboration or authorship of one of the developers of the PHQ-9.

Of the non-allegiant studies, thirteen (42%) did not include a conflict of interests statement [30]–[32], [37], [42], [46], [48], [54], [56], [61], [63]–[65]. Similar to the algorithm studies, the newer studies were more likely to include a conflict of interest statement. Funding was acknowledged by most studies (27/31) and most received funding from academic or/and health research institutions. One study [58] acknowledged that the last author involved in the development of one of the instruments (CORE-OM), 'but does not gain financially from its use'. One study [52] acknowledged funding from industry, AHA Pharmaceuticals Roundtable, but stated that 'the funding organisations had no role in the design or conduct of the study, collection, management, analysis or interpretation of data; or preparation, review or approval of the manuscript. Fine et al., 2013 disclosed that the last author had financial and consulting interests (Pfizer was not cited as one of them).

Diagnostic test accuracy

Pooled sensitivity of allegiant studies was 0.87 (95% CI = 0.77 – 0.93), pooled specificity was 0.87 (95% CI = 0.76 – 0.94), and the pooled diagnostic odds ratio was 49.31 (95% CI = 25.74 – 94.48) – see table 5. Heterogeneity was moderate (I^2 = 55.1%). Figure 2 represents the summary ROCs for this group.

For peer review only - http://bmjopen.bmj.com/site/about/guidelines.xhtml

----

Figure 2. PHQ-9 summed items scoring method at cut-off point 10 summary ROC plot for diagnosis of major depressive disorder in allegiant studies (panel A) and non-allegiant studies (panel B). Pooled sensitivity and specificity using a bi-variate meta-analysis (*HSROC* hierarchical receiver-operating characteristic).

Pooled sensitivity of non-allegiant studies was 0.76 (95% CI, 0.67 – 0.83), pooled specificity was 0.88 95% CI (0.85 – 0.91), and the pooled diagnostic odds ratio was 24.96 (95% CI 14.81 – 42.08), approximately half that of the allegiant studies (table 2). Heterogeneity was high at I^2 = 81.5 %. Figure 2 represents the summary ROCs for this group.

For peer review only - http://bmjopen.bmj.com/site/about/guidelines.xhtml

The meta-regression for the studies using a cut-off point of 10 or above with allegiance status of the predictor showed that allegiance status was a significant predictor of the diagnostic odds ratio (P = 0.015) and explained 19.0% of observed heterogeneity.

Quality assessment

The results of the quality assessment using the QUADAS-2 are given in table 4. For the patient selection domain, the two groups of studies were broadly comparable on two items (consecutive or random sample, avoid case-control design). However, all allegiant studies were rated as avoiding inappropriate exclusions (5/5) in contrast to 58% (15/26) of the non-allegiant studies.

On the index test domain, there were a number of differences between the two groups of studies. More of the non-allegiant studies (81%, 21/26) reported that the PHQ-9 was interpreted blind to the reference test compared to 60% (3/5) of the allegiant studies. All (5/5) allegiant studies were rated as pre-specifying the threshold on the PHQ-9 compared to 73% (19/26) of the non-allegiant studies. The two sets of studies were broadly comparable in terms of two items from the reference test domain (correctly classify target condition, reference test interpreted blind). Only one allegiant study used a translated version of the index test or reference test, so it is not possible to comment on differences between the two sets of studies in terms of these items from the index or reference test domains. For the flow and timing domain, the two groups of studies were broadly comparable for two of the criteria (interval of two weeks or less, all participants receive same reference test). However, fewer than half of the non-allegiant studies met the criterion for 'all participants included in the analysis' (42%, 11/26); whereas all allegiant studies met this criterion.

Discussion

For peer review only - http://bmjopen.bmj.com/site/about/guidelines.xhtml

This is to our knowledge the first systematic examination of a possible 'allegiance' or authorship effect in the validation of screening or case finding psychological instrument for a common mental health disorder. We reviewed diagnostic validation studies of the PHQ-9, a widely used depression screening-instrument. We found that allegiant studies reported higher sensitivity paired with similar specificity compared to non-allegiant studies. When entered as a covariate in meta-regression analyses, allegiance status was predictive of variation in the DOR for both the algorithm scoring method and the summed-item scoring method at a cut-off point of 10 or above.

Previous research has proposed several possible explanations for the allegiance effect [9]–[11]. One possibility is the advertent bias that may serve to inflate the performance of a test when evaluated by those who have developed it. However, before concluding that the differences are due to this, it is important to explore and rule out alternative explanations. First, it is possible that any observed differences are a result of differences in study characteristics of the two sets of studies (e.g., setting, clinical population). Secondly, differences in the methodological quality of the studies may also account for any differences. These possibilities are examined below.

<u>Difference in study characteristics as potential alternative explanations</u>

The two sets of studies were broadly comparable in terms of gender and the prevalence of depression, so these variables are unlikely to offer an explanation for the differences. While there were some indications from both sets of comparisons that the PHQ-9 may have been researcher-administered more often in the independent studies, it is not immediately clear how this would lead to lowered diagnostic performance.

For peer review only - http://bmjopen.bmj.com/site/about/guidelines.xhtml

The diagnostic meta-analyses of the PHQ-9 [13], [14] have shown that the sensitivity and DOR of the PHQ-9 tends to be lower in hospital settings for both algorithm and summed-item scoring methods. Whilst the fact that proportionally more non-allegiant algorithm studies were conducted in secondary care could explain the lower sensitivity and DOR values in the algorithm studies, in the studies that reported the cut-off point of or above this would not be the case as proportionally more allegiant studies were conducted in hospital settings.

Similarly, differences in the proportions of studies using translated versions of the PHQ-9 are also unlikely to offer an obvious explanation of the difference in diagnostic performance, because in the algorithm set of studies more of the allegiant studies used a translated version of the test, but the proportions were in the opposite direction for the studies using a cut off of 10 or above. We tested this by carrying out a sensitivity analysis restricting the sample to English studies and studies with adequate translation. The allegiance effect was still predictive of DOR variation between allegiance and non-allegiance studies variation in both algorithm ($p = 0.00$) and summed item scoring at cut-off point of 10 meta-analyses ($p = 0.02$).

A similar conclusion is also likely to apply to the age of the samples. There were more older adults studies in the non-allegiant than allegiant studies in the algorithm comparison. Depression could be more difficult to identify in older adults due to physical co-morbidities that may present with similar symptomatology to depression and could account for the lower diagnostic performance in the non-allegiant studies. However, the non-allegiant samples in the studies that reported the psychometric properties at cut-off point 10 or above had younger samples than the allegiant studies, so this would not support this interpretation.

The SCID was used as the gold standard in nearly all allegiant studies. The fact that some non-allegiant studies used other gold standards could potentially explain the poorer psychometric properties of the PHQ-9 in these studies. The SCID is often regarded as the most valid of the available semi-structured interviews used in depression diagnostic validity studies as the reference standard. If we assume that this is the case and, furthermore, that the PHQ-9 is an accurate method of screening for depression, then the PHQ-9 may be more likely to agree with the SCID

For peer review only - http://bmjopen.bmj.com/site/about/guidelines.xhtml

than other reference standards. However, when we carried out a sensitivity analysis restricting the sample to SCID only studies the allegiance effect was still predictive of DOR variation between allegiance and non-allegiance studies variation in both algorithm (p = 0.01) and summed item scoring at cut-off point of 10 reviews (p= 0.02).

Differences in methodological quality as potential alternative explanations

The quality of the studies was evaluated using the QUADAS-2. Although there were several potential methodological differences between the two groups of studies from the algorithm papers, not all of these offer obvious explanations of the observed differences and some are unlikely as explanations. For example, more allegiant studies ensured that the reference test was interpreted blind to the index test. This is unlikely to account for the observed differences, because a lack of blinding is typically associated with artificially increased diagnostic performance, which is in the opposite direction to the pattern of results observed here. The impact of some other differences is less clear-cut. For example, a higher number of the non-allegiant studies met the criterion for consecutive referrals. For this to provide an explanation of the of the observed differences, the non-consecutive nature of the referrals in the studies by those who had developed the PHQ-9 would need to have led to the over-inclusion of true positives or under-inclusion of false negatives given that these studies tended to report higher sensitivity relative to the non-allegiant studies (and vice versa for the independent studies). It is not immediately obvious how this would occur. The allegiant studies were more likely to have met the criterion of 'included all participants in the analysis'. It is possible that the greater loss of participants from the non-allegiant studies may have artificially reduced the observed diagnostic accuracy, though, again, it is not immediately obvious how this would have affected the true positive and false negative rates. Although there is not an obvious explanation of how these differences in methodological quality could account for the observed differences in diagnostic performance, it is important to recognise that they cannot on that basis be ruled out.

For peer review only - http://bmjopen.bmj.com/site/about/guidelines.xhtml

There are, however, two differences in methodological quality among the algorithm studies that are clearer potential alternative explanations. The higher rate of appropriate translations among the allegiant studies is potentially important, because lower diagnostic estimates may be expected from studies that have poorly translated versions of the index test. In the flow and timing domain, more allegiant studies ensured that there was a less than two-week interval between the index and reference test. This is consistent with lower diagnostic performance in the non-allegiant studies: as the interval increases it is likely that depression status may change and this would lead to lower levels of agreement between the index test and the reference test.

There were also differences on some quality assessment items between the two sets of studies in the summed item scoring method comparison. The threshold was reported as pre-specified in all allegiant studies in contrast to approximately three quarters of the non-allegiant studies. On the face of it, this is unlikely to explain the observed differences, because the use of a pre-specified cut-off point is likely to be associated with lower not higher diagnostic test performance. One possibility, however, is that studies that performed poorly at this cut-off point were less likely to be reported by those who had developed the measure. As discussed in more detail in the limitations section, we were unable to explore this possibility through the use of formal tests for publication bias.

All allegiant studies avoided inappropriate exclusions compared to approximately half of the non-allegiant studies. While this is a potential alternative explanation of the differences it is not immediately obvious how this would explain the differences in diagnostic performance between the two sets of studies. Fewer than half of the non-allegiant studies met the criterion for ‘all participants included in the analysis’, in contrast to all of the allegiant studies met this criterion, but again this difference should usually work against the inclusive studies, not those

For peer review only - http://bmjopen.bmj.com/site/about/guidelines.xhtml

excluding cases. More of the non-allegiant studies reported that the PHQ-9 was interpreted blind to the reference test. This does offer a potential explanation, because the absence of blinding may artificially inflate diagnostic accuracy.

Limitations

The results of this review need to be viewed in the light of the limitations of the primary studies that contributed to the review and the review itself. An important consideration is to establish whether any observed differences between the diagnostic performance of the non-allegiant and allegiant studies are better accounted for by study characteristic or methodological differences. Caution, however, is needed in interpreting any differences, because of the small number of allegiant studies in both the algorithm and cut-off 10 or above comparisons. The small number of allegiant studies also meant that we were also unable to explore the potential role of publication bias in the non-allegiant and allegiant studies. At least 10 studies are required to use standard methods of examining publication bias, but the number of allegiant studies in both the algorithm and cut-off 10 or above comparisons were fewer than this.

Conclusions and implications for further research.

The aims of the review was to investigate whether an allegiance effect is found that leads to an increased diagnostic performance in diagnostic validation studies that were conducted by teams connected to the original developers of the PHQ-9. Our analyses showed that diagnostic studies conducted by independent/non-allegiant researchers had lower sensitivity paired with similar specificity compared to studies that were classified as allegiant. This conclusion held for both the algorithm and cut-off 10 or above studies. We explored a range of possible alternative

For peer review only - http://bmjopen.bmj.com/site/about/guidelines.xhtml

explanations for the observed allegiance effect including both differences in study characteristics and study quality. A number of potential differences were found, though for some of these it is not clear how they would necessarily account for the observed differences. However, there were a number of differences that offered potential alternative explanations unconnected to allegiance effects. In the algorithm studies, the studies rated as allegiant were also more likely to use an appropriate translation of the PHQ-9 and were also more likely to ensure that the index and reference test were conducted within two weeks of each other, both of which may be associated with an improvement in observed diagnostic performance of an instrument. The majority of studies in both meta-analyses did not provide clear statements about potential conflict of interest and/or funding, however the newer studies were more likely to provide such statements, which may reflect increasing transparency in this area of research.

We cannot, therefore, conclude that allegiance effects are present in studies examining the diagnostic performance of the PHQ-9; but nor can we rule them out. Conflicts of interest are an important area of investigation in medical and behavioural research, particularly due to concerns about trial results being influenced by industry sponsorship. Future diagnostic validity in this area should as a matter of routine present clear statements about potential conflicts of interest and funding, particularly relating to the development of the instrument under evaluation. Future meta-analyses of diagnostic validation studies of psychological measures should routinely evaluate the impact of researcher allegiance in the primary studies examined in the meta-analysis.

Contributors LM led on all stages of the review and is the guarantor. We used an established database of diagnostic validation studies of the PHQ-9 [13], [14] SG provided expert advice on methodology and approaches to assessment of the evidence base. AM carried out the literature

For peer review only - http://bmjopen.bmj.com/site/about/guidelines.xhtml

searches, screened the studies, extracted data and assessed the quality of the included studies for one of the systematic reviews (Moriarty et al., 2015) . LM carried out the literature searches, screened the studies, extracted data and assessed the quality of the included studies for the other systematic review (Manea et al., 2015), analysed the data for both systematic reviews and drafted the report. JB was involved in the development of the study, wrote the introduction section of the review and contributed to the production of the final report. DM supervised the quality assessment, methodology and approaches to evidence synthesis, provided senior advice and support throughout and contributed to the production of the final report. All parties were involved in drafting and/or commenting on the report.

Competing interests None declared.

Provenance and peer review Not commissioned; externally peer reviewed.

Data sharing statement No additional data are available.

REFERENCES

1. [1] L. Luborsky, L. Diguer, D. A. Seligman, R. Rosenthal, E. D. Krause, S. Johnson, G. Halperin, M. Bishop, J. S. Berman, and E. Schweizer, "The Researcher's Own Therapy Allegiances: A 'Wild Card' in Comparisons of Treatment Efficacy," *Clin. Psychol. Sci. Pract.*, vol. 6, no. 1, pp. 95–106, May 2006.

For peer review only - http://bmjopen.bmj.com/site/about/guidelines.xhtml

[2] E. Dragioti, I. Dimoliatis, and E. Evangelou, “Disclosure of researcher allegiance in meta-analyses and randomised controlled trials of psychotherapy: a systematic appraisal,” *BMJ Open*, vol. 5, no. 6, pp. e007206–e007206, Jun. 2015.

[3] T. Munder, O. Brütsch, R. Leonhart, H. Gerger, and J. Barth, “Researcher allegiance in psychotherapy outcome research: An overview of reviews,” *Clin. Psychol. Rev.*, vol. 33, no. 4, pp. 501–511, Jun. 2013.

[4] D. A. Winter, “Editorial.” Routledge, 07-May-2010.

[5] J. McLeod, “Taking allegiance seriously–implications for research policy and practice,” *Eur. J. Psychother. Couns.*, May 2010.

[6] G. L. Staines and C. M. Cleland, “Bias in meta-analytic estimates of the absolute efficacy of psychotherapy.,” *Rev. Gen. Psychol.*, vol. 11, no. 4, pp. 329–347, 2007.

[7] K. D. Markman and E. R. Hirt, “Social Prediction and the ‘Allegiance Bias,’” *Soc. Cogn.*, vol. 20, no. 1, pp. 58–86, Feb. 2002.

[8] G. D. Walters, “The Psychological Inventory of Criminal Thinking Styles and Psychopathy Checklist: Screening version as incrementally valid predictors of recidivism.,” *Law Hum. Behav.*, vol. 33, no. 6, pp. 497–505, 2009.

[9] J. P. Singh, M. Grann, and S. Fazel, “Authorship Bias in Violence Risk Assessment? A Systematic Review and Meta-Analysis,” *PLoS One*, vol. 8, no. 9, p. e72484, Sep. 2013.

[10] P. R. Blair, D. K. Marcus, and M. T. Boccaccini, “Is There an Allegiance Effect for Assessment Instruments? Actuarial Risk Assessment as an Exemplar,” *Clin. Psychol. Sci. Pract.*, vol. 15, no. 4, pp. 346–360, Oct. 2008.

[11] S. O. Lilienfeld and M. K. Jones, “Allegiance Effects in Assessment: Unresolved Questions, Potential Explanations, and Constructive Remedies,” *Clin. Psychol. Sci. Pract.*, vol. 15, no. 4, pp. 361–365, Oct. 2008.

For peer review only

For peer review only - http://bmjopen.bmj.com/site/about/guidelines.xhtml

[12] K. Kroenke, R. L. Spitzer, and J. B. Williams, “The PHQ-9: validity of a brief depression severity measure.,” *J. Gen. Intern. Med.*, vol. 16, no. 9, pp. 606–13, Sep. 2001.

[13] L. Manea, S. Gilbody, and D. McMillan, “A diagnostic meta-analysis of the Patient Health Questionnaire-9 (PHQ-9) algorithm scoring method as a screen for depression,” *Gen. Hosp. Psychiatry*, vol. 37, no. 1, pp. 67–75, Jan. 2015.

[14] A. S. Moriarty, S. Gilbody, D. McMillan, and L. Manea, “Screening and case finding for major depressive disorder using the Patient Health Questionnaire (PHQ-9): a meta-analysis,” *Gen. Hosp. Psychiatry*, vol. 37, no. 6, pp. 567–576, Nov. 2015.

[15] P. F. Whiting, A. W. S. Rutjes, M. E. Westwood, S. Mallett, J. J. Deeks, J. B. Reitsma, M. M. G. Leeflang, J. A. C. Sterne, P. M. M. Bossuyt, and QUADAS-2 Group, “QUADAS-2: a revised tool for the quality assessment of diagnostic accuracy studies.,” *Ann. Intern. Med.*, vol. 155, no. 8, pp. 529–36, Oct. 2011.

[16] R. Mann, C. E. Hewitt, and S. M. Gilbody, “Assessing the quality of diagnostic studies using psychometric instruments: applying QUADAS,” *Soc. Psychiatry Psychiatr. Epidemiol.*, vol. 44, no. 4, pp. 300–307, Apr. 2009.

[17] J. B. Reitsma, A. S. Glas, A. W. S. Rutjes, R. J. P. M. Scholten, P. M. Bossuyt, and A. H. Zwinderman, “Bivariate analysis of sensitivity and specificity produces informative summary measures in diagnostic reviews,” *J. Clin. Epidemiol.*, vol. 58, no. 10, pp. 982–990, Oct. 2005.

[18] University of York. NHS Centre for Reviews and Dissemination., *Systematic reviews : CRD’s guidance for undertaking reviews in health care*. CRD, University of York, 2009.

[19] S. D. Walter, “Properties of the summary receiver operating characteristic (SROC) curve for diagnostic test data,” *Stat. Med.*, vol. 21, no. 9, pp. 1237–1256, May 2002.

For peer review only

For peer review only - http://bmjopen.bmj.com/site/about/guidelines.xhtml

[20] J. G. Lijmer, P. M. M. Bossuyt, and S. H. Heisterkamp, “Exploring sources of heterogeneity in systematic reviews of diagnostic tests,” *Stat. Med.*, vol. 21, no. 11, pp. 1525–1537, Jun. 2002.

[21] S. G. Thompson and J. P. T. Higgins, “How should meta-regression analyses be undertaken and interpreted?,” *Stat. Med.*, vol. 21, no. 11, pp. 1559–1573, Jun. 2002.

[22] C. Diez-Quevedo, T. Rangil, L. Sanchez-Planell, K. Kroenke, and R. L. Spitzer, “Validation and utility of the patient health questionnaire in diagnosing mental disorders in 1003 general hospital Spanish inpatients.,” *Psychosom. Med.*, vol. 63, no. 4, pp. 679–86.

[23] K. Gräfe, S. Zipfel, W. Herzog, and B. Löwe, “Screening psychischer Störungen mit dem “Gesundheitsfragebogen für Patienten (PHQ-D)“,” *Diagnostica*, vol. 50, no. 4, pp. 171–181, Oct. 2004.

[24] B. Löwe, R. L. Spitzer, K. Gräfe, K. Kroenke, A. Quenter, S. Zipfel, C. Buchholz, S. Witte, and W. Herzog, “Comparative validity of three screening questionnaires for DSM-IV depressive disorders and physicians’ diagnoses.,” *J. Affect. Disord.*, vol. 78, no. 2, pp. 131–40, Feb. 2004.

[25] R. L. Spitzer, K. Kroenke, and J. B. Williams, “Validation and utility of a self-report version of PRIME-MD: the PHQ primary care study. Primary Care Evaluation of Mental Disorders. Patient Health Questionnaire.,” *JAMA*, vol. 282, no. 18, pp. 1737–44, Nov. 1999.

[26] P. Thekkumpurath, J. Walker, I. Butcher, L. Hodges, A. Kleiboer, M. O’Connor, L. Wall, G. Murray, K. Kroenke, and M. Sharpe, “Screening for major depression in cancer outpatients: the diagnostic accuracy of the 9-item patient health questionnaire.,” *Cancer*, vol. 117, no. 1, pp. 218–27, Jan. 2011.

[27] K. Muramatsu, H. Miyaoka, K. Kamijima, Y. Muramatsu, M. Yoshida, T. Otsubo, and F. Gejyo, “The patient health questionnaire, Japanese version: validity according to the mini-international neuropsychiatric interview-plus.,” *Psychol. Rep.*, vol. 101, no. 3 Pt 1, pp.

For peer review only - http://bmjopen.bmj.com/site/about/guidelines.xhtml

952–60, Dec. 2007.

[28] R. Navinés, P. Castellví, J. Moreno-España, D. Gimenez, M. Udina, S. Cañizares, C. Diez-Quevedo, M. Valdés, R. Solà, and R. Martín-Santos, "Depressive and anxiety disorders in chronic hepatitis C patients: Reliability and validity of the Patient Health Questionnaire," *J. Affect. Disord.*, vol. 138, no. 3, pp. 343–351, May 2012.

[29] S. M. Eack, C. G. Greeno, and B.-J. Lee, "Limitations of the Patient Health Questionnaire in Identifying Anxiety and Depression: Many Cases Are Undetected.," *Res. Soc. Work Pract.*, vol. 16, no. 6, pp. 625–631, Nov. 2006.

[30] J. R. Fann, C. H. Bombardier, S. Dikmen, P. Esselman, C. A. Warms, E. Pelzer, H. Rau, and N. Temkin, "Validity of the Patient Health Questionnaire-9 in assessing depression following traumatic brain injury.," *J. Head Trauma Rehabil.*, vol. 20, no. 6, pp. 501–11.

[31] B. Gelaye, M. A. Williams, S. Lemma, N. Deyessa, Y. Bahretibeb, T. Shibre, D. Wondimagegn, A. Lemenhe, J. R. Fann, A. Vander Stoep, and X.-H. H. Andrew Zhou, "Validity of the patient health questionnaire-9 for depression screening and diagnosis in East Africa," *Psychiatry Res.*, vol. 210, no. 2, pp. 653–661, Dec. 2013.

[32] T. Hyphantis, K. Kotsis, P. V. Voulgari, N. Tsifetaki, F. Creed, and A. A. Drosos, "Diagnostic accuracy, internal consistency, and convergent validity of the Greek version of the patient health questionnaire 9 in diagnosing depression in rheumatologic disorders," *Arthritis Care Res. (Hoboken).*, vol. 63, no. 9, pp. 1313–1321, Sep. 2011.

[33] M. Inagaki, T. Ohtsuki, N. Yonemoto, Y. Kawashima, A. Saitoh, Y. Oikawa, M. Kurosawa, K. Muramatsu, T. A. Furukawa, and M. Yamada, "Validity of the Patient Health Questionnaire (PHQ)-9 and PHQ-2 in general internal medicine primary care at a Japanese rural hospital: a cross-sectional study.," *Gen. Hosp. Psychiatry*, vol. 35, no. 6, pp. 592–7, Jan. 2013.

[34] M. E. Khamseh, H. R. Baradaran, A. Javanbakht, M. Mirghorbani, Z. Yadollahi, and M. Malek, "Comparison of the CES-D and PHQ-9

For peer review only

depression scales in people with type 2 diabetes in Tehran, Iran," *BMC Psychiatry*, vol. 11, no. 1, p. 61, Dec. 2011.

[35] P. Persoons, K. Luyckx, C. Desloovere, J. Vandenberghe, and B. Fischler, "Anxiety and mood disorders in otorhinolaryngology outpatients presenting with dizziness: validation of the self-administered PRIME-MD Patient Health Questionnaire and epidemiology.," *Gen. Hosp. Psychiatry*, vol. 25, no. 5, pp. 316–23.

[36] A. Picardi, D. A. Adler, D. Abeni, H. Chang, P. Pasquini, W. H. Rogers, and K. M. Bungay, "Screening for depressive disorders in patients with skin diseases: a comparison of three screeners.," *Acta Derm. Venereol.*, vol. 85, no. 5, pp. 414–9, 2005.

[37] L. Stafford, M. Berk, and H. J. Jackson, "Validity of the Hospital Anxiety and Depression Scale and Patient Health Questionnaire-9 to screen for depression in patients with coronary artery disease," *Gen. Hosp. Psychiatry*, vol. 29, no. 5, pp. 417–424, Sep. 2007.

[38] B. D. Thombs, R. C. Ziegelstein, and M. A. Whooley, "Optimizing detection of major depression among patients with coronary artery disease using the patient health questionnaire: data from the heart and soul study.," *J. Gen. Intern. Med.*, vol. 23, no. 12, pp. 2014–7, Dec. 2008.

[39] A. W. Thompson, H. Liu, R. D. Hays, W. J. Katon, R. Rausch, N. Diaz, E. L. Jacob, S. D. Vassar, and B. G. Vickrey, "Diagnostic accuracy and agreement across three depression assessment measures for Parkinson's disease," *Parkinsonism Relat. Disord.*, vol. 17, no. 1, pp. 40–45, Jan. 2011.

[40] A. Turner, J. Hambridge, J. White, G. Carter, K. Clover, L. Nelson, and M. Hackett, "Depression screening in stroke: a comparison of alternative measures with the structured diagnostic interview for the diagnostic and statistical manual of mental disorders, fourth edition (major depressive episode) as criterion standard.," *Stroke.*, vol. 43, no. 4, pp. 1000–5, Apr. 2012.

[41] K. M. van Steenbergen-Weijenburg, L. de Vroege, R. R. Ploeger, J. W. Brals, M. G. Vloedbeld, T. F. Veneman, L. Hakkaart-van Roijen,

For peer review only - http://bmjopen.bmj.com/site/about/guidelines.xhtml

F. F. Rutten, A. T. Beekman, and C. M. van der Feltz-Cornelis, “Validation of the PHQ-9 as a screening instrument for depression in diabetes patients in specialized outpatient clinics,” *BMC Health Serv. Res.*, vol. 10, no. 1, p. 235, Dec. 2010.

[42] B. Arroll, F. Goodyear-Smith, S. Crengle, J. Gunn, N. Kerse, T. Fishman, K. Falloon, and S. Hatcher, “Validation of PHQ-2 and PHQ-9 to Screen for Major Depression in the Primary Care Population,” *Ann. Fam. Med.*, vol. 8, no. 4, pp. 348–353, Jul. 2010.

[43] L. Ayalon, M. Goldfracht, and P. Bech, “‘Do you think you suffer from depression?’ Reevaluating the use of a single item question for the screening of depression in older primary care patients,” *Int. J. Geriatr. Psychiatry*, vol. 25, no. 5, pp. 497–502, May 2010.

[44] V. Henkel, R. Mergl, R. Kohnen, A.-K. Allgaier, H.-J. Möller, and U. Hegerl, “Use of brief depression screening tools in primary care: consideration of heterogeneity in performance in different patient groups,” *Gen. Hosp. Psychiatry*, vol. 26, no. 3, pp. 190–198, May 2004.

[45] F. Lamers, C. C. M. Jonkers, H. Bosma, B. W. J. H. Penninx, J. A. Knottnerus, and J. T. M. van Eijk, “Summed score of the Patient Health Questionnaire-9 was a reliable and valid method for depression screening in chronically ill elderly patients,” *J. Clin. Epidemiol.*, vol. 61, no. 7, pp. 679–687, Jul. 2008.

[46] M. Lotrakul, S. Sumrithe, and R. Saipanish, “Reliability and validity of the Thai version of the PHQ-9,” *BMC Psychiatry*, vol. 8, no. 1, p. 46, Dec. 2008.

[47] N. P. Zuithoff, Y. Vergouwe, M. King, I. Nazareth, M. J. van Wezep, K. G. Moons, and M. I. Geerlings, “The Patient Health Questionnaire-9 for detection of major depressive disorder in primary care: consequences of current thresholds in a crosssectional study,” *BMC Fam. Pract.*, vol. 11, no. 1, p. 98, Dec. 2010.

[48] D. Gjerdingen, S. Crow, P. McGovern, M. Miner, and B. Center, “Postpartum depression screening at well-child visits: validity of a 2-question screen and the PHQ-9.,” *Ann. Fam. Med.*, vol. 7, no. 1, pp. 63–70, 2009.

For peer review only - http://bmjopen.bmj.com/site/about/guidelines.xhtml

[49] V. Henkel, R. Mergl, R. Kohnen, A.-K. Allgaier, H.-J. Möller, and U. Hegerl, “Use of brief depression screening tools in primary care: consideration of heterogeneity in performance in different patient groups.,” *Gen. Hosp. Psychiatry*, vol. 26, no. 3, pp. 190–8.

[50] L. S. Williams, E. J. Brizendine, L. Plue, T. Bakas, W. Tu, H. Hendrie, and K. Kroenke, “Performance of the PHQ-9 as a screening tool for depression after stroke.,” *Stroke.*, vol. 36, no. 3, pp. 635–8, Mar. 2005.

[51] M. H. N. Chagas, V. Tumas, G. R. Rodrigues, J. P. Machado-de-Sousa, A. S. Filho, J. E. C. Hallak, and J. A. S. Crippa, “Validation and internal consistency of Patient Health Questionnaire-9 for major depression in Parkinson’s disease,” *Age Ageing*, vol. 42, no. 5, pp. 645–649, Sep. 2013.

[52] L. Elderon, K. G. Smolderen, B. Na, and M. A. Whooley, “Accuracy and prognostic value of American Heart Association: recommended depression screening in patients with coronary heart disease: data from the Heart and Soul Study.,” *Circ. Cardiovasc. Qual. Outcomes*, vol. 4, no. 5, pp. 533–40, Sep. 2011.

[53] A. G. Rooney, S. McNamara, M. Mackinnon, M. Fraser, R. Rampling, A. Carson, and R. Grant, “Screening for major depressive disorder in adults with cerebral glioma: an initial validation of 3 self-report instruments,” *Neuro. Oncol.*, vol. 15, no. 1, pp. 122–129, Jan. 2013.

[54] S. Watnick, P.-L. Wang, T. Demadura, and L. Ganzini, “Validation of 2 depression screening tools in dialysis patients.,” *Am. J. Kidney Dis.*, vol. 46, no. 5, pp. 919–24, Nov. 2005.

[55] Y. Zhang, R. Ting, M. Lam, J. Lam, H. Nan, R. Yeung, W. Yang, L. Ji, J. Weng, Y.-K. Wing, N. Sartorius, and J. C. N. Chan, “Measuring depressive symptoms using the Patient Health Questionnaire-9 in Hong Kong Chinese subjects with type 2 diabetes,” *J. Affect. Disord.*, vol. 151, no. 2, pp. 660–666, Nov. 2013.

[56] A. O. Adewuya, B. A. Ola, and O. O. Afolabi, “Validity of the patient health questionnaire (PHQ-9) as a screening tool for depression

For peer review only - http://bmjopen.bmj.com/site/about/guidelines.xhtml

amongst Nigerian university students," *J. Affect. Disord.*, vol. 96, no. 1–2, pp. 89–93, Nov. 2006.

[57] T. H. Fine, A. A. Contractor, M. Tamburrino, J. D. Elhai, M. R. Prescott, G. H. Cohen, E. Shirley, P. K. Chan, T. Goto, R. Slembarski, I. Liberzon, S. Galea, and J. R. Calabrese, "Validation of the telephone-administered PHQ-9 against the in-person administered SCID-I major depression module," *J. Affect. Disord.*, vol. 150, no. 3, pp. 1001–1007, Sep. 2013.

[58] S. Gilbody, D. Richards, S. Brealey, and C. Hewitt, "Screening for depression in medical settings with the Patient Health Questionnaire (PHQ): A diagnostic meta-analysis," *J. Gen. Intern. Med.*, vol. 22, no. 11, pp. 1596–1602, Oct. 2007.

[59] E. Phelan, B. Williams, K. Meeker, K. Bonn, J. Frederick, J. LoGerfo, and M. Snowden, "A study of the diagnostic accuracy of the PHQ-9 in primary care elderly," *BMC Fam. Pract.*, vol. 11, no. 1, p. 63, Dec. 2010.

[60] A. C. Sidebottom, P. A. Harrison, A. Godecker, and H. Kim, "Validation of the Patient Health Questionnaire (PHQ)-9 for prenatal depression screening," *Arch. Womens. Ment. Health*, vol. 15, no. 5, pp. 367–374, Oct. 2012.

[61] F. de Lima Osório, A. Vilela Mendes, J. A. Crippa, and S. R. Loureiro, "Study of the Discriminative Validity of the PHQ-9 and PHQ-2 in a Sample of Brazilian Women in the Context of Primary Health Care," *Perspect. Psychiatr. Care*, vol. 45, no. 3, pp. 216–227, Jul. 2009.

[62] V. Patel, R. Araya, N. Chowdhary, M. King, B. Kirkwood, S. Nayak, G. Simon, and H. A. Weiss, "Detecting common mental disorders in primary care in India: a comparison of five screening questionnaires," *Psychol. Med.*, vol. 38, no. 2, Feb. 2008.

[63] M. N. N. Azah, M. E. M. Shah, S. Juwita, I. S. Bahri, W. M. W. M. Rushidi, and Y. M. Jamil, "Validation of the Malay Version Brief Patient Health Questionnaire (PHQ-9) among Adult Attending Family Medicine Clinics.," *Int. Med. J.*, 2005.

[64] S.-I. Liu, Z.-T. Yeh, H.-C. Huang, F.-J. Sun, J.-J. Tjung, L.-C. Hwang, Y.-H. Shih, and A. W.-C. Yeh, "Validation of Patient Health Questionnaire for depression screening among primary care patients in Taiwan," *Compr. Psychiatry*, vol. 52, no. 1, pp. 96–101, Jan. 2011.

For peer review only

For peer review only - http://bmjopen.bmj.com/site/about/guidelines.xhtml

[65] K. Wittkampf, H. van Ravesteijn, K. Baas, H. van de Hoogen, A. Schene, P. Bindels, P. Lucassen, E. van de Lisdonk, and H. van Weert, "The accuracy of Patient Health Questionnaire-9 in detecting depression and measuring depression severity in high-risk groups in primary care," *Gen. Hosp. Psychiatry*, vol. 31, no. 5, pp. 451–459, Sep. 2009.

For peer review only

For peer review only - http://bmjopen.bmj.com/site/about/guidelines.xhtml

Table 1: Descriptive characteristics of algorithm studies (Manea et al., 2014)

Study	Sample characteristics (Country, setting, age, sex)	Sample size and % depressed	PHQ-9 characteristics	Diagnostic standard	a) Conflict of interest (COI) declaration<br>b) Funding<br>c) Relationship with original developers
Diez-Quevedo et al. (2001)	Country: Spain<br><br>Setting: Medical and surgical tertiary hospitals<br><br>Age (yrs): M=43 (SD=14.2)<br><br>Female: 45.6%	N = 1003<br>Depressed: 8.2%	Administration: Self-report<br>Language: Spanish	DSM-III-R SCID	a) No COI declaration<br>b) Funding acknowledged (academic institutions)<br>c) Not acknowledged
Gräfe et al. (2004)	Country: Germany<br><br>Setting: psychosomatic walk-in clinics and family practices<br><br>Age (yrs): M = 41.9 (SD = 13.8)<br><br>Female: 67.8%	N = 528<br><br>Depressed: 29.2% psychosomatic patients; 6.16% medical patients	Language: German<br><br>Administration: self-report	DSM-IV SCID	a) No COI declaration<br>b) Acknowledged funding from Pfizer<br>c) Not acknowledged

For peer review only

For peer review only - http://bmjopen.bmj.com/site/about/guidelines.xhtml

Lowe et al. (2004)	Country: Germany<br><br>Setting: Outpatient clinics and family practices<br>Age (yrs): M = 41.7 (SD = 13.8)<br>Female: 67.1%	N = 501<br><br>Depressed: 13.2%	Administration: Self-report<br>Language: German	DSM–IV SCID	a) COI declaration 'This study was supported by unrestricted restricted grants from Pfizer Germany and from the medical faculty of the University of Heidelberg Germany, and there are no COI.'<br>b) Acknowledged funding from Pfizer and academic institution<br>c) Not acknowledged
Muramatsu et al. (2007)	Country: Japan<br><br>Setting: Primary care and general hospital<br><br>Age (yrs): M = 43.3 (SD = 16.4)<br><br>Female: 59.5%	N = 131<br><br>Depressed: 28.2%	Administration: Self-report<br><br>Language: Japanese	DSM–IV MINI	a) No COI declaration<br>b) Acknowledged funding from Pfizer<br>c) Acknowledged one of the developers of the PHQ-9: 'The authors acknowledge Dr R L Spitzer'
Navinés et al. (2012)	Country: Spain<br><br>Setting: General hospital (patients with chronic HCV)<br><br>Age (yrs): M = 43.4 (SD = 10.2)<br><br>Female: 28.6%	N = 500<br>Depressed: 6.4%	Administration: Self-report<br><br>Language: Spanish	DSM–IV SCID	a) All authors declared that they had no COI.<br>b) Role of funding source declared<br>c) Not acknowledged

For peer review only - http://bmjopen.bmj.com/site/about/guidelines.xhtml

Spitzer et al. (1999)	Country: US<br><br>Setting: Primary care<br><br>Age (yrs): M = 46 (SD = 17.2)<br><br>Female: 66%	N = 3000 (585 received SCID)<br><br>Depressed: 10%	Administration: Self-report<br><br>Language: English	DSM-III-R SCID	a) No COI declaration<br>b) Acknowledged funding from Pfizer. 'Drs Spitzer and Williams receive honoraria and consulting money from Pfizer Inc, which has supported this work.'<br>c) N/A
Thekkumpurath et al. (2010)	Country: UK<br><br>Setting: Hospital (cancer patients)<br><br>Age (yrs): M = 61<br><br>Female: 63%	N = 782<br><br>Depressed: 6.3% (of the whole sample)	Administration: Not stated<br><br>Language: English	DSM-IV SCID	a) COI declaration: 'Supported by Cancer Research UK'<br>b) As in a)<br>c) Not acknowledged
Ayalon et al. (2010)	Country: Israel<br><br>Age (yrs): M = 75 (SD = 8.1)<br><br>Female: 40.5 %	N = 153<br>Depressed: 3.9 %	Administration: Researcher administered<br><br>Language: Hebrew	DSM-IV SCID	a) COI declaration: 'The project was funded by an Investigator's Initiated Research Grant from Lundbeck International given to Dr Liat Ayalon. Lundbeck International had no other involvement in the project concept of design or in this paper. Per Bech has occasionally over the past 3 years until August 2008 received funding from and has been speaker or member of advisory boards for pharmaceutical companies with an interest in the drug treatment of affective disorders (Astra-Zeneca, Lilly, H. Lundbeck A/S, Lundbeck Foundation and Organon). '<br>b) Acknowledged funding from Lundbeck International

For peer review only - http://bmjopen.bmj.com/site/about/guidelines.xhtml

Eack et al. (2006)	Country: US<br><br>Setting: Community mental health centers for children<br><br>Age (yrs): M = 39.20 (SD 9.63)<br><br>Female: 100%	N = 50<br><br>Depressed: 28%	Administration: Self-report<br><br>Language: English	DSM-IV SCID	a) No COI declaration<br>b) Funding acknowledged (academic /health research institutions)
Fann et al. (2005)	Country: US<br><br>Setting: Trauma hospital (inpatients with traumatic brain injury)<br><br>Age (yrs): M = 42 (SD=17.9)<br><br>Female: 29.1%	N = 135<br><br>Depressed: 16.3%	Administration: Telephone-administered<br><br>Language: English	DSM-IV SCID	a) No COI declaration<br>b) Funding acknowledged (academic institutions)

For peer review only - http://bmjopen.bmj.com/site/about/guidelines.xhtml

Gelaye et al. (2011)	Country: Ethiopia<br><br>Setting: General hospital<br><br>Age (yrs): 34.9 (SD=11.6)<br><br>Female: 63.1 %	N = 363<br>Depressed: 12.6%	Administration: Researcher-administered<br><br>Language: Amharic	DSM-IV<br>SCAN	a) No COI declaration<br>b) Funding acknowledged (academic /health research institutions)
Gjerdingen et al. (2009)	Country: US<br><br>Setting: Community<br><br>Age (yrs): M = 29.3<br><br>Female: 100%	N = 438<br><br>Depressed: 4.6%	Administration: Telephone or self-report<br><br>Language: English	DSM-IV<br>SCID	a) No COI declaration<br>b) Funding acknowledged (academic /health research institutions)
Henkel et al. (2004)	Country: Germany<br><br>Setting: primary care<br><br>Age (yrs): not reported<br><br>Female: 74%	N = 448<br><br>Depressed: 10%	Administration: self-report<br><br>Language: German	DSM-IV<br>CIDI	a) No COI declaration<br>b) Funding acknowledged (academic /health research institutions)

For peer review only - http://bmjopen.bmj.com/site/about/guidelines.xhtml

Hyphantis et al. (2011)	Country: Greece<br><br>Setting: Hospital – rheumatology patients<br><br>Age (yrs): M = 54.2 (SD = 13.5)<br><br>Female: 74%	N = 213<br><br>Depressed: 32.4%	Administration: Researcher administered<br><br>Language: Greek	DSM-IV MINI	a) No COI declaration<br>b) No funding acknowledgement
Inagaki et al. (2013)	Country: Japan<br><br>Setting: General hospital<br><br>Age whole sample (yrs): M = 73.5 (SD = 12.3)<br><br>Female: 59.3%	N = 104 out of 511 received MINI<br><br>Depressed: 7.4%	Administration: Researcher administered<br>Language: Japanese	DSM-IV MINI	a) COI declaration: 'The authors declare that they have no competing interests.'<br><br>b) Funding acknowledged (academic /health research institutions)
Khamseh et al. (2011)	Country: Iran<br><br>Setting: Diabetes clinic<br><br>Age (yrs): M = 56.17 (SD = 9.60)<br><br>Female: 51.9%	N = 185<br><br>Depressed: 43.2%	Administration: Self report<br>Language: Persian	DSM-IV SCID	a) COI declaration: The authors declared no competing interests<br>b) Funding acknowledged (academic /health research institutions)

For peer review only - http://bmjopen.bmj.com/site/about/guidelines.xhtml

Lamers et al. (2008)	Country: Netherlands<br><br>Setting: Primary care (elderly)<br><br>Age (yrs): M = 71.4 (SD = 6.90)<br><br>Female: 48.2%	N = 713<br><br>Depressed: 10.7%	Administration: Self report<br><br>Language: Dutch	DSM-IV MINI	a) No COI declaration<br>b) Funding acknowledged (academic /health research institutions)
Lotrakul et al. (2008)	Country: Thailand<br><br>Setting: Primary care<br><br>Age (yrs): M = 45.0 (SD = 14.30)<br><br>Female: 73.7%	N = 279<br><br>Depressed: 6.8%	Administration: Self report<br><br>Language: Thai	DSM-IV MINI	a) No COI declaration<br>b) Funding acknowledged (academic /health research institutions)
Persoons et al. (2003)	Country: Belgium<br><br>Setting: Hospital (otolaryngology patients)<br><br>Age (yrs): M = 48.2 (SD = 12.9)<br><br>Female: 65.6%	N = 268 (97 received MINI)<br><br>Depressed: 16.5%	Administration: Self-report<br><br>Language: Dutch	DSM-IV MINI	a) No COI declaration<br>b) Funding acknowledged (academic /health research institutions) and Pfizer Belgium

For peer review only

Picardi et al. (2005)	Country: Italy<br><br>Setting: Hospital (dermatology inpatients)<br><br>Age (yrs): M = 37.5<br><br>Female: 56%	N = 141<br><br>Depressed: 8.5%	Administration: Self-report<br><br>Language: Italian	DSM-IV SCID	a) No COI declaration<br>b) Funding acknowledged (academic /health research institutions). Acknowledged Pfizer Italia SRL for providing the Italian version of the PHQ-9 and for permission to use it.
Stafford et al. (2007)	Country: Australia<br><br>Setting: Hospital (cardiology patients)<br><br>Age (yrs): M = 64.1 (SD = 10.3)<br><br>Female: 66%	N = 193<br><br>Depressed: 18%	Administration: Self-report<br><br>Language: English	DSM–IV MINI	a) No COI declaration<br>b) Funding acknowledged (academic/health research institutions)
Thombs et al. (2008)	Country: US<br><br>Setting: Hospital (outpatients with coronary heart disease)<br><br>Age (yrs): M = 67 (SD = 11)<br><br>Female: 18%	N = 1024<br><br>Depressed: 22%	Administration: Not stated<br><br>Language: English	DSM C-DIS	a) COI declaration "None disclosed"<br>b) Funding acknowledged (academic/health research institutions)

For peer review only - http://bmjopen.bmj.com/site/about/guidelines.xhtml

Thompson et al. (2010)	Country: US<br><br>Setting: Patients with Parkinson Disease<br><br>Age (yrs): 72.5 (SD = 9.6)<br><br>Female: 42%	N = 214<br><br>Depressed: 14%	Administration: Self administered<br><br>Language: English	DSM-IV SCID	a) No COI declaration<br>b) Funding acknowledged (academic/health research institutions)
Turner et al. (2012)	Country: Australia<br><br>Setting: Stroke patients<br><br>Age (yrs): 66.7 (SD = 13.1)<br><br>Female: 47.2%	N = 72<br><br>Depressed: 18%	Administration: Self administered<br><br>Language: English	DSM-IV SCID	a) COI declaration: Disclosures 'None'.<br>b) Funding acknowledged (academic/health research institutions)

For peer review only

For peer review only - http://bmjopen.bmj.com/site/about/guidelines.xhtml

van Steenbergen-Weijenburg (2010)	Country: Netherlands<br><br>Setting: Diabetes patients<br><br>Age (yrs): M = 61.8 (SD = 13.6)<br><br>Female: 48.7%	N = 197<br>Depressed: 18.8%	Administration: Self administered<br><br>Language: Dutch	DSM-IV<br>SCID	a) COI declaration: 'The authors declare that they have no competing interests'.<br>b) Funding acknowledged (academic/health research institutions) – 'this had no influence on the content of this article'.
Zuitthoff et al. (2010)	Country: Netherlands<br><br>Setting: Primary care<br><br>Age (yrs): M = 51 (sd = 16.7)<br><br>Female: 63%	N = 1338<br><br>Depressed: 13%	Administration: Self-report<br><br>Language: Dutch	DSM-IV<br>CIDI	a) COI declaration 'The authors declare that they have no competing interests.'<br>b) Funding acknowledged (academic/health research institutions).

For peer review only - http://bmjopen.bmj.com/site/about/guidelines.xhtml

Table 2: Descriptive characteristics of the summed items scoring method studies cut-off point 10 (Moriarty et al, 2015)

Study	Sample characteristics	Sample size and % MDD	PHQ-9 characteristics	Diagnostic standard	Conflict of interest (COI) declaration<br>Funding<br>c) Relationship with original developers
13. Gräfe et al. (2004)	Country: Germany<br><br>Setting: psychosomatic walk-in clinics and family practices<br><br>Mean age: 41.9 (SD = 13.8)<br><br>Female: 67.8%	N = 528<br><br>Depressed: 29.2% psychosomatic patients; 6.16% medical patients	Administration: self-report<br><br>Language: German<br><br>Cut-offs: 10 to 14	DSM-IV SCID	No COI declaration<br>Acknowledged funding from Pfizer<br>Not acknowledged
16. Kroenke et al. (2001)	Country: USA<br><br>Setting: Primary care<br><br>Mean age: 46 (SD=17)<br><br>Female: 66%	N = 580<br><br>7.1% MDD	Administration: Self-report<br><br>Language: English<br><br>Cut-offs: 9 to 15	DSM-IV SCID	a) No COI declaration<br>b) Acknowledged funding from Pfizer<br>c) N/A
22. Navinés et al. (2012)	Country: Spain<br><br>Setting: General hospital (patients with chronic HCV)<br><br>Mean age: 43.4 (SD = 10.2)<br><br>Female: 28.6%	N = 500<br><br>6.4% MDD	Administration: Self-report<br><br>Language: Spanish<br><br>Cut-offs: 10	DSM–IV SCID	`a) All authors declared that they had no COI.<br>b) Role of funding source declared<br>c) Not acknowledged
29. Thekkumpurath et al. (2010)	Country: UK<br><br>Setting: Hospital (cancer patients)<br><br>Mean age: 61<br><br>Female: 63%	N = 782<br><br>6.3% MDD (of the whole sample)	Administration: Not stated<br><br>Language: English<br><br>Cut-offs: 5 to 10	DSM-IV SCID	c) COI declaration: 'Supported by Cancer Research UK'<br>d) As in a)<br>e) Not acknowledged
33. Williams et al. (2005)	Country: USA	N = 316	Administration: Unclear	DSM-IV SCID	a) No COI declaration<br>b) Funding

For peer review only

For peer review only - http://bmjopen.bmj.com/site/about/guidelines.xhtml

	Setting: Secondary care (Post-stroke)<br><br>Mean age: Unclear<br><br>Female: Unclear	33.5% MDD	Language: English<br><br>Cut-offs: 10		acknowledged (academic institutions)<br>c) Not acknowledged

1. Adewuya et al. (2006)	Country: Nigeria<br><br>Setting: community (students)<br><br>Mean age: 24.8 (15-40)<br><br>Female: 41.2%	N = 512<br><br>2.5% MDD	Administration: Self-report<br><br>Language: English<br><br>Cut-offs: 8 to 12	DSM-IV MINI	a) No COI declaration<br>b) No funding declaration
2. Arroll et al. (2010)	Country: New Zealand<br><br>Setting: Primary care<br><br>Mean age: 49 (17-99)<br><br>Female: 61%	N = 2642<br><br>6.2% MDD	Administration: Not stated<br><br>Language: English<br><br>Cut-offs: 8,10,12,15	DSM-IV SCID	a) No COI declaration<br>b) Funding acknowledged (academic /health research institutions)
3. Azah et al. (2005)	Country: Malaysia<br><br>Setting: Primary care<br><br>Mean age: 38.7 (18-79)<br><br>Female: 61.7%	N = 180<br><br>16.6% MDD	Administration: Self-report<br><br>Language: Malay<br><br>Cut-offs: 5 to 12	DSM-IV CIDI	b) No COI declaration<br>c) Funding acknowledged (academic /health research institutions)
4. Chagas et al. (2013)	Country: Brazil	N = 84	Administration: self-report	DSM-IV SCID	a) COI declaration “None declared”

For peer review only

For peer review only - http://bmjopen.bmj.com/site/about/guidelines.xhtml

	Setting: Secondary care<br><br>Mean age: Not stated<br><br>Female: 52.7%	25.5% MDD	Language: Brazilian<br><br>Cut-offs: 7 to 10		b) Funding acknowledged (academic/health research institutions)
6. de Lima Osorio et al. (2009)	Country: Brazil<br><br>Setting: Primary care<br><br>Mean age: Unclear<br><br>Female: 100%	N = 177<br><br>34% MDD	Administration: research assistants<br><br>Language: Brazilian Portuguese<br><br>Cut-offs: 10 to 15	DSM-IV SCID	a) No COI declaration<br>b) Funding acknowledged (academic institutions)
7. Elderon et al. (2011)	Country: USA<br><br>Setting: Secondary care<br><br>Mean age: Unclear<br><br>Female: 18%	N = 1022<br><br>18.3% MDD	Administration: self-report<br><br>Language: English<br><br>Cut-offs: 10	C-DIS	a) COI declaration – 'No disclosures'<br>b) Funding acknowledged (academic institutions and industry – AHA Pharmaceuticals Roundtable) – 'The funding organisations had no role in the design or conduct of the study, collection, management, analysis or interpretation of data; or preparation, review or approval of the manuscript.'
8. Fann et al. (2005)	Country: US<br><br>Setting: Trauma hospital (inpatients with traumatic	N = 135<br><br>16.3% MDD	Administration: Telephone-administered<br><br>Language: English	DSM-IV SCID	b) No COI declaration<br>c) Funding acknowledged (academic

For peer review only - http://bmjopen.bmj.com/site/about/guidelines.xhtml

	brain injury)<br><br>Mean age: 42 (SD=17.9)<br><br>Female: 29.1%		Cut-offs: 10		institutions)
9. Fine et al. (2013)	Country: USA<br><br>Setting: Primary care (Ohio Army National Guard)<br><br>Mean age: 31 (17-60)<br><br>Female: 12%	N = 498<br><br>21.5% MDD	Administration: Telephone-administered<br><br>Language: English<br><br>Cut-offs: 10,15	DSM-IV SCID-I	a) COI – last author disclosed financial and consulting interests (Pfizer not one of them). All other authors declared that they have no COI.<br>b) Funding acknowledged – DoD Medical Research. ‘’The sponsor had no role in study design, data collection, analysis, interpretation of results, report writing or manuscript submission.
10. Gelaye et al. (2013)	Country: Ethiopia<br><br>Setting: General hospital<br><br>Mean age: 34.9 (SD=11.6)<br><br>Female: 63.1 %	N = 363<br><br>12.6% MDD	Administration: Researcher-administered<br><br>Language: Amharic<br><br>Cut-offs: 9 to 11	DSM-IV SCAN	c) No COI declaration<br>d) Funding acknowledged (academic /health research institutions)
11. Gilbody et al.	Country: UK	N = 96	Administration: Not	DSM-IV	a) COI declaration –

For peer review only

(2007)	Setting: Primary care<br><br>Mean age: 42.5 (SD 13.6)<br><br>Female: 77%	37.5 MDD	stated<br><br>Language: English<br><br>Cut-offs: 9 to 13	SCID	last author involved in the development of one of the instruments (CORE-OM), 'but does not gain financially from its use.<br>b) Funding acknowledged (academic /health research institutions)
12. Gjerdingen et al. (2009)	Country: USA<br><br>Setting: Community<br><br>Mean age: 29.3<br><br>Female: 100%	N = 438<br><br>4.6% MDD	Administration: Telephone or self-report<br><br>Language: English<br><br>Cut-offs: 10	DSM-IV SCID	c) No COI declaration<br>d) Funding acknowledged (academic /health research institutions)
14. Hyphantis et al. (2011)	Country: Greece<br><br>Setting: Hospital – rheumatology patients<br><br>Mean age: 54.2 (SD = 13.5)<br><br>Female: 74%	N = 213<br><br>32.4% MDD	Administration: Researcher administered<br><br>Language: Greek<br><br>Cut-offs: 4 to 16	DSM-IV MINI	c) No COI declaration<br>d) No funding acknowledgement
15. Khamseh et al. (2011)	Country: Iran<br><br>Setting: Outpatient diabetic clinic<br><br>Mean age: 56.1 (SD=9.6)	N = 185<br><br>43.2% MDD	Administration: Self-report<br><br>Language: Persian<br><br>Cut-offs: 10,13	DSM-IV SCID	c) COI declaration: The authors declared no competing interests<br>d) Funding acknowledged (academic /health

For peer review only - http://bmjopen.bmj.com/site/about/guidelines.xhtml

	Female: 51.8%				research institutions)
19. Liu et al. (2011)	Country: Taiwan<br><br>Setting: Primary care<br><br>Mean age: Not specified<br><br>Female: 60.9%	N = 1532<br><br>3.3% MDD	Administration: Self-report<br><br>Language: Chinese version<br><br>Cut-offs: 9 to 11	SCAN	a) a) No COI declaration<br>b) Funding acknowledged (academic /health research institutions)
20. Lotrakul et al. (2008)	Country: Thailand<br><br>Setting: Primary care<br><br>Mean age: 45.0 (SD = 14.30)<br><br>Female: 73.7%	N = 279<br><br>6.8% MDD	Administration: Self report<br><br>Language: Thai<br><br>Cut-offs: 7 to 15	DSM-IV MINI	c) No COI declaration<br>d) Funding acknowledged (academic /health research institutions)
23. Patel et al. (2008)	Country: India<br><br>Setting: Primary care<br><br>Mean age: 37.5 (18-83)<br><br>Female: 56.4%	N = 299<br><br>4.3% MDD	Administration: Face-to-face interview<br><br>Language: Not specified<br><br>Cut-offs: 7 to 15	CIS-R	a) COI declaration – No Declaration of Interest<br>b) Funding acknowledged (academic /health research institutions)
24. Phelan et al. (2010)	Country: USA<br><br>Setting: Primary care (elderly)<br><br>Mean age: 78 (SD=7)<br><br>Female: 62%	N = 71<br><br>12% MDD	Administration: Research assistant<br><br>Language: English<br><br>Cut-offs: 8 to 12	DSM-IV SCID	a) COI declaration – No competing interests<br>b) Funding acknowledged (academic /health research institutions). 'The funder had no role in the study design, methods,

For peer review only - http://bmjopen.bmj.com/site/about/guidelines.xhtml

					data collection, analysis or interpretation of data, nor any role in the preparation of the manuscript or decision to submit the manuscript for publication.
25. Rooney et al. (2013)	Country: UK<br><br>Setting: Secondary care (glioma)<br><br>Mean age: 54.2 (SD=12.3)<br><br>Female: 42.6%	N = 129<br><br>13.5% MDD	Administration: Self-report<br><br>Language: English<br><br>Cut-offs: 8 to 11	DSM-IV SCID	a) COI declaration "The authors declare that they have no COI"<br>b) Funding acknowledged (academic/health research institutions)
26. Sherina et al. (2012)	Country: Malaysia<br><br>Setting: Primary care<br><br>Mean age: 30.9 (18-81)<br><br>Female: 100%	N= 146<br><br>21.2% MDD	Administration: Self-report<br><br>Language: Malay<br><br>Cut-offs: 10	CIDI	a) COI declaration "The authors declare that they have no competing interests"<br>b) Funding acknowledged (academic/health research institutions)
27. Sidebottom et al. (2012)	Country: USA<br><br>Setting: Community (prenatal)<br><br>Mean age: 23 (SD=5.5)<br><br>Female: 100%	N = 745<br><br>3.6% MDD	Administration: Interview<br><br>Language: English<br><br>Cut-offs: 10	DSM-IV SCID	b) COI declaration "The authors declare that they have no financial COI"<br>b) Funding acknowledged (academic/health research institutions)
28. Stafford et al. (2007)	Country: Australia<br><br>Setting: Secondary care (cardiac procedures)	N = 193<br><br>18.1% MDD	Administration: Self-report<br><br>Language: English	DSM-IV MINI	b) No COI declaration<br>c) Funding acknowledged (academic/health

For peer review only - http://bmjopen.bmj.com/site/about/guidelines.xhtml

	Mean age: 64.14 (38-91)<br><br>Female: 19.2%		Cut-offs: 10		research institutions)
30. Thombs et al. (2008)	Country: US<br><br>Setting: Hospital (outpatients with coronary heart disease)<br><br>Mean age: 67 (SD = 11)<br><br>Female: 18%	N = 1024<br><br>22% MDD	Administration: Not stated<br><br>Language: English<br><br>Cut-offs: 7 to 10	DSM C-DIS	b) COI declaration "None disclosed"<br>b) Funding acknowledged (academic/health research institutions)
32. Watnick et al. (2005)	Country: USA<br><br>Setting: Secondary care (dialysis)<br><br>Mean age: 63 (SD=15)<br><br>Female: 32.3%	N = 62<br><br>19% MDD	Administration: Self-report<br><br>Language: English<br><br>Cut-offs: 10	DSM-IV SCID	b) No COI declaration<br>c) Funding acknowledged (academic/health research institutions)
34. Wittkampf et al. (2009)	Country: Netherlands<br><br>Setting: Primary care<br><br>Mean age: 49.8<br><br>Female: 66.7%	N = 664<br><br>12.3% MDD	Administration: Self-report<br><br>Language: Not specified<br><br>Cut-offs: 10 and 15	DSM-IV SCIDI	No COI declaration<br>b) Funding acknowledged (academic/health research institutions)
35. Zhang et al. (2013)	Country: Hong Kong<br><br>Setting: Secondary care (diabetic outpatients)<br><br>Mean age: 55.1 (SD=9.5)	N = 99<br><br>23.2% MDD	Administration: Self-report<br><br>Language: Chinese version<br><br>Cut-offs: 15	DSM-IV MINI	COI declaration – last author acknowledged financial COI. The other authors declare that they have no competing interests.<br>) Funding acknowledged

For peer review only - http://bmjopen.bmj.com/site/about/guidelines.xhtml

	Female: 40.8%				(academic/health research institutions)
36. Zuithoff et al. (2010)	Country: Netherlands<br><br>Setting: Primary care<br><br>Age (yrs): M = 51 (sd = 16.7)<br><br>Female: 63%	N = 1338<br><br>Depressed: 13%	Administration: Self-report<br><br>Language: Dutch	DSM-IV CIDI	b) COI declaration "The authors declare that they have no competing interests.<br>b) Funding acknowledged (academic/health research institutions)

Table 3: Quality assessment of included studies in the algorithm meta-analysis (Manea et al., 2014)

Study	Patient selection: Consecutive or random sample	Patient selection: Avoid case-control / avoid artificially inflated base rate	Patient selection: Avoided inappropriate exclusions	Patient selection: Overall risk of bias	Index test: PHQ-9 interpreted blind to reference test	Index test: If translated, appropriate translation	Index test: If translated, psychometric properties reported	Index test: Overall risk of bias
**Allegiant studies**								
Diez-Quevedo et al. (2001)	×	✓	×	High	?	✓	✓	Unclear
Gräfe et al. (2004)	✓	✓	✓	Low	?	✓	✓	Unclear

For peer review only - http://bmjopen.bmj.com/site/about/guidelines.xhtml

Lowe et al. (2004)	×	✓	✓	High	✓	✓	✓	Low
Muramatsu et al. (2007)	?	✓	?	Unclear	✓	✓	?	Unclear
Navines et al. (2012)	✓	✓	✓	Low	✓	✓	?	Unclear
Spitzer et al. (1999)	×	✓	✓	High	✓	n/a	n/a	Low
Thekkumpurath et al. (2010)	×	×	✓	High	✓	n/a	n/a	Low
**Non-allegiant studies**								
Arroll et al. (2010)	✓	✓	✓	Low	✓	n/a	n/a	Low
Ayalon et al. (2010)	?	✓	✓	Unclear	?	✓	?	Unclear
Eack et al. (2006)	?	✓	?	Unclear	?	n/a	n/a	Unclear
Fann et al. (2005)	✓	×	×	High	✓	n/a	n/a	Low
Gelaye et al. (2013)	?	×	?	High	✓	✓	?	Unclear
Gjerdingen et	✓	✓	✓	Low	?	n/a	n/a	Unclear

For peer review only - http://bmjopen.bmj.com/site/about/guidelines.xhtml

al. (2009)								
Henkel et al. (2004)	✓	✓	✓	Low	?	n/a	n/a	Unclear
Hyphantis et al. (2011)	✓	✓	×	High	✓	?	?	Unclear
Inagaki et al. (2013)	✓	×	✓	High	✓	?	?	Unclear
Khamseh et al. (2011)	✓	✓	?	Unclear	✓	✓	?	Unclear
Lamers et al (2008)	✓	×	×	High	✓	?	?	Unclear
Lotrakul et al. (2008)	×	✓	?	High	✓	✓	?	Unclear
Persoons et al. (2003)	✓	✓	✓	Low	✓	✓	n/a	Low
Picardi et al. (2005)	✓	✓	✓	Low	✓	?	?	Unclear
Stafford et al. (2007)	✓	✓	✓	Low	✓	n/a	n/a	Low
Thombs et al. (2008)	×	✓	?	Unclear	?	n/a	n/a	Unclear
Thomspon et	?	✓	✓	Unclear	?	n/a	n/a	Unclear

For peer review only

For peer review only - http://bmjopen.bmj.com/site/about/guidelines.xhtml

al. (2011)								
Turner et al. (2012)	✓	✓	✓	Low	✓	n/a	n/a	Low
Van Steenbergen-Wijenburg (2010)	?	✓	✓	Unclear	?	?	?	Unclear
Zuithoff et al. (2010)	✓	✓	✓	Low	✓	✓	?	Unclear

✓ = criterion met; × = criterion not met; ? = insufficient information to code whether criterion met; n/a = not applicable

Table 3: Quality assessment of included studies in the algorithm meta-analysis (Manea et al., 2014) (continued)

Study	**Reference test:** Reference test correctly classifies target condition	**Reference test:** Reference test interpreted blind to PHQ-9	**Reference test:** If translated, appropriate translation	**Reference test:** If translated, psychometric properties reported	**Reference test:** Overall risk of bias	**Flow / timing:** Interval of two weeks or less	**Flow / timing:** All participants receive same reference test	**Flow / timing:** All participants included in analysis?	**Flow / timing:** Overall risk of bias
**Allegiant studies**									
Diez-Quevedo et al. (2001)	✓	✓	✓	?	Unclear	✓	✓	✓	Low

For peer review only

For peer review only - http://bmjopen.bmj.com/site/about/guidelines.xhtml

Gräfe et al. (2004)	✓	?	n/a	n/a	Unclear	✓	✓	✓	Low
Lowe et al. (2004)	✓	✓	n/a	n/a	Low	✓	✓	✓	Low
Muramatsu et al. (2007)	✓	✓	✓	✓	Low	✓	✓	?	Unclear
Navines et al. (2012)	✓	✓	?	?	Unclear	✓	✓	✓	Low
Spitzer et al. (1999)	✓	✓	n/a	n/a	Low	✓	✓	×	High
Thekkumpurath et al. (2010)	✓	✓	n/a	n/a	Low	?	✓	×	High
**Non-allegiant studies**									
Arroll et al. (2010)	✓	✓	n/a	n/a	Low	✓	✓	✓	Low
Ayalon et al. (2010)	✓	?	✓	?	Unclear	?	✓	✓	Unclear
Eack et al. (2006)	✓	?	n/a	n/a	Unclear	?	✓	?	Unclear
Fann et al. (2005)	✓	?	n/a	n/a	Unclear	✓	✓	×	High
Gelaye et al.	✓	✓	✓	✓	Low	✓	✓	×	High

For peer review only

(2013)									
Gjerdingen et al. (2009)	✓	?	n/a	n/a	Unclear	✓	✓	×	High
Henkel et al. (2004)	✓	?	n/a	n/a	Unclear	✓	✓	×	High
Hyphantis et al. (2011)	✓	✓	?	?	Unclear	✓	✓	×	High
Inagaki et el. (2013)	✓	✓	✓	?	Unclear	✓	✓	×	High
Khamseh et al (2011)	✓	✓	✓	?	Unclear	✓	✓	?	Unclear
Lamers et al. (2008)	✓	✓	?	?	Unclear	?	✓	×	High
Lotrakul et al. (2008)	✓	✓	✓	✓	Low	?	✓	×	High
Persoons et al. (2003)	✓	✓	?	?	Unclear	✓	✓	✓	Low
Picardi et al. (2005)	✓	✓	✓	?	Unclear	✓	✓	×	High
Stafford et al. (2007)	✓	✓	n/a	n/a	Low	✓	✓	×	High
Thombs et al.	?	✓	n/a	n/a	Unclear	✓	✓	✓	Low

For peer review only

For peer review only - http://bmjopen.bmj.com/site/about/guidelines.xhtml

Table 4: Quality assessment of included studies in the summed item scoring method cut-off point 10 meta-analysis (Moriarty et al., 2015)

(2008)									
Thompson et al. (2011)	✓	?	n/a	n/a	Unclear	✓	✓	✗	High
Turner et al. (2012)	✓	?	n/a	n/a	Unclear	?	✓	✗	High
Van Steenbergen-Wijenburg (2010)	✓	✗	?	?	High	✓	✓	✗	High
Zuithoff et al. (2010)	✓	✓	?	?	Unclear	?	✓	✓	Unclear

✓ = criterion met; ✗ = criterion not met; ? = insufficient information to code whether criterion met; n/a = not applicable

For peer review only

Study	Patient selection: Consecutive or random sample	Patient selection: Avoid case-control / avoid artificially inflated base rate	Patient selection: Avoided inappropriate exclusions	Patient selection: Overall risk of bias	Index test: PHQ-9 interpreted blind to reference test	Index test: Was a threshold pre-specified?	Index test: If translated, appropriate translation	Index test: If translated, psychometric properties reported	Index test: Overall risk of bias
**Allegiant studies**									
13. Gräfe et al. (2004)	✓	✓	✓	Low	?	✓	✓	✓	Unclear
16. Kroenke et al. (2011)	✓	✓	✓	Low	✓	✓	n/a	n/a	Low
22. Navinés et al. (2012)	✓	✓	✓	Low	✓	✓	✓	?	Unclear
29. Thekkumpurath et al. (2010)	✗	✗	✓	High	✓	✓	n/a	n/a	Low
33. Williams et al. (2005)	✓	✓	✓	Low	?	✓	n/a	n/a	Unclear
**Non-allegiant studies**									
1. Adewuya et	✓	✓	✗	Unclear	✓	✓	n/a	n/a	Low

For peer review only

For peer review only - http://bmjopen.bmj.com/site/about/guidelines.xhtml

al. (2006)									
2. Arroll et al. (2010)	✓	✓	✓	Low	✓	✓	n/a	n/a	Low
3. Azah et al. (2005)	✓	✗	?	High	✓	✓	✓	✓	Low
4. Chagas et al. (2013)	✓	✓	✓	Low	✓	✓	✓	✓	Low
6. de Lima Osorio et al. (2009)	✓	✗	✓	High	?	✗	n/a	n/a	High
7. Elderon et al. (2011)	✓	✓	✓	Low	✓	✓	n/a	n/a	Low
8. Fann et al. (2005)	✓	✗	✗	High	✓	✓	n/a	n/a	Low
9. Fine et al. (2013)	✓	✓	✓	Low	?	✓	n/a	n/a	Unclear
10. Gelaye et al. (2013)	?	✗	?	High	✓	✗	✓	?	High
11. Gilbody et al.	?	✓	?	Unclear	✓	✓	n/a	n/a	Low

For peer review only - http://bmjopen.bmj.com/site/about/guidelines.xhtml

(2007)									
12. Gjerdingen et al. (2009)	✓	✓	✓	Low	?	✓	n/a	n/a	Unclear
14. Hyphantis et al. (2011)	✓	✗	✓	High	✓	✓	?	?	Unclear
15. Khamseh et al. (2011)	✓	✓	?	Unclear	✓	✓	✓	?	Unclear
19. Liu et al. (2011)	✓	✓	?	Unclear	✓	✗	✓	?	High
20. Lotrakul et al. (2008)	✗	✓	?	Unclear	✓	✓	✓	?	Unclear
23. Patel et al. (2008)	✓	✓	✓	Low	✓	✓	?	?	Unclear
24. Phelan et al. (2010)	✗	✓	✓	High	✓	✗	n/a	n/a	High
25. Rooney et al. (2013)	✓	✓	✓	Low	?	✗	n/a	n/a	High
26. Sherina et al.	✓	✓	✗	High	✓	✓	✓	✓	Low

For peer review only - http://bmjopen.bmj.com/site/about/guidelines.xhtml

For peer review only

(2012)									
27. Sidebottom et al. (2012)	✓	✓	✓	Low	✓	✓	n/a	n/a	Low
28. Stafford et al. (2007)	✓	✓	✓	Low	✓	✓	n/a	n/a	Low
30. Thombs et al. (2008)	✗	✓	?	High	✓	?	n/a	n/a	Unclear
32. Watnick et al. (2005)	?	✗	✓	High	✓	✓	n/a	n/a	Low
34. Wittkampf et al. (2009)	✓	✓	✓	Low	✓	?	n/a	n/a	Unclear
35. Zhang et al. (2013)	✓	✓	?	Unclear	?	✓	?	?	Unclear
36. Zuithoff et al. (2010)	✓	✓	✓	Low	✓	✓	✓	?	Unclear

Table 4: Quality assessment of included studies in the summed item scoring method cut-off point 10 meta-analysis (Moriarty et al., 2015) (continued)

Study	Reference test: Reference test	Reference test: Reference test	Reference test: If translated,	Reference test: If translated, psychometric	Reference test: Overall risk of	Flow / timing: Interval of two	Flow / timing: All participants	Flow / timing: All participants	Flow / timing: Overall risk of

For peer review only - http://bmjopen.bmj.com/site/about/guidelines.xhtml

For peer review only

	correctly classifies target condition	interpreted blind to PHQ-9	appropriate translation	properties reported	bias	weeks or less	receive same reference test	included in analysis?	bias
**Allegiant studies**									
13. Gräfe et al. (2004)	✓	?	n/a	n/a	Unclear	✓	✓	✓	Low
16. Kroenke et al. (2011)	✓	✓	n/a	n/a	Low	✓	✓	✓	Low
22. Navinés et al. (2012)	✓	✓	?	?	Unclear	✓	✓	✓	Low
29. Thekkumpurath et al. (2010)	✓	✓	n/a	n/a	Low	?	✓	✓	Unclear
33. Williams et al. (2005)	✓	?	n/a	n/a	Unclear	?	✓	✓	Unclear
Non-allegiant studies									
1. Adewuya et al. (2006)	✓	✓	n/a	n/a	Low	✓	✓	✓	Low
2. Arroll et al.	✓	✓	n/a	n/a	Low	?	✓	✓	Unclear

For peer review only - http://bmjopen.bmj.com/site/about/guidelines.xhtml

(2010)									
3. Azah et al. (2005)	✓	✓	✓	✓	Low	✓	✓	✗	High
4. Chagas et al. (2013)	✓	✓	?	?	Unclear	✓	✓	✗	High
6. de Lima Osorio et al. (2009)	✓	?	n/a	n/a	Unclear	?	✓	✓	Unclear
7. Elderon et al. (2011)	✓	✓	n/a	n/a	Low	✓	✓	✓	Low
8. Fann et al. (2005)	✓	?	n/a	n/a	Unclear	✓	✓	✗	High
9. Fine et al. (2013)	✓	?	n/a	n/a	Unclear	?	✓	✓	Unclear
10. Gelaye et al. (2013)	✓	✓	✓	✓	Low	✓	✓	✗	High
11. Gilbody et al. (2007)	✓	✓	n/a	n/a	Low	?	✓	✓	Unclear
12. Gjerdingen	✓	?	n/a	n/a	Unclear	✓	✓	✗	High

For peer review only

et al. (2009)									
14. Hyphantis et al. (2011)	✓	✓	?	?	Unclear	✓	✓	✗	High
15. Khamseh et al. (2011)	✓	✓	✓	?	Unclear	✓	✓	?	Unclear
19. Liu et al. (2011)	✓	✓	✓	✓	Low	✓	✓	?	Unclear
20. Lotrakul et al. (2008)	✓	✓	✓	✓	Low	?	✓	✗	High
23. Patel et al. (2008)	✓	✓	✓	?	Unclear	?	✓	✗	High
24. Phelan et al. (2010)	✓	✓	n/a	n/a	Low	✓	✓	✓	Low
25. Rooney et al. (2013)	✓	?	n/a	n/a	Unclear	?	✓	✗	High
26. Sherina et al. (2012)	✓	✓	✓	✓	Low	✓	✓	✓	Low
27. Sidebottom	✓	✓	n/a	n/a	Low	✓	✓	✗	High

For peer review only

et al. (2012)									
28. Stafford et al. (2007)	✓	✓	n/a	n/a	Low	✓	✓	✗	High
30. Thombs et al. (2008)	?	✓	n/a	n/a	Unclear	✓	✓	✓	Low
32. Watnick et al. (2005)	✓	✓	n/a	n/a	Low	✓	✓	✓	Low
34. Wittkampf et al. (2009)	✓	✓	n/a	n/a	Low	?	✓	✗	High
35. Zhang et al. (2013)	✓	?	✓	✓	Unclear	✗	✓	✗	High
36. Zuithoff et al. (2010)	✓	✓	?	?	Unclear	?	✓	✓	Unclear

For peer review only

Table 5. Pooled estimates of diagnostic properties of the PHQ-9 at cut-off point 10 and using algorithm scoring method in the non-independent vs independent studies groups

Settings	No of studies	No of patients	Sensitivity (95% CI)	Specificity (95% CI)	Pooled positive likelihood ratio (95% CI)	Pooled negative likelihood ratio (95% CI)	Diagnostic odds ratio (95% CI)	Heterogeneity: I^2

**Manea et al, 2014 SR – RA group**	7	4,065	0.77 (0.70 – 0.84)	0.94 (0.90 – 0.97)	14.97 (8.39 – 26.71)	0.23 (0.17 - 0.31)	64.40 (34.15 – 121.43)	78.9%
**Manea et al, 2014 SR Independent studies**	21	9,900	0.48 (0.41 – 0.91)	0.94 (0.91 – 0.95)	8.26 (6.15 – 11.09)	0.54 (0.48 – 0.62)	15.05 (11.03 – 20.52)	68.1%
**Moriarty et al., 2015 SR – RA group**	5	6,188	0.87 (0.77 – 0.93)	0.87 (0.76 – 0.94)	7.24 (3.74 – 14.03)	0.14 (0.08 - 0.25)	49.31 (25.74 – 94.48)	55.1%
**Moriarty et al., 2015 SR Independent studies**	26	13,164	0.76 (0.67 – 0.83)	0.88 (0.85 – 0.91)	6.72 (5.06 – 8.92)	0.26 (0.19 - 0.37)	24.96 (14.81 – 42.08)	81.5%

578

579

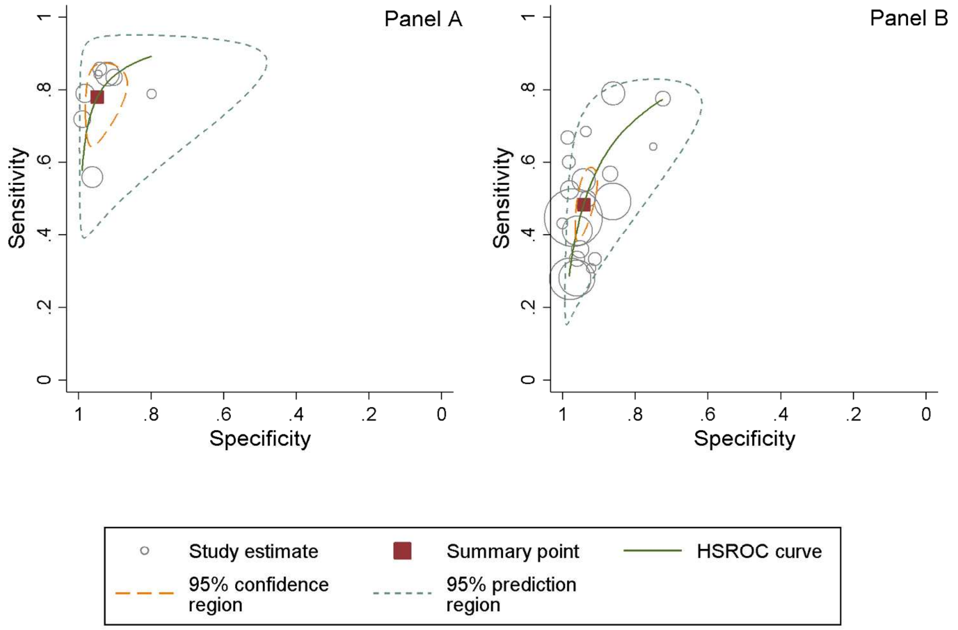

Figure 1. PHQ-9 algorithm scoring method summary ROC plot for the diagnosis of major depressive disorder in allegiant studies (Panel A) and non-allegiant studies (Panel B). Pooled sensitivity and specificity estimates using a bi-variate meta-analysis (HSROC hierarchical receiver-operating characteristic).

169x123mm (300 x 300 DPI)

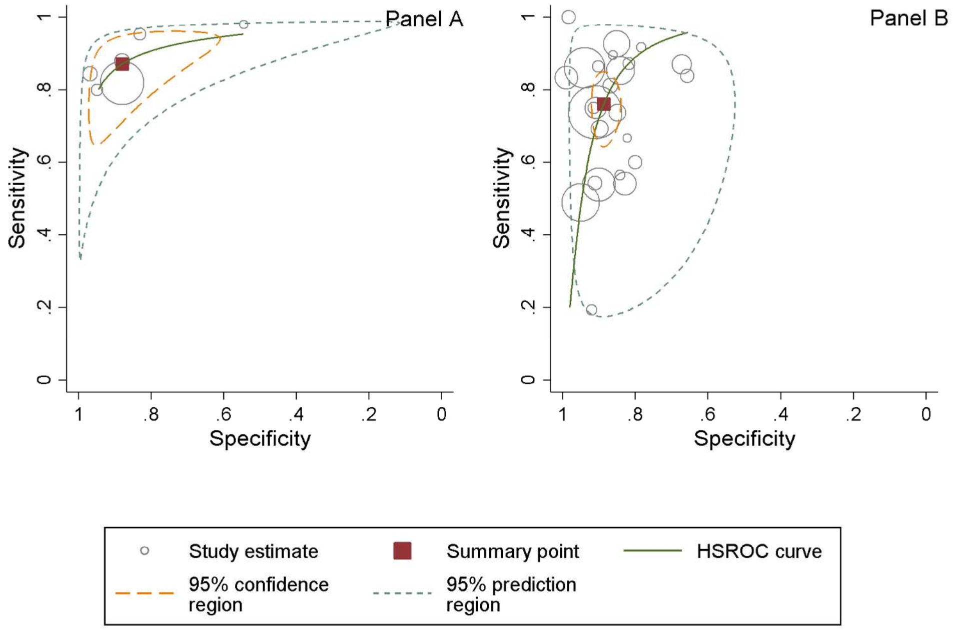

Caption : Figure 2. PHQ-9 summed items scoring method at cut-off point 10 summary ROC plot for diagnosis of major depressive disorder in allegiant studies (panel A) and non-allegiant studies (panel B). Pooled sensitivity and specificity using a bi-variate meta-analysis (HSROC hierarchical receiver-operating characteristic).

169x123mm (300 x 300 DPI)

For peer review only - http://bmjopen.bmj.com/site/about/guidelines.xhtml

Appendices to: Manea L, Boehnke JR, Gilbody S, Moriarty AS, McMillan D, Are there researcher allegiance effects in diagnostic validation studies of the PHQ-9? A systematic review and meta-analysis. Manuscript submitted for publication at BMJOpen.

Appendix 1

Figure 1: PRISMA flowchart - search and selection of included diagnostic accuracy studies for the systematic review of studies reporting diagnostic accuracy of the PHQ-9 at using the summed items scoring method (Manea et al, 2014)

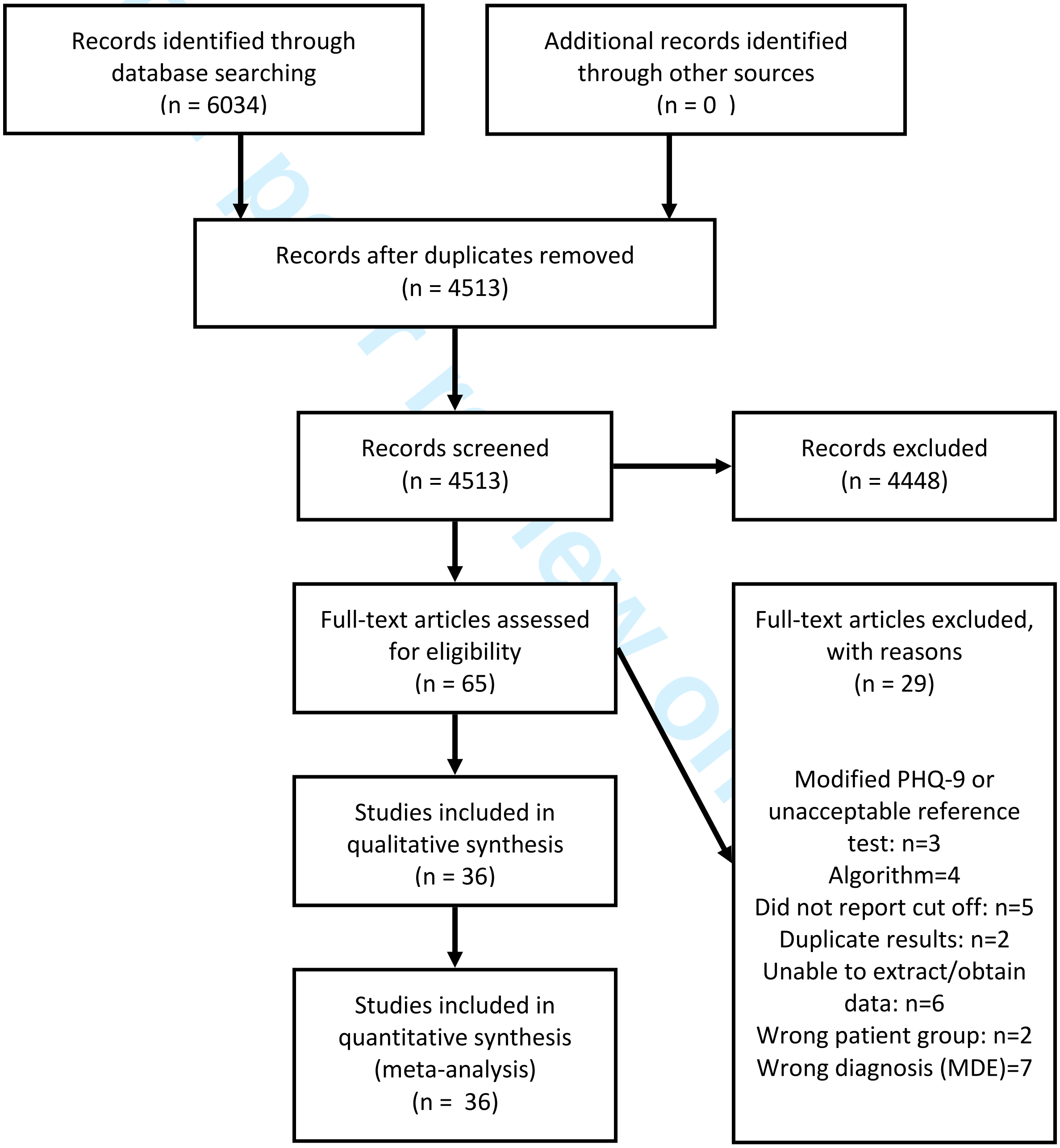

Figure 2: PRISMA flowchart - search and selection of included diagnostic accuracy studies for the systematic review of studies reporting diagnostic accuracy of the PHQ-9 at using the algorithm scoring method (Moriarty et al., 2015)

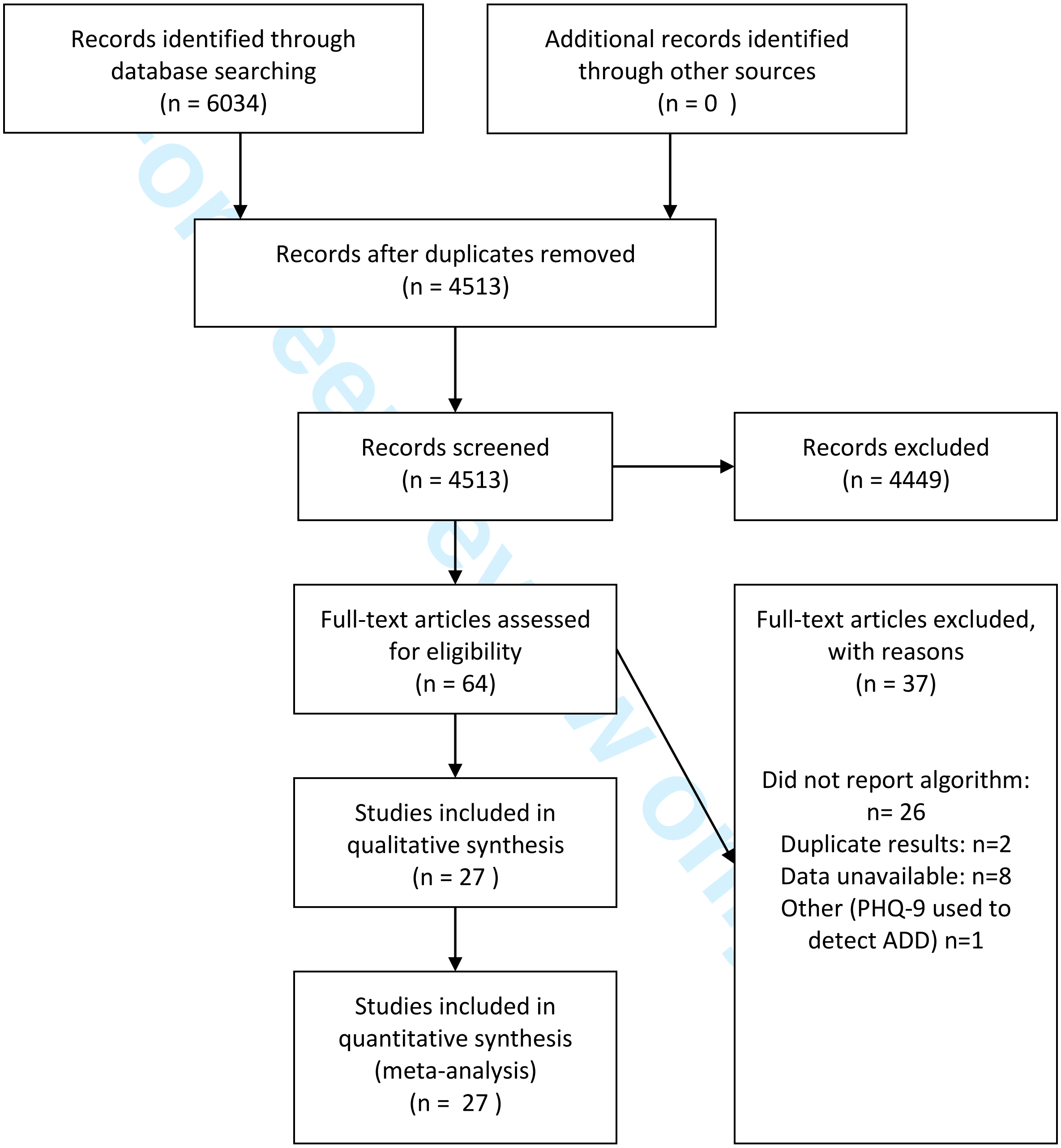

For peer review only - http://bmjopen.bmj.com/site/about/guidelines.xhtml

Appendix 2: Search terms used in Embase, MEDLINE and PsycINFO

(phq adj5 “9”).ti,ab.
(phq adj5 item$).ti,ab.
(patient health questionnaire adj5 “9”).ti,ab.
(patient health questionnaire adj5 item$).ti,ab.
(prime md adj5 “9”).ti,ab.
(prime md adj5 item$).ti,ab.

For peer review only

PRISMA 2009 Checklist

Section/topic	#	Checklist item	Reported on page #
**TITLE**			
Title	1	Identify the report as a systematic review, meta-analysis, or both.	1
**ABSTRACT**			
Structured summary	2	Provide a structured summary including, as applicable: background; objectives; data sources; study eligibility criteria, participants, and interventions; study appraisal and synthesis methods; results; limitations; conclusions and implications of key findings; systematic review registration number.	2
**INTRODUCTION**			
Rationale	3	Describe the rationale for the review in the context of what is already known.	4-5
Objectives	4	Provide an explicit statement of questions being addressed with reference to participants, interventions, comparisons, outcomes, and study design (PICOS).	5
**METHODS**			
Protocol and registration	5	Indicate if a review protocol exists, if and where it can be accessed (e.g., Web address), and, if available, provide registration information including registration number.	No
Eligibility criteria	6	Specify study characteristics (e.g., PICOS, length of follow-up) and report characteristics (e.g., years considered, language, publication status) used as criteria for eligibility, giving rationale.	5
Information sources	7	Describe all information sources (e.g., databases with dates of coverage, contact with study authors to identify additional studies) in the search and date last searched.	5
Search	8	Present full electronic search strategy for at least one database, including any limits used, such that it could be repeated.	Available online (see Manea et al., 2015; Moriarty et al., 2015)
Study selection	9	State the process for selecting studies (i.e., screening, eligibility, included in systematic review, and, if applicable, included in the meta-analysis).	5
Data collection process	10	Describe method of data extraction from reports (e.g., piloted forms, independently, in duplicate) and any processes for obtaining and confirming data from investigators.	6
Data items	11	List and define all variables for which data were sought (e.g., PICOS, funding sources) and any assumptions and simplifications made.	5-6
Risk of bias in individual studies	12	Describe methods used for assessing risk of bias of individual studies (including specification of whether this was done at the study or outcome level), and how this information is to be used in any data synthesis.	6

For peer review only

PRISMA 2009 Checklist

Summary measures	13	State the principal summary measures (e.g., risk ratio, difference in means).	6
Synthesis of results	14	Describe the methods of handling data and combining results of studies, if done, including measures of consistency (e.g., I^2) for each meta-analysis.	6

Section/topic	#	Checklist item	Reported on page #
Risk of bias across studies	15	Specify any assessment of risk of bias that may affect the cumulative evidence (e.g., publication bias, selective reporting within studies).	6, 21
Additional analyses	16	Describe methods of additional analyses (e.g., sensitivity or subgroup analyses, meta-regression), if done, indicating which were pre-specified.	6
**RESULTS**			
Study selection	17	Give numbers of studies screened, assessed for eligibility, and included in the review, with reasons for exclusions at each stage, ideally with a flow diagram.	Appendix
Study characteristics	18	For each study, present characteristics for which data were extracted (e.g., study size, PICOS, follow-up period) and provide the citations.	Tables 1 and 2
Risk of bias within studies	19	Present data on risk of bias of each study and, if available, any outcome level assessment (see item 12).	Tables 3 and 4
Results of individual studies	20	For all outcomes considered (benefits or harms), present, for each study: (a) simple summary data for each intervention group (b) effect estimates and confidence intervals, ideally with a forest plot.	N/A
Synthesis of results	21	Present results of each meta-analysis done, including confidence intervals and measures of consistency.	Table 5
Risk of bias across studies	22	Present results of any assessment of risk of bias across studies (see Item 15).	Tables 3 and 4
Additional analysis	23	Give results of additional analyses, if done (e.g., sensitivity or subgroup analyses, meta-regression [see Item 16]).	11 and 17
**DISCUSSION**			
Summary of evidence	24	Summarize the main findings including the strength of evidence for each main outcome; consider their relevance to key groups (e.g., healthcare providers, users, and policy makers).	17-21
Limitations	25	Discuss limitations at study and outcome level (e.g., risk of bias), and at review-level (e.g., incomplete retrieval of identified research, reporting bias).	21
Conclusions	26	Provide a general interpretation of the results in the context of other evidence, and implications for future research.	21-22
**FUNDING**			

For peer review only

PRISMA 2009 Checklist

Funding	27	Describe sources of funding for the systematic review and other support (e.g., supply of data); role of funders for the systematic review.	23

From: Moher D, Liberati A, Tetzlaff J, Altman DG, The PRISMA Group (2009). Preferred Reporting Items for Systematic Reviews and Meta-Analyses: The PRISMA Statement. PLoS Med 6(6): e1000097. doi:10.1371/journal.pmed1000097

For more information, visit: **www.prisma-statement.org**.

For peer review only

For peer review only - http://bmjopen.bmj.com/site/about/guidelines.xhtml

BMJ Open

Are there researcher allegiance effects in diagnostic validation studies of the PHQ-9? A systematic review and meta-analysis

Journal:	*BMJ Open*
Manuscript ID	bmjopen-2016-015247.R2
Article Type:	Research
Date Submitted by the Author:	21-Jul-2017
Complete List of Authors:	Manea, Laura; University of York, Health Sciences<br>Boehnke, Jan Rasmus; University of York<br>Gilbody, Simon; The University of York, Department of Health Sciences<br>Moriarty, Andrew; University of York, Health Sciences<br>McMillan, Dean; University of York, Department of Health Sciences
**Primary Subject Heading**:	Mental health
Secondary Subject Heading:	Diagnostics
Keywords:	Depression & mood disorders < PSYCHIATRY, Screening, PHQ-9, diagnostic meta-analysis, allegiance effect

SCHOLARONE™
Manuscripts

For peer review only

Are there researcher allegiance effects in diagnostic validation studies of the PHQ-9? A systematic review and meta-analysis

Laura Manea MMedSci MRCPsych*, Jan R. Boehnke PhD, Simon Gilbody DPhil FRCPsych FRSA, Andrew S. Moriarty MRes, Dean McMillan PhD

*Corresponding Author

Hull York Medical School and Department of Health Sciences, ARRC Building, University of York, YO10 5DD

Email: laura.manea@york.ac.uk

For peer review only

Abstract

Objectives To investigate whether an authorship effect is found that leads to better performance in studies conducted by the original developers of the PHQ-9 (allegiant studies).

Design Systematic review with random effects bivariate diagnostic meta-analysis. Search strategies included electronic databases, examination of reference lists, and forward citation searches.

Inclusion criteria Included studies provided sufficient data to calculate the diagnostic accuracy of the PHQ-9 against a gold standard diagnosis of major depression using the algorithm or the summed item scoring method at cut-off point 10.

Data extraction Descriptive information, methodological quality criteria, and 2×2 contingency tables.

Results

For peer review only - http://bmjopen.bmj.com/site/about/guidelines.xhtml

Seven allegiant and twenty independent studies reported the diagnostic performance of the PHQ-9 using the algorithm scoring method. Pooled diagnostic odds ratio (DOR) for the allegiant group was 64.40, and 15.05 for non-allegiant studies group. The allegiance status was a significant predictor of DOR variation ($p < 0.0001$).

Five allegiant studies and twenty-six non-allegiant studies reported the performance of the PHQ-9 at recommended cut-off point of 10. Pooled DOR for the allegiant group was 49.31, and 24.96 for the non-allegiant studies. The allegiance status was a significant predictor of DOR variation ($P = 0.015$).

Some potential alternative explanations for the observed authorship effect including differences in study characteristics and quality were found, though it is not clear how some of them account for the observed differences

Conclusions

Allegiant studies reported better performance of the PHQ-9. Allegiance status was predictive of variation in the DOR. Based on the observed differences between independent and non-independent studies we were unable to conclude or exclude that allegiance effects are present in studies examining the diagnostic performance of the PHQ-9. This study highlights the need for future meta-analyses of diagnostic validation studies of psychological measures to evaluate the impact of researcher allegiance in the primary studies.

Strengths and limitations of this study

For peer review only - http://bmjopen.bmj.com/site/about/guidelines.xhtml

a) An original study–the first meta-analysis of diagnostic validation studies of psychological measures to evaluate the impact of researcher allegiance.
b) Using rigorous methodology–strict inclusion/exclusion and quality assessment criteria.
c) We found that the allegiance effect was a significant predictor of the variation of the diagnostic odds ratio in the meta-regression analysis.
d) Substantial variability observed in methodological quality of included studies.
e) Based on the observed methodological differences between the independent and non-independent studies we were unable to conclude or exclude that allegiance effects are present in studies examining the diagnostic performance of the PHQ-9.

For peer review only

For peer review only - http://bmjopen.bmj.com/site/about/guidelines.xhtml

Research on allegiance effects has a long tradition in psychotherapy research. In this context *allegiance* describes the phenomenon that researchers and clinicians who developed a treatment approach or are for other reasons invested in it tend to find larger effect sizes in favour of their treatment than for comparison groups. [1] This finding has been extensively replicated [2], [3] and is also robust when the quality of research is controlled for. Researcher allegiance is subject of on-going debates about the design of efficacy studies as well as implications for policy. [2], [4], [5] Researcher allegiance is also discussed widely in the literature on experimental as well as evaluation research. [6] Since the motivational underpinnings of allegiance effects are potentially far more ingrained into human behaviour and decision making than previously thought (e.g., [7], they may occur commonly in clinical research in general.

Although it has been suggested that allegiance effects may play a role in the validation of psychological screening and case-finding tools (e.g., O'Shea et al., in press), systematic evaluations of this hypothesis are rare and studies that acknowledge potential allegiance effects in such studies mainly come from forensic psychology and psychiatry backgrounds. [8]–[11] Diagnostic validation studies are geared at establishing the sensitivity and specificity of a screening or case finding tool, which is used in practice to differentiate cases from non-cases or to decide about whether further assessment or treatment is indicated or will be offered. An allegiance effect in such studies would be seen in systematically higher sensitivities or specificities if the original author(s) is (are) part of the team of such a study. Such a bias would have a deleterious affect on practice through promising over-optimistic accuracy of the screening or case finding tool or in evaluating the cost-effectiveness of the measure in a screening or case-finding context.

The depression module of the Patient Health Questionnaire (PHQ-9) is a widely used depression-screening instrument in non-psychiatric settings. The PHQ-9 was developed by a team of researchers, with its development underwritten by an educational grant from Pfizer US Pharmaceuticals. [12] The PHQ-9 can be scored using different methods, including an algorithm based on DSM-IV criteria and a cut-off based on summed-item scores. The psychometric properties of these two approaches have been summarised in two recently published meta-analyses. [13], [14] The goal of the current review is to investigate, based on an established database of PHQ-9 diagnostic validation studies [13], [14],

For peer review only - http://bmjopen.bmj.com/site/about/guidelines.xhtml

whether an allegiance effect is found that leads to an increased sensitivity and specificity in studies that were conducted by researchers closely connected to the original developers of the instrument.

METHODS

Study Selection

Similar search strategies were used in both systematic reviews. (For full details please see Manea et al. (2014) and Moriarty et al. (2015)). Embase, MEDLine and PSYCHInfo were searched from 1999 (when the PHQ-9 was first developed) to August 2013 [13] and September 2013 [14] respectively, using the terms "PHQ-9", "PHQ", "PHQ$" and "patient health questionnaire". The search strategy is presented in Appendix 1. The reference lists of studies fitting the inclusion criteria were manually searched and a reverse citation search in Web of Science was performed. Authors of unpublished studies were contacted and conference abstracts were reviewed in an attempt to minimise publication bias.

The following inclusion-exclusion criteria were used:

Population: Adult population. *Instrument:* Studies that used the PHQ-9. *Comparison (reference standard):* The accuracy of the PHQ-9 had to be assessed against a recognised gold-standard instrument for the diagnosis of either Diagnostic and Statistical Manual (DSM) or International Classification of Disease (ICD) criteria for major depression. Studies were included if the diagnoses were made using a standardised diagnostic structured interview schedule (e.g. Mini International Neuropsychiatric Interview (MINI), Structured Clinical Interview for DSM Disorders (SCID)). Unguided clinician diagnoses with no reference to a standard structured diagnostic schedule or comparisons of the PHQ-9 with other self-report measures were excluded. Studies were also excluded if the target diagnosis was not major depressive disorder (MDD, e.g. any depressive disorder). *Outcome:* Studies had to report sufficient information to calculate a 2*2 contingency table for the algorithm or the recommended cut-off point 10. *Study design:* Any design. *Additional criterion:* We avoided double counting of evidence by ensuring that only

For peer review only - http://bmjopen.bmj.com/site/about/guidelines.xhtml

one study of those that reported overlapping datasets in different journals were included in the meta-analysis. Citations with overlapping samples were examined to establish whether they contained information relevant to the research question that was not contained in the included report.

Quality assessment

Quality assessment was performed using the QUADAS-2 tool, a tool for evaluating the risk of bias and applicability of primary diagnostic accuracy studies when conducting diagnostic systematic reviews. [15] It covers the areas of: patient selection, index test, reference standard and flow and timing. [16] This tool was adapted for the two reviews and quality assessments were carried out by two independent reviewers for all studies included in the reviews.

Data synthesis and statistical analysis

We constructed 2x2 tables for cut-off point 10 [14] and the algorithm scoring method [13] Pooled estimates of sensitivity, specificity, positive/negative likelihood ratios, and diagnostic odds ratios were calculated using random effects bivariate meta-analysis. [17] Heterogeneity was assessed using I^2 for the diagnostic odds ratio, an estimate of the proportion of study variability that is due to between-study variability rather than sampling error. We considered values of ≥50% to indicate substantial heterogeneity.[18] Summary Receiver Operator Characteristic curves (sROC) were constructed using the bivariate model to produce a 95% confidence ellipse within ROC space. [19] Each data point in the summary ROC space represents a separate study, unlike a traditional ROC plot, which explores the effect varying thresholds on sensitivity and specificity in a single study.

We undertook a meta-regression analysis of logit diagnostic odds ratio using research allegiance as covariate in the meta-regression model. [20], [21] Analyses were conducted using STATA version 12, with the metan, metandi and metareg user-written commands.

Allegiance Rating

For peer review only - http://bmjopen.bmj.com/site/about/guidelines.xhtml

We rated authorship on a paper if any of the developers of the PHQ-9 - Kurt Kroenke, MD, Robert L Spitzer, MD, and Janet B W Williams – as an indicator of potential allegiance. We also rated as evidence of allegiance as acknowledged collaborations with the developers of the PHQ-9, even if they were not listed as co-authors or if the authors acknowledged funding from Pfizer to conduct the study.

RESULTS

Overview of included studies

31 studies reported the diagnostic properties of the PHQ-9 at cut-off point 10 or above and were included in this analysis. [14] 27 studies were included in the algorithm review [13]. The study selection flowcharts can be found in Appendix 2 (figures 1 and 2). The characteristics of these studies are reported in tables 1 and 2 and the results of the methodological assessment are presented in tables 3 and 4.

Algorithm scoring method

Descriptive characteristics

The descriptive characteristics of the included studies are presented in table 1. Seven individual studies that reported the diagnostic performance of the PHQ-9 using the algorithm scoring method were co-authored by the original developers of the PHQ-9 [22]–[26], specifically acknowledged one of the developers and support by an educational grant from Pfizer US [27], or were co-authored by the first author of a

For peer review only - http://bmjopen.bmj.com/site/about/guidelines.xhtml

previous study that had also been co-authored by one of the developers [28]. Twenty non-allegiant studies reported the diagnostic properties of the PHQ-9 using the algorithm scoring method.

Three (43%, 3/7) of the allegiant studies were conducted exclusively in hospital settings [22], [26], [28]. The remaining four studies (67%, 4/7) were conducted in different settings or non-exclusively hospital settings: one in primary care [25] and three in mixed settings: psycho-somatic walk in clinics and family practices [23][1], outpatient clinics and family practices [24] and primary care and hospital settings [27]. In the non-allegiant group, thirteen (65%, 13/20) studies were conducted in hospital settings [29]–[41]. Of the remaining seven studies, six were conducted in primary care settings [42]–[47] and one in a community sample [48].

In both groups (non-allegiant and allegiant studies), the majority of studies validated a translated version of the PHQ-9. Two of the studies authored by developers (28%, 2/7) [25], [26], and eight (40%, 8/20) allegiant studies [29], [30], [37]–[40], [42], [48] were conducted in English.

The mean prevalence of major depressive disorder in the group of allegiant studies was 13.4 % (range 6.1% - 29.2%); in the non-allegiant group it was 15.5% (range 3.9% - 32.4%). The mean age of patients in the PHQ-9 developers group was 45.7; all but one study had a mean age in the range of 40 to 50 years. In the non-allegiant group the mean age was 54.6 (range 29.3 – 75.0), with almost half (8) of the studies reporting a mean age of over 60. The percentage of females in the PHQ-9 developers was 56.8% (range 28.6% - 67.8%) and in the non-allegiant group was 59.1 (18% -100%).

[1] This study provided separate estimates for the two settings in which it was conducted; therefore separate psychometric estimates were generated for each sample for both algorithm scoring method and summed items scoring method at cut-off point 10 (see below).

For peer review only - http://bmjopen.bmj.com/site/about/guidelines.xhtml

All allegiant studies used a self-reported PHQ-9, whereas in 7 non-allegiant studies (30%, 6/20) the PHQ-9 was administered by a researcher [30]–[33], [43], [48]. Apart from Muramatsu et al. (2007) all allegiant studies used the SCID as a gold standard; the non-allegiant studies used a wider range of gold standards including SCAN, CIDI, MINI, and C-DIS, though the SCID was also frequently used by the independent studies as well (45%, 9/20 studies).

Four out of the seven allegiant studies (57%) did not include a conflict of interests statement [22], [23], [25], [27]. Also, four (57%) of the allegiant studies acknowledged funding from Pfizer [23]–[25], [27]. Only one study [27] acknowledged the collaboration with one of the developers of the PHQ-9.

Of the non-allegiant studies, twelve (60%) did not include a conflict of interests statement [29]–[32], [35]–[37], [39], [44]–[46], [48]. It appears that newer studies were more likely to include a conflict of interest statement, which may reflect a recent change in reporting. Funding was acknowledged by most studies (18/20) and most received funding from academic or/and health research institutions. Two studies received funding from pharmaceutical companies – Lundbeck [43] and Pfizer [35] and one study acknowledged that Pfizer Italia provided the Italian version of PHQ-9 and gave the authors permission to use it [36].

Diagnostic test accuracy

Pooled sensitivity and specificity was calculated separately for the non-allegiant and allegiant studies. Pooled sensitivity for the allegiant studies of the PHQ-9 was 0.77 (95% CI = 0.70 – 0.84), pooled specificity was 0.94 (95% CI = 0.90 – 0.97), and the pooled diagnostic odds ratio was 64.40 (95% CI = 34.15 – 121.43). Heterogeneity was high (I^2 = 78.9%). Figure 1 represents the summary ROCs for this set of studies.

For peer review only - http://bmjopen.bmj.com/site/about/guidelines.xhtml

----

Figure 1. PHQ-9 algorithm scoring method summary ROC plot for the diagnosis of major depressive disorder in allegiant studies (Panel A) and non-allegiant studies (Panel B). Pooled sensitivity and specificity estimates using a bi-variate meta-analysis (*HSROC* hierarchical receiver-operating characteristic).

Pooled sensitivity for the non-allegiant studies was lower compared to the developer authored studies group at 0.48 (95% CI = 0.41 – 0.91), pooled specificity was the same at 0.94 (95% CI = 0.91 – 0.95). The pooled diagnostic odds ratio was approximately four times lower at 15.05 (95% CI = 11.03 – 20.52) (see figure 1). Heterogeneity was substantial at $I^2 = 68.1$ %.

The meta-regression analysis for algorithm studies with non-allegiant status as the predictor of the diagnostic odds ratio showed that non-allegiant status was a significant predictor of the diagnostic odds ratio ($p < 0.0001$) and explained a substantial amount of the observed heterogeneity (51.5%).

Quality assessment

For peer review only - http://bmjopen.bmj.com/site/about/guidelines.xhtml

The results of the quality assessment using QUADAS-2 are given in table 3 for the studies reporting on the diagnostic performance of the algorithm scoring method. In the patient selection domain, more non-allegiant studies (65%, 13/20) than allegiant (29%, 2/7) met the criterion for consecutive referrals. There were no marked differences on the other two criteria in this domain (avoid case-control design, avoid inappropriate exclusions). In the index test domain, the proportion of studies reporting that the PHQ-9 was conducted blind to the reference test was comparable between the two groups. There were differences in this domain for those studies using a translated version of the test. All non-English allegiant studies (5/5) used an appropriately translated version of the PHQ-9; whereas just over a half of the non-allegiant studies reported this (55%, 6/11). However, the majority of both sets of studies did not report details of psychometric properties of the translated version. For the reference test domain, nearly all studies in both groups were rated as using a reference test that would correctly classify the condition. While most allegiant studies reported that the reference test was interpreted blind to the PHQ-9 score (86%, 6/7), this was reported in only 60% (12/20) of the non-allegiant studies.

The two sets of studies that used translated versions of the reference test were broadly comparable. There was a slight indication that the allegiant studies were more likely to use an appropriately translated version of the reference test and report data on the psychometric properties of the translated version, though the numbers for the translated comparison are very low. There were, however, some more notable differences on the flow and timing domain. Most allegiant studies ensured that the time between the index and reference test was under two weeks (86%, 6/7) in comparison to 70% (14/20) of the non-allegiant studies. More allegiant studies met the criterion for ‘all participants included in the analysis’ (57%, 4/7) than non-allegiant studies (25%).

Summed items scoring method (cut-off point 10 or above)

For peer review only - http://bmjopen.bmj.com/site/about/guidelines.xhtml

Descriptive characteristics

Table 2 presents the sample characteristics of the thirty-one PHQ-9 validation studies that reported the psychometric properties of the PHQ-9 at cut-off point 10 or above. Five of these studies were co-authored by the original developers of the instrument or acknowledged collaboration [12], [23], [26], [49] or were co-authored by the first author of a previous study that had also been co-authored by one of the developers [28]. Twenty-six studies were conducted by independent researchers.

Three (60%, 3/5) allegiant studies [26], [28], [49] and eleven non-allegiant studies (42%, 11/26) [30]–[32], [34], [37], [38], [50]–[54] were conducted in hospital settings.

Three (60%, 3/5) allegiant studies[12], [26], [49] and thirteen non-allegiant studies (13/26) [30], [37], [38], [42], [48], [51]–[53], [55]–[59], were conducted in English.

The mean prevalence of major depressive disorder in the allegiant group was 13.2% (range 6.1% - 33.5%) and in the non-allegiant group was 16.1% (range 2.5% - 43.2%). The mean age of patients in the allegiant group studies was 48.1 (range 41.9 -61.0) and in the 26 non-allegiant studies that reported these data was 49.1 (range 23.0 – 78.0). The percentage of females in the allegiant studies that reported these data [12], [23], [26], [28] was 56.3% (range 28.6% – 67.8%) and in the non-allegiant group was 64.9 % (range 12% -100%).

For peer review only - http://bmjopen.bmj.com/site/about/guidelines.xhtml

Three allegiant studies used the self-reported mode of administration and two of them did not specify how the PHQ-9 was administered. In 9 non-allegiant studies (34%, 9/26) the PHQ-9 was administered by the researcher [30]–[32], [48], [56], [58]–[61]. All allegiant studies used SCID as a gold standard; the non-allegiant studies used a wider range of gold standards including SCAN, CIDI, MINI, CIS-R, C-DIS, though the SCID was used in half of the studies (50%, 13/26 studies).

Three allegiant studies (60%) did not include a conflict of interests statement [12], [23], [49]. Two of these studies [12], [23] acknowledged funding from Pfizer. None of the allegiant studies acknowledged collaboration or authorship of one of the developers of the PHQ-9.

Of the non-allegiant studies, thirteen (42%) did not include a conflict of interests statement [30]–[32], [37], [42], [46], [48], [53], [55], [60], [62]–[64]. Similar to the algorithm studies, the newer studies were more likely to include a conflict of interest statement. Funding was acknowledged by most studies (27/31) and most received funding from academic or/and health research institutions. One study [57] acknowledged that the last author involved in the development of one of the instruments (CORE-OM), 'but does not gain financially from its use'. One study [51] acknowledged funding from industry, AHA Pharmaceuticals Roundtable, but stated that 'the funding organisations had no role in the design or conduct of the study, collection, management, analysis or interpretation of data; or preparation, review or approval of the manuscript. Fine et al., 2013 disclosed that the last author had financial and consulting interests (Pfizer was not cited as one of them).

Diagnostic test accuracy

Pooled sensitivity of allegiant studies was 0.87 (95% CI = 0.77 – 0.93), pooled specificity was 0.87 (95% CI = 0.76 – 0.94), and the pooled diagnostic odds ratio was 49.31 (95% CI = 25.74 – 94.48) – see table 5. Heterogeneity was moderate (I^2 = 55.1%). Figure 2 represents the summary ROCs for this group.

For peer review only - http://bmjopen.bmj.com/site/about/guidelines.xhtml

----

Figure 2. PHQ-9 summed items scoring method at cut-off point 10 summary ROC plot for diagnosis of major depressive disorder in allegiant studies (panel A) and non-allegiant studies (panel B). Pooled sensitivity and specificity using a bi-variate meta-analysis (*HSROC* hierarchical receiver-operating characteristic).

Pooled sensitivity of non-allegiant studies was 0.76 (95% CI, 0.67 – 0.83), pooled specificity was 0.88 95% CI (0.85 – 0.91), and the pooled diagnostic odds ratio was 24.96 (95% CI 14.81 – 42.08), approximately half that of the allegiant studies (table 2). Heterogeneity was high at I^2 = 81.5 %. Figure 2 represents the summary ROCs for this group.

For peer review only - http://bmjopen.bmj.com/site/about/guidelines.xhtml

The meta-regression for the studies using a cut-off point of 10 or above with allegiance status of the predictor showed that allegiance status was a significant predictor of the diagnostic odds ratio (P = 0.015) and explained 19.0% of observed heterogeneity.

Quality assessment

The results of the quality assessment using the QUADAS-2 are given in table 4. For the patient selection domain, the two groups of studies were broadly comparable on two items (consecutive or random sample, avoid case-control design). However, all allegiant studies were rated as avoiding inappropriate exclusions (5/5) in contrast to 58% (15/26) of the non-allegiant studies.

On the index test domain, there were a number of differences between the two groups of studies. More of the non-allegiant studies (81%, 21/26) reported that the PHQ-9 was interpreted blind to the reference test compared to 60% (3/5) of the allegiant studies. All (5/5) allegiant studies were rated as pre-specifying the threshold on the PHQ-9 compared to 73% (19/26) of the non-allegiant studies. The two sets of studies were broadly comparable in terms of two items from the reference test domain (correctly classify target condition, reference test interpreted blind). Only one allegiant study used a translated version of the index test or reference test, so it is not possible to comment on differences between the two sets of studies in terms of these items from the index or reference test domains. For the flow and timing domain, the two groups of studies were broadly comparable for two of the criteria (interval of two weeks or less, all participants receive same reference test). However, fewer than half of the non-allegiant studies met the criterion for 'all participants included in the analysis' (42%, 11/26); whereas all allegiant studies met this criterion.

Discussion

For peer review only - http://bmjopen.bmj.com/site/about/guidelines.xhtml

For peer review only

This is to our knowledge the first systematic examination of a possible ‘allegiance’ or authorship effect in the validation of screening or case finding psychological instrument for a common mental health disorder. We reviewed diagnostic validation studies of the PHQ-9, a widely used depression screening-instrument. We found that allegiant studies reported higher sensitivity paired with similar specificity compared to non-allegiant studies. When entered as a covariate in meta-regression analyses, allegiance status was predictive of variation in the DOR for both the algorithm scoring method and the summed-item scoring method at a cut-off point of 10 or above.

Previous research has proposed several possible explanations for the allegiance effect [9]–[11]. One possibility is the advertent bias that may serve to inflate the performance of a test when evaluated by those who have developed it. However, before concluding that the differences are due to this, it is important to explore and rule out alternative explanations. First, it is possible that any observed differences are a result of differences in study characteristics of the two sets of studies (e.g., setting, clinical population). Secondly, differences in the methodological quality of the studies may also account for any differences. These possibilities are examined below.

Difference in study characteristics as potential alternative explanations

The two sets of studies were broadly comparable in terms of gender and the prevalence of depression, so these variables are unlikely to offer an explanation for the differences. While there were some indications from both sets of comparisons that the PHQ-9 may have been researcher-administered more often in the independent studies, it is not immediately clear how this would lead to lowered diagnostic performance.

For peer review only - http://bmjopen.bmj.com/site/about/guidelines.xhtml

The diagnostic meta-analyses of the PHQ-9 [13], [14] have shown that the sensitivity and DOR of the PHQ-9 tends to be lower in hospital settings for both algorithm and summed-item scoring methods. Whilst the fact that proportionally more non-allegiant algorithm studies were conducted in secondary care could explain the lower sensitivity and DOR values in the algorithm studies, in the studies that reported the cut-off point of or above this would not be the case as proportionally more allegiant studies were conducted in hospital settings.

Similarly, differences in the proportions of studies using translated versions of the PHQ-9 are also unlikely to offer an obvious explanation of the difference in diagnostic performance, because in the algorithm set of studies more of the allegiant studies used a translated version of the test, but the proportions were in the opposite direction for the studies using a cut off of 10 or above. We tested this by carrying out a sensitivity analysis restricting the sample to English studies and studies with adequate translation. The allegiance effect was still predictive of DOR variation between allegiance and non-allegiance studies variation in both algorithm ($p = 0.00$) and summed item scoring at cut-off point of 10 meta-analyses ($p = 0.02$).

A similar conclusion is also likely to apply to the age of the samples. There were more older adults studies in the non-allegiant than allegiant studies in the algorithm comparison. Depression could be more difficult to identify in older adults due to physical co-morbidities that may present with similar symptomatology to depression and could account for the lower diagnostic performance in the non-allegiant studies. However, the non-allegiant samples in the studies that reported the psychometric properties at cut-off point 10 or above had younger samples than the allegiant studies, so this would not support this interpretation.

The SCID was used as the gold standard in nearly all allegiant studies. The fact that some non-allegiant studies used other gold standards could potentially explain the poorer psychometric properties of the PHQ-9 in these studies. The SCID is often regarded as the most valid of the available semi-structured interviews used in depression diagnostic validity studies as the reference standard. If we assume that this is the case and, furthermore, that the PHQ-9 is an accurate method of screening for depression, then the PHQ-9 may be more likely to agree with the SCID

For peer review only - http://bmjopen.bmj.com/site/about/guidelines.xhtml

than other reference standards. However, when we carried out a sensitivity analysis restricting the sample to SCID only studies the allegiance effect was still predictive of DOR variation between allegiance and non-allegiance studies variation in both algorithm (p = 0.01) and summed item scoring at cut-off point of 10 reviews (p= 0.02).

Differences in methodological quality as potential alternative explanations

The quality of the studies was evaluated using the QUADAS-2. Although there were several potential methodological differences between the two groups of studies from the algorithm papers, not all of these offer obvious explanations of the observed differences and some are unlikely as explanations. For example, more allegiant studies ensured that the reference test was interpreted blind to the index test. This is unlikely to account for the observed differences, because a lack of blinding is typically associated with artificially increased diagnostic performance, which is in the opposite direction to the pattern of results observed here. The impact of some other differences is less clear-cut. For example, a higher number of the non-allegiant studies met the criterion for consecutive referrals. For this to provide an explanation of the of the observed differences, the non-consecutive nature of the referrals in the studies by those who had developed the PHQ-9 would need to have led to the over-inclusion of true positives or under-inclusion of false negatives given that these studies tended to report higher sensitivity relative to the non-allegiant studies (and vice versa for the independent studies). It is not immediately obvious how this would occur. The allegiant studies were more likely to have met the criterion of 'included all participants in the analysis'. It is possible that the greater loss of participants from the non-allegiant studies may have artificially reduced the observed diagnostic accuracy, though, again, it is not immediately obvious how this would have affected the true positive and false negative rates. Although there is not an obvious explanation of how these differences in methodological quality could account for the observed differences in diagnostic performance, it is important to recognise that they cannot on that basis be ruled out.

For peer review only - http://bmjopen.bmj.com/site/about/guidelines.xhtml

There are, however, two differences in methodological quality among the algorithm studies that are clearer potential alternative explanations. The higher rate of appropriate translations among the allegiant studies is potentially important, because lower diagnostic estimates may be expected from studies that have poorly translated versions of the index test. In the flow and timing domain, more allegiant studies ensured that there was a less than two-week interval between the index and reference test. This is consistent with lower diagnostic performance in the non-allegiant studies: as the interval increases it is likely that depression status may change and this would lead to lower levels of agreement between the index test and the reference test.

There were also differences on some quality assessment items between the two sets of studies in the summed item scoring method comparison. The threshold was reported as pre-specified in all allegiant studies in contrast to approximately three quarters of the non-allegiant studies. On the face of it, this is unlikely to explain the observed differences, because the use of a pre-specified cut-off point is likely to be associated with lower not higher diagnostic test performance. One possibility, however, is that studies that performed poorly at this cut-off point were less likely to be reported by those who had developed the measure. As discussed in more detail in the limitations section, we were unable to explore this possibility through the use of formal tests for publication bias.

All allegiant studies avoided inappropriate exclusions compared to approximately half of the non-allegiant studies. While this is a potential alternative explanation of the differences it is not immediately obvious how this would explain the differences in diagnostic performance between the two sets of studies. Fewer than half of the non-allegiant studies met the criterion for ‘all participants included in the analysis’, in contrast to all of the allegiant studies met this criterion, but again this difference should usually work against the inclusive studies, not those

For peer review only - http://bmjopen.bmj.com/site/about/guidelines.xhtml

excluding cases. More of the non-allegiant studies reported that the PHQ-9 was interpreted blind to the reference test. This does offer a potential explanation, because the absence of blinding may artificially inflate diagnostic accuracy.

Limitations

The results of this review need to be viewed in the light of the limitations of the primary studies that contributed to the review and the review itself. An important consideration is to establish whether any observed differences between the diagnostic performance of the non-allegiant and allegiant studies are better accounted for by study characteristic or methodological differences. Caution, however, is needed in interpreting any differences, because of the small number of allegiant studies in both the algorithm and cut-off 10 or above comparisons. The small number of allegiant studies also meant that we were also unable to explore the potential role of publication bias in the non-allegiant and allegiant studies. At least 10 studies are required to use standard methods of examining publication bias, but the number of allegiant studies in both the algorithm and cut-off 10 or above comparisons were fewer than this. Papers published from August 2013 onwards are not covered in the literature search used and so it potentially misses some more recent studies that would be eligible for inclusion although it is unlikely that many, if any, new allegiant studies have been published since.

Conclusions and implications for further research.

For peer review only - http://bmjopen.bmj.com/site/about/guidelines.xhtml

The aims of the review was to investigate whether an allegiance effect is found that leads to an increased diagnostic performance in diagnostic validation studies that were conducted by teams connected to the original developers of the PHQ-9. Our analyses showed that diagnostic studies conducted by independent/non-allegiant researchers had lower sensitivity paired with similar specificity compared to studies that were classified as allegiant. This conclusion held for both the algorithm and cut-off 10 or above studies. We explored a range of possible alternative explanations for the observed allegiance effect including both differences in study characteristics and study quality. A number of potential differences were found, though for some of these it is not clear how they would necessarily account for the observed differences. However, there were a number of differences that offered potential alternative explanations unconnected to allegiance effects. In the algorithm studies, the studies rated as allegiant were also more likely to use an appropriate translation of the PHQ-9 and were also more likely to ensure that the index and reference test were conducted within two weeks of each other, both of which may be associated with an improvement in observed diagnostic performance of an instrument. The majority of studies in both meta-analyses did not provide clear statements about potential conflict of interest and/or funding, however the newer studies were more likely to provide such statements, which may reflect increasing transparency in this area of research.

We cannot, therefore, conclude that allegiance effects are present in studies examining the diagnostic performance of the PHQ-9; but nor can we rule them out. Conflicts of interest are an important area of investigation in medical and behavioural research, particularly due to concerns about trial results being influenced by industry sponsorship. Future diagnostic validity in this area should as a matter of routine present clear statements about potential conflicts of interest and funding, particularly relating to the development of the instrument under evaluation. Future meta-analyses of diagnostic validation studies of psychological measures should routinely evaluate the impact of researcher allegiance in the primary studies examined in the meta-analysis.

For peer review only - http://bmjopen.bmj.com/site/about/guidelines.xhtml

Contributors LM led on all stages of the review and is the guarantor. We used an established database of diagnostic validation studies of the PHQ-9 [13], [14] SG provided expert advice on methodology and approaches to assessment of the evidence base. AM carried out the literature searches, screened the studies, extracted data and assessed the quality of the included studies for one of the systematic reviews (Moriarty et al., 2015) . LM carried out the literature searches, screened the studies, extracted data and assessed the quality of the included studies for the other systematic review (Manea et al., 2015), analysed the data for both systematic reviews and drafted the report. JB was involved in the development of the study, wrote the introduction section of the review and contributed to the production of the final report. DM supervised the quality assessment, methodology and approaches to evidence synthesis, provided senior advice and support throughout and contributed to the production of the final report. All parties were involved in drafting and/or commenting on the report.

Competing interests None declared.

Funding LM was an NIHR Clinical Lecturer when this research was carried out. The NIHR had no role in the study design, methods, data collection, analysis or interpretation of data, nor any role in the preparation of the manuscript or decision to submit the manuscript for publication.

Provenance and peer review Not commissioned; externally peer reviewed.

Data sharing statement No additional data are available.

For peer review only

For peer review only - http://bmjopen.bmj.com/site/about/guidelines.xhtml

REFERENCES

[1] L. Luborsky, L. Diguer, D. A. Seligman, R. Rosenthal, E. D. Krause, S. Johnson, G. Halperin, M. Bishop, J. S. Berman, and E. Schweizer, “The Researcher’s Own Therapy Allegiances: A ‘Wild Card’ in Comparisons of Treatment Efficacy,” *Clin. Psychol. Sci. Pract.*, vol. 6, no. 1, pp. 95–106, May 2006.

[2] E. Dragioti, I. Dimoliatis, and E. Evangelou, “Disclosure of researcher allegiance in meta-analyses and randomised controlled trials of psychotherapy: a systematic appraisal,” *BMJ Open*, vol. 5, no. 6, pp. e007206–e007206, Jun. 2015.

[3] T. Munder, O. Brütsch, R. Leonhart, H. Gerger, and J. Barth, “Researcher allegiance in psychotherapy outcome research: An overview of reviews,” *Clin. Psychol. Rev.*, vol. 33, no. 4, pp. 501–511, Jun. 2013.

[4] D. A. Winter, “Editorial.” Routledge, 07-May-2010.

[5] J. McLeod, “Taking allegiance seriously–implications for research policy and practice,” *Eur. J. Psychother. Couns.*, May 2010.

[6] G. L. Staines and C. M. Cleland, “Bias in meta-analytic estimates of the absolute efficacy of psychotherapy.,” *Rev. Gen. Psychol.*, vol. 11, no. 4, pp. 329–347, 2007.

[7] K. D. Markman and E. R. Hirt, “Social Prediction and the ‘Allegiance Bias,’” *Soc. Cogn.*, vol. 20, no. 1, pp. 58–86, Feb. 2002.

[8] G. D. Walters, “The Psychological Inventory of Criminal Thinking Styles and Psychopathy Checklist: Screening version as incrementally valid predictors of recidivism.,” *Law Hum. Behav.*, vol. 33, no. 6, pp. 497–505, 2009.

[9] J. P. Singh, M. Grann, and S. Fazel, “Authorship Bias in Violence Risk Assessment? A Systematic Review and Meta-Analysis,” *PLoS One*, vol. 8, no. 9, p. e72484, Sep. 2013.

For peer review only

For peer review only - http://bmjopen.bmj.com/site/about/guidelines.xhtml

[10] P. R. Blair, D. K. Marcus, and M. T. Boccaccini, “Is There an Allegiance Effect for Assessment Instruments? Actuarial Risk Assessment as an Exemplar,” *Clin. Psychol. Sci. Pract.*, vol. 15, no. 4, pp. 346–360, Oct. 2008.

[11] S. O. Lilienfeld and M. K. Jones, “Allegiance Effects in Assessment: Unresolved Questions, Potential Explanations, and Constructive Remedies,” *Clin. Psychol. Sci. Pract.*, vol. 15, no. 4, pp. 361–365, Oct. 2008.

[12] K. Kroenke, R. L. Spitzer, and J. B. Williams, “The PHQ-9: validity of a brief depression severity measure.,” *J. Gen. Intern. Med.*, vol. 16, no. 9, pp. 606–13, Sep. 2001.

[13] L. Manea, S. Gilbody, and D. McMillan, “A diagnostic meta-analysis of the Patient Health Questionnaire-9 (PHQ-9) algorithm scoring method as a screen for depression,” *Gen. Hosp. Psychiatry*, vol. 37, no. 1, pp. 67–75, Jan. 2015.

[14] A. S. Moriarty, S. Gilbody, D. McMillan, and L. Manea, “Screening and case finding for major depressive disorder using the Patient Health Questionnaire (PHQ-9): a meta-analysis,” *Gen. Hosp. Psychiatry*, vol. 37, no. 6, pp. 567–576, Nov. 2015.

[15] P. F. Whiting, A. W. S. Rutjes, M. E. Westwood, S. Mallett, J. J. Deeks, J. B. Reitsma, M. M. G. Leeflang, J. A. C. Sterne, P. M. M. Bossuyt, and QUADAS-2 Group, “QUADAS-2: a revised tool for the quality assessment of diagnostic accuracy studies.,” *Ann. Intern. Med.*, vol. 155, no. 8, pp. 529–36, Oct. 2011.

[16] R. Mann, C. E. Hewitt, and S. M. Gilbody, “Assessing the quality of diagnostic studies using psychometric instruments: applying QUADAS,” *Soc. Psychiatry Psychiatr. Epidemiol.*, vol. 44, no. 4, pp. 300–307, Apr. 2009.

[17] J. B. Reitsma, A. S. Glas, A. W. S. Rutjes, R. J. P. M. Scholten, P. M. Bossuyt, and A. H. Zwinderman, “Bivariate analysis of sensitivity and specificity produces informative summary measures in diagnostic reviews,” *J. Clin. Epidemiol.*, vol. 58, no. 10, pp. 982–990, Oct. 2005.

For peer review only

[18] University of York. NHS Centre for Reviews and Dissemination., *Systematic reviews : CRD's guidance for undertaking reviews in health care*. CRD, University of York, 2009.

[19] S. D. Walter, "Properties of the summary receiver operating characteristic (SROC) curve for diagnostic test data," *Stat. Med.*, vol. 21, no. 9, pp. 1237–1256, May 2002.

[20] J. G. Lijmer, P. M. M. Bossuyt, and S. H. Heisterkamp, "Exploring sources of heterogeneity in systematic reviews of diagnostic tests," *Stat. Med.*, vol. 21, no. 11, pp. 1525–1537, Jun. 2002.

[21] S. G. Thompson and J. P. T. Higgins, "How should meta-regression analyses be undertaken and interpreted?," *Stat. Med.*, vol. 21, no. 11, pp. 1559–1573, Jun. 2002.

[22] C. Diez-Quevedo, T. Rangil, L. Sanchez-Planell, K. Kroenke, and R. L. Spitzer, "Validation and utility of the patient health questionnaire in diagnosing mental disorders in 1003 general hospital Spanish inpatients.," *Psychosom. Med.*, vol. 63, no. 4, pp. 679–86.

[23] K. Gräfe, S. Zipfel, W. Herzog, and B. Löwe, "Screening psychischer Störungen mit dem "Gesundheitsfragebogen für Patienten (PHQ-D)"," *Diagnostica*, vol. 50, no. 4, pp. 171–181, Oct. 2004.

[24] B. Löwe, R. L. Spitzer, K. Gräfe, K. Kroenke, A. Quenter, S. Zipfel, C. Buchholz, S. Witte, and W. Herzog, "Comparative validity of three screening questionnaires for DSM-IV depressive disorders and physicians' diagnoses.," *J. Affect. Disord.*, vol. 78, no. 2, pp. 131–40, Feb. 2004.

[25] R. L. Spitzer, K. Kroenke, and J. B. Williams, "Validation and utility of a self-report version of PRIME-MD: the PHQ primary care study. Primary Care Evaluation of Mental Disorders. Patient Health Questionnaire.," *JAMA*, vol. 282, no. 18, pp. 1737–44, Nov. 1999.

[26] P. Thekkumpurath, J. Walker, I. Butcher, L. Hodges, A. Kleiboer, M. O'Connor, L. Wall, G. Murray, K. Kroenke, and M. Sharpe,

For peer review only - http://bmjopen.bmj.com/site/about/guidelines.xhtml

"Screening for major depression in cancer outpatients: the diagnostic accuracy of the 9-item patient health questionnaire.," *Cancer*, vol. 117, no. 1, pp. 218–27, Jan. 2011.

[27] K. Muramatsu, H. Miyaoka, K. Kamijima, Y. Muramatsu, M. Yoshida, T. Otsubo, and F. Gejyo, "The patient health questionnaire, Japanese version: validity according to the mini-international neuropsychiatric interview-plus.," *Psychol. Rep.*, vol. 101, no. 3 Pt 1, pp. 952–60, Dec. 2007.

[28] R. Navinés, P. Castellví, J. Moreno-España, D. Gimenez, M. Udina, S. Cañizares, C. Diez-Quevedo, M. Valdés, R. Solà, and R. Martín-Santos, "Depressive and anxiety disorders in chronic hepatitis C patients: Reliability and validity of the Patient Health Questionnaire," *J. Affect. Disord.*, vol. 138, no. 3, pp. 343–351, May 2012.

[29] S. M. Eack, C. G. Greeno, and B.-J. Lee, "Limitations of the Patient Health Questionnaire in Identifying Anxiety and Depression: Many Cases Are Undetected.," *Res. Soc. Work Pract.*, vol. 16, no. 6, pp. 625–631, Nov. 2006.

[30] J. R. Fann, C. H. Bombardier, S. Dikmen, P. Esselman, C. A. Warms, E. Pelzer, H. Rau, and N. Temkin, "Validity of the Patient Health Questionnaire-9 in assessing depression following traumatic brain injury.," *J. Head Trauma Rehabil.*, vol. 20, no. 6, pp. 501–11.

[31] B. Gelaye, M. A. Williams, S. Lemma, N. Deyessa, Y. Bahretibeb, T. Shibre, D. Wondimagegn, A. Lemenhe, J. R. Fann, A. Vander Stoep, and X.-H. H. Andrew Zhou, "Validity of the patient health questionnaire-9 for depression screening and diagnosis in East Africa," *Psychiatry Res.*, vol. 210, no. 2, pp. 653–661, Dec. 2013.

[32] T. Hyphantis, K. Kotsis, P. V. Voulgari, N. Tsifetaki, F. Creed, and A. A. Drosos, "Diagnostic accuracy, internal consistency, and convergent validity of the Greek version of the patient health questionnaire 9 in diagnosing depression in rheumatologic disorders," *Arthritis Care Res. (Hoboken).*, vol. 63, no. 9, pp. 1313–1321, Sep. 2011.

For peer review only

For peer review only - http://bmjopen.bmj.com/site/about/guidelines.xhtml

[33] M. Inagaki, T. Ohtsuki, N. Yonemoto, Y. Kawashima, A. Saitoh, Y. Oikawa, M. Kurosawa, K. Muramatsu, T. A. Furukawa, and M. Yamada, “Validity of the Patient Health Questionnaire (PHQ)-9 and PHQ-2 in general internal medicine primary care at a Japanese rural hospital: a cross-sectional study.,” *Gen. Hosp. Psychiatry*, vol. 35, no. 6, pp. 592–7, Jan. 2013.

[34] M. E. Khamseh, H. R. Baradaran, A. Javanbakht, M. Mirghorbani, Z. Yadollahi, and M. Malek, “Comparison of the CES-D and PHQ-9 depression scales in people with type 2 diabetes in Tehran, Iran,” *BMC Psychiatry*, vol. 11, no. 1, p. 61, Dec. 2011.

[35] P. Persoons, K. Luyckx, C. Desloovere, J. Vandenberghe, and B. Fischler, “Anxiety and mood disorders in otorhinolaryngology outpatients presenting with dizziness: validation of the self-administered PRIME-MD Patient Health Questionnaire and epidemiology.,” *Gen. Hosp. Psychiatry*, vol. 25, no. 5, pp. 316–23.

[36] A. Picardi, D. A. Adler, D. Abeni, H. Chang, P. Pasquini, W. H. Rogers, and K. M. Bungay, “Screening for depressive disorders in patients with skin diseases: a comparison of three screeners.,” *Acta Derm. Venereol.*, vol. 85, no. 5, pp. 414–9, 2005.

[37] L. Stafford, M. Berk, and H. J. Jackson, “Validity of the Hospital Anxiety and Depression Scale and Patient Health Questionnaire-9 to screen for depression in patients with coronary artery disease,” *Gen. Hosp. Psychiatry*, vol. 29, no. 5, pp. 417–424, Sep. 2007.

[38] B. D. Thombs, R. C. Ziegelstein, and M. A. Whooley, “Optimizing detection of major depression among patients with coronary artery disease using the patient health questionnaire: data from the heart and soul study.,” *J. Gen. Intern. Med.*, vol. 23, no. 12, pp. 2014–7, Dec. 2008.

[39] A. W. Thompson, H. Liu, R. D. Hays, W. J. Katon, R. Rausch, N. Diaz, E. L. Jacob, S. D. Vassar, and B. G. Vickrey, “Diagnostic accuracy and agreement across three depression assessment measures for Parkinson’s disease,” *Parkinsonism Relat. Disord.*, vol. 17, no. 1, pp. 40–45, Jan. 2011.

For peer review only - http://bmjopen.bmj.com/site/about/guidelines.xhtml

[40] A. Turner, J. Hambridge, J. White, G. Carter, K. Clover, L. Nelson, and M. Hackett, “Depression screening in stroke: a comparison of alternative measures with the structured diagnostic interview for the diagnostic and statistical manual of mental disorders, fourth edition (major depressive episode) as criterion standard.,” *Stroke.*, vol. 43, no. 4, pp. 1000–5, Apr. 2012.

[41] K. M. van Steenbergen-Weijenburg, L. de Vroege, R. R. Ploeger, J. W. Brals, M. G. Vloedbeld, T. F. Veneman, L. Hakkaart-van Roijen, F. F. Rutten, A. T. Beekman, and C. M. van der Feltz-Cornelis, “Validation of the PHQ-9 as a screening instrument for depression in diabetes patients in specialized outpatient clinics,” *BMC Health Serv. Res.*, vol. 10, no. 1, p. 235, Dec. 2010.

[42] B. Arroll, F. Goodyear-Smith, S. Crengle, J. Gunn, N. Kerse, T. Fishman, K. Falloon, and S. Hatcher, “Validation of PHQ-2 and PHQ-9 to Screen for Major Depression in the Primary Care Population,” *Ann. Fam. Med.*, vol. 8, no. 4, pp. 348–353, Jul. 2010.

[43] L. Ayalon, M. Goldfracht, and P. Bech, “‘Do you think you suffer from depression?’ Reevaluating the use of a single item question for the screening of depression in older primary care patients,” *Int. J. Geriatr. Psychiatry*, vol. 25, no. 5, pp. 497–502, May 2010.

[44] V. Henkel, R. Mergl, R. Kohnen, A.-K. Allgaier, H.-J. Möller, and U. Hegerl, “Use of brief depression screening tools in primary care: consideration of heterogeneity in performance in different patient groups,” *Gen. Hosp. Psychiatry*, vol. 26, no. 3, pp. 190–198, May 2004.

[45] F. Lamers, C. C. M. Jonkers, H. Bosma, B. W. J. H. Penninx, J. A. Knottnerus, and J. T. M. van Eijk, “Summed score of the Patient Health Questionnaire-9 was a reliable and valid method for depression screening in chronically ill elderly patients,” *J. Clin. Epidemiol.*, vol. 61, no. 7, pp. 679–687, Jul. 2008.

[46] M. Lotrakul, S. Sumrithe, and R. Saipanish, “Reliability and validity of the Thai version of the PHQ-9,” *BMC Psychiatry*, vol. 8, no. 1, p. 46, Dec. 2008.

[47] N. P. Zuithoff, Y. Vergouwe, M. King, I. Nazareth, M. J. van Wezep, K. G. Moons, and M. I. Geerlings, “The Patient Health

For peer review only - http://bmjopen.bmj.com/site/about/guidelines.xhtml

Questionnaire-9 for detection of major depressive disorder in primary care: consequences of current thresholds in a crosssectional study," *BMC Fam. Pract.*, vol. 11, no. 1, p. 98, Dec. 2010.

[48] D. Gjerdingen, S. Crow, P. McGovern, M. Miner, and B. Center, "Postpartum depression screening at well-child visits: validity of a 2-question screen and the PHQ-9.," *Ann. Fam. Med.*, vol. 7, no. 1, pp. 63–70, 2009.

[49] L. S. Williams, E. J. Brizendine, L. Plue, T. Bakas, W. Tu, H. Hendrie, and K. Kroenke, "Performance of the PHQ-9 as a screening tool for depression after stroke.," *Stroke.*, vol. 36, no. 3, pp. 635–8, Mar. 2005.

[50] M. H. N. Chagas, V. Tumas, G. R. Rodrigues, J. P. Machado-de-Sousa, A. S. Filho, J. E. C. Hallak, and J. A. S. Crippa, "Validation and internal consistency of Patient Health Questionnaire-9 for major depression in Parkinson's disease," *Age Ageing*, vol. 42, no. 5, pp. 645–649, Sep. 2013.

[51] L. Elderon, K. G. Smolderen, B. Na, and M. A. Whooley, "Accuracy and prognostic value of American Heart Association: recommended depression screening in patients with coronary heart disease: data from the Heart and Soul Study.," *Circ. Cardiovasc. Qual. Outcomes*, vol. 4, no. 5, pp. 533–40, Sep. 2011.

[52] A. G. Rooney, S. McNamara, M. Mackinnon, M. Fraser, R. Rampling, A. Carson, and R. Grant, "Screening for major depressive disorder in adults with cerebral glioma: an initial validation of 3 self-report instruments," *Neuro. Oncol.*, vol. 15, no. 1, pp. 122–129, Jan. 2013.

[53] S. Watnick, P.-L. Wang, T. Demadura, and L. Ganzini, "Validation of 2 depression screening tools in dialysis patients.," *Am. J. Kidney Dis.*, vol. 46, no. 5, pp. 919–24, Nov. 2005.

[54] Y. Zhang, R. Ting, M. Lam, J. Lam, H. Nan, R. Yeung, W. Yang, L. Ji, J. Weng, Y.-K. Wing, N. Sartorius, and J. C. N. Chan, "Measuring depressive symptoms using the Patient Health Questionnaire-9 in Hong Kong Chinese subjects with type 2 diabetes," *J.*

For peer review only - http://bmjopen.bmj.com/site/about/guidelines.xhtml

528 *Affect. Disord.*, vol. 151, no. 2, pp. 660–666, Nov. 2013.

529 [55] A. O. Adewuya, B. A. Ola, and O. O. Afolabi, "Validity of the patient health questionnaire (PHQ-9) as a screening tool for depression
530 amongst Nigerian university students," *J. Affect. Disord.*, vol. 96, no. 1–2, pp. 89–93, Nov. 2006.

531 [56] T. H. Fine, A. A. Contractor, M. Tamburrino, J. D. Elhai, M. R. Prescott, G. H. Cohen, E. Shirley, P. K. Chan, T. Goto, R. Slembarski, I.
532 Liberzon, S. Galea, and J. R. Calabrese, "Validation of the telephone-administered PHQ-9 against the in-person administered SCID-I
533 major depression module," *J. Affect. Disord.*, vol. 150, no. 3, pp. 1001–1007, Sep. 2013.

534 [57] S. Gilbody, D. Richards, S. Brealey, and C. Hewitt, "Screening for depression in medical settings with the Patient Health Questionnaire
535 (PHQ): A diagnostic meta-analysis," *J. Gen. Intern. Med.*, vol. 22, no. 11, pp. 1596–1602, Oct. 2007.

536 [58] E. Phelan, B. Williams, K. Meeker, K. Bonn, J. Frederick, J. LoGerfo, and M. Snowden, "A study of the diagnostic accuracy of the PHQ-
537 9 in primary care elderly," *BMC Fam. Pract.*, vol. 11, no. 1, p. 63, Dec. 2010.

538 [59] A. C. Sidebottom, P. A. Harrison, A. Godecker, and H. Kim, "Validation of the Patient Health Questionnaire (PHQ)-9 for prenatal
539 depression screening," *Arch. Womens. Ment. Health*, vol. 15, no. 5, pp. 367–374, Oct. 2012.

540 [60] F. de Lima Osório, A. Vilela Mendes, J. A. Crippa, and S. R. Loureiro, "Study of the Discriminative Validity of the PHQ-9 and PHQ-2 in
541 a Sample of Brazilian Women in the Context of Primary Health Care," *Perspect. Psychiatr. Care*, vol. 45, no. 3, pp. 216–227, Jul. 2009.

542 [61] V. Patel, R. Araya, N. Chowdhary, M. King, B. Kirkwood, S. Nayak, G. Simon, and H. A. Weiss, "Detecting common mental disorders in
543 primary care in India: a comparison of five screening questionnaires," *Psychol. Med.*, vol. 38, no. 2, Feb. 2008.

544 [62] M. N. N. Azah, M. E. M. Shah, S. Juwita, I. S. Bahri, W. M. W. M. Rushidi, and Y. M. Jamil, "Validation of the Malay Version Brief
545 Patient Health Questionnaire (PHQ-9) among Adult Attending Family Medicine Clinics.," *Int. Med. J.*, 2005.

For peer review only - http://bmjopen.bmj.com/site/about/guidelines.xhtml

[63] S.-I. Liu, Z.-T. Yeh, H.-C. Huang, F.-J. Sun, J.-J. Tjung, L.-C. Hwang, Y.-H. Shih, and A. W.-C. Yeh, “Validation of Patient Health Questionnaire for depression screening among primary care patients in Taiwan,” *Compr. Psychiatry*, vol. 52, no. 1, pp. 96–101, Jan. 2011.

[64] K. Wittkampf, H. van Ravesteijn, K. Baas, H. van de Hoogen, A. Schene, P. Bindels, P. Lucassen, E. van de Lisdonk, and H. van Weert, “The accuracy of Patient Health Questionnaire-9 in detecting depression and measuring depression severity in high-risk groups in primary care,” *Gen. Hosp. Psychiatry*, vol. 31, no. 5, pp. 451–459, Sep. 2009.

For peer review only - http://bmjopen.bmj.com/site/about/guidelines.xhtml

BMJ Open

Table 1: Descriptive characteristics of algorithm studies (Manea et al., 2014)

Study	Sample characteristics (Country, setting, age, sex)	Sample size and % depressed	PHQ-9 characteristics	Diagnostic standard	a) Conflict of interest (COI) declaration b) Funding c) Relationship with original developers
Diez-Quevedo et al. (2001)	Country: Spain<br><br>Setting: Medical and surgical tertiary hospitals<br><br>Age (yrs): M=43 (SD=14.2)<br><br>Female: 45.6%	N = 1003<br>Depressed: 8.2%	Administration: Self-report<br>Language: Spanish	DSM-III-R SCID	a) No COI declaration<br>b) Funding acknowledged (academic institutions)<br>c) Not acknowledged
Gräfe et al. (2004)	Country: Germany<br><br>Setting: psychosomatic walk-in clinics and family practices<br><br>Age (yrs): M = 41.9 (SD = 13.8)<br><br>Female: 67.8%	N = 528<br><br>Depressed: 29.2% psychosomatic patients; 6.16% medical patients	Language: German<br><br>Administration: self-report	DSM-IV SCID	a) No COI declaration<br>b) Acknowledged funding from Pfizer<br>c) Not acknowledged

For peer review only

For peer review only - http://bmjopen.bmj.com/site/about/guidelines.xhtml

Lowe et al. (2004)	Country: Germany<br><br>Setting: Outpatient clinics and family practices<br>Age (yrs): M = 41.7 (SD = 13.8)<br>Female: 67.1%	N = 501<br><br>Depressed: 13.2%	Administration: Self-report<br>Language: German	DSM–IV SCID	a) COI declaration 'This study was supported by unrestricted restricted grants from Pfizer Germany and from the medical faculty of the University of Heidelberg Germany, and there are no COI.'<br>b) Acknowledged funding from Pfizer and academic institution<br>c) Not acknowledged
Muramatsu et al. (2007)	Country: Japan<br><br>Setting: Primary care and general hospital<br><br>Age (yrs): M = 43.3 (SD = 16.4)<br><br>Female: 59.5%	N = 131<br><br>Depressed: 28.2%	Administration: Self-report<br><br>Language: Japanese	DSM–IV MINI	a) No COI declaration<br>b) Acknowledged funding from Pfizer<br>c) Acknowledged one of the developers of the PHQ-9: 'The authors acknowledge Dr R L Spitzer'
Navinés et al. (2012)	Country: Spain<br><br>Setting: General hospital (patients with chronic HCV)<br><br>Age (yrs): M = 43.4 (SD = 10.2)<br><br>Female: 28.6%	N = 500<br>Depressed: 6.4%	Administration: Self-report<br><br>Language: Spanish	DSM–IV SCID	a) All authors declared that they had no COI.<br>b) Role of funding source declared<br>c) Not acknowledged

For peer review only - http://bmjopen.bmj.com/site/about/guidelines.xhtml

Spitzer et al. (1999)	Country: US<br><br>Setting: Primary care<br><br>Age (yrs): M = 46 (SD = 17.2)<br><br>Female: 66%	N = 3000 (585 received SCID)<br><br>Depressed: 10%	Administration: Self-report<br><br>Language: English	DSM-III-R SCID	a) No COI declaration<br>b) Acknowledged funding from Pfizer. 'Drs Spitzer and Williams receive honoraria and consulting money from Pfizer Inc, which has supported this work.'<br>c) N/A
Thekkumpurath et al. (2010)	Country: UK<br><br>Setting: Hospital (cancer patients)<br><br>Age (yrs): M = 61<br><br>Female: 63%	N = 782<br><br>Depressed: 6.3% (of the whole sample)	Administration: Not stated<br><br>Language: English	DSM-IV SCID	a) COI declaration: 'Supported by Cancer Research UK'<br>b) As in a)<br>c) Not acknowledged
Ayalon et al. (2010)	Country: Israel<br><br>Age (yrs): M = 75 (SD = 8.1)<br><br>Female: 40.5 %	N = 153<br>Depressed: 3.9 %	Administration: Researcher administered<br><br>Language: Hebrew	DSM-IV SCID	a) COI declaration: 'The project was funded by an Investigator's Initiated Research Grant from Lundbeck International given to Dr Liat Ayalon. Lundbeck International had no other involvement in the project concept of design or in this paper. Per Bech has occasionally over the past 3 years until August 2008 received funding from and has been speaker or member of advisory boards for pharmaceutical companies with an interest in the drug treatment of affective disorders (Astra-Zeneca, Lilly, H. Lundbeck A/S, Lundbeck Foundation and Organon). '<br>b) Acknowledged funding from Lundbeck International

For peer review only - http://bmjopen.bmj.com/site/about/guidelines.xhtml

Eack et al. (2006)	Country: US<br><br>Setting: Community mental health centers for children<br><br>Age (yrs): M = 39.20 (SD 9.63)<br><br>Female: 100%	N = 50<br><br>Depressed: 28%	Administration: Self-report<br><br>Language: English	DSM-IV SCID	a) No COI declaration<br>b) Funding acknowledged (academic /health research institutions)
Fann et al. (2005)	Country: US<br><br>Setting: Trauma hospital (inpatients with traumatic brain injury)<br><br>Age (yrs): M = 42 (SD=17.9)<br><br>Female: 29.1%	N = 135<br><br>Depressed: 16.3%	Administration: Telephone-administered<br><br>Language: English	DSM-IV SCID	a) No COI declaration<br>b) Funding acknowledged (academic institutions)

For peer review only - http://bmjopen.bmj.com/site/about/guidelines.xhtml

Gelaye et al. (2011)	Country: Ethiopia<br><br>Setting: General hospital<br><br>Age (yrs): 34.9 (SD=11.6)<br><br>Female: 63.1 %	N = 363<br>Depressed: 12.6%	Administration: Researcher-administered<br><br>Language: Amharic	DSM-IV SCAN	a) No COI declaration<br>b) Funding acknowledged (academic /health research institutions)
Gjerdingen et al. (2009)	Country: US<br><br>Setting: Community<br><br>Age (yrs): M = 29.3<br><br>Female: 100%	N = 438<br><br>Depressed: 4.6%	Administration: Telephone or self-report<br><br>Language: English	DSM-IV SCID	a) No COI declaration<br>b) Funding acknowledged (academic /health research institutions)
Henkel et al. (2004)	Country: Germany<br><br>Setting: primary care<br><br>Age (yrs): not reported<br><br>Female: 74%	N = 448<br><br>Depressed: 10%	Administration: self-report<br><br>Language: German	DSM-IV CIDI	a) No COI declaration<br>b) Funding acknowledged (academic /health research institutions)

For peer review only - http://bmjopen.bmj.com/site/about/guidelines.xhtml

Hyphantis et al. (2011)	Country: Greece<br><br>Setting: Hospital – rheumatology patients<br><br>Age (yrs): M = 54.2 (SD = 13.5)<br><br>Female: 74%	N = 213<br><br>Depressed: 32.4%	Administration: Researcher administered<br><br>Language: Greek	DSM-IV MINI	a) No COI declaration<br>b) No funding acknowledgement
Inagaki et al. (2013)	Country: Japan<br><br>Setting: General hospital<br><br>Age whole sample (yrs): M = 73.5 (SD = 12.3)<br><br>Female: 59.3%	N = 104 out of 511 received MINI<br><br>Depressed: 7.4%	Administration: Researcher administered<br>Language: Japanese	DSM-IV MINI	a) COI declaration: 'The authors declare that they have no competing interests.'<br><br>b) Funding acknowledged (academic /health research institutions)
Khamseh et al. (2011)	Country: Iran<br><br>Setting: Diabetes clinic<br><br>Age (yrs): M = 56.17 (SD = 9.60)<br><br>Female: 51.9%	N = 185<br><br>Depressed: 43.2%	Administration: Self report<br>Language: Persian	DSM-IV SCID	a) COI declaration: The authors declared no competing interests<br>b) Funding acknowledged (academic /health research institutions)

For peer review only - http://bmjopen.bmj.com/site/about/guidelines.xhtml

Lamers et al. (2008)	Country: Netherlands<br><br>Setting: Primary care (elderly)<br><br>Age (yrs): M = 71.4 (SD = 6.90)<br><br>Female: 48.2%	N = 713<br><br>Depressed: 10.7%	Administration: Self report<br><br>Language: Dutch	DSM-IV MINI	a) No COI declaration<br>b) Funding acknowledged (academic /health research institutions)
Lotrakul et al. (2008)	Country: Thailand<br><br>Setting: Primary care<br><br>Age (yrs): M = 45.0 (SD = 14.30)<br><br>Female: 73.7%	N = 279<br><br>Depressed: 6.8%	Administration: Self report<br><br>Language: Thai	DSM-IV MINI	a) No COI declaration<br>b) Funding acknowledged (academic /health research institutions)
Persoons et al. (2003)	Country: Belgium<br><br>Setting: Hospital (otolaryngology patients)<br><br>Age (yrs): M = 48.2 (SD = 12.9)<br><br>Female: 65.6%	N = 268 (97 received MINI)<br><br>Depressed: 16.5%	Administration: Self-report<br><br>Language: Dutch	DSM-IV MINI	a) No COI declaration<br>b) Funding acknowledged (academic /health research institutions) and Pfizer Belgium

For peer review only

Picardi et al. (2005)	Country: Italy<br><br>Setting: Hospital (dermatology inpatients)<br><br>Age (yrs): M = 37.5<br><br>Female: 56%	N = 141<br><br>Depressed: 8.5%	Administration: Self-report<br><br>Language: Italian	DSM-IV SCID	a) No COI declaration<br>b) Funding acknowledged (academic /health research institutions). Acknowledged Pfizer Italia SRL for providing the Italian version of the PHQ-9 and for permission to use it.
Stafford et al. (2007)	Country: Australia<br><br>Setting: Hospital (cardiology patients)<br><br>Age (yrs): M = 64.1 (SD = 10.3)<br><br>Female: 66%	N = 193<br><br>Depressed: 18%	Administration: Self-report<br><br>Language: English	DSM–IV MINI	a) No COI declaration<br>b) Funding acknowledged (academic/health research institutions)
Thombs et al. (2008)	Country: US<br><br>Setting: Hospital (outpatients with coronary heart disease)<br><br>Age (yrs): M = 67 (SD = 11)<br><br>Female: 18%	N = 1024<br><br>Depressed: 22%	Administration: Not stated<br><br>Language: English	DSM C-DIS	a) COI declaration "None disclosed"<br>b) Funding acknowledged (academic/health research institutions)

For peer review only - http://bmjopen.bmj.com/site/about/guidelines.xhtml

Thompson et al. (2010)	Country: US<br><br>Setting: Patients with Parkinson Disease<br><br>Age (yrs): 72.5 (SD = 9.6)<br><br>Female: 42%	N = 214<br><br>Depressed: 14%	Administration: Self administered<br><br>Language: English	DSM-IV SCID	a) No COI declaration<br>b) Funding acknowledged (academic/health research institutions)
Turner et al. (2012)	Country: Australia<br><br>Setting: Stroke patients<br><br>Age (yrs): 66.7 (SD = 13.1)<br><br>Female: 47.2%	N = 72<br><br>Depressed: 18%	Administration: Self administered<br><br>Language: English	DSM-IV SCID	a) COI declaration: Disclosures 'None'.<br>b) Funding acknowledged (academic/health research institutions)

For peer review only - http://bmjopen.bmj.com/site/about/guidelines.xhtml

van Steenbergen-Weijenburg (2010)	Country: Netherlands<br><br>Setting: Diabetes patients<br><br>Age (yrs): M = 61.8 (SD = 13.6)<br><br>Female: 48.7%	N = 197<br>Depressed: 18.8%	Administration: Self administered<br><br>Language: Dutch	DSM-IV<br>SCID	a) COI declaration: 'The authors declare that they have no competing interests'.<br>b) Funding acknowledged (academic/health research institutions) – 'this had no influence on the content of this article'.
Zuitthoff et al. (2010)	Country: Netherlands<br><br>Setting: Primary care<br><br>Age (yrs): M = 51 (sd = 16.7)<br><br>Female: 63%	N = 1338<br><br>Depressed: 13%	Administration: Self-report<br><br>Language: Dutch	DSM-IV<br>CIDI	a) COI declaration 'The authors declare that they have no competing interests.'<br>b) Funding acknowledged (academic/health research institutions).

For peer review only - http://bmjopen.bmj.com/site/about/guidelines.xhtml

Table 2: Descriptive characteristics of the summed items scoring method studies cut-off point 10 (Moriarty et al, 2015)					
Study	Sample characteristics	Sample size and % MDD	PHQ-9 characteristics	Diagnostic standard	Conflict of interest (COI) declaration<br>Funding<br>c) Relationship with original developers
13. Gräfe et al. (2004)	Country: Germany<br><br>Setting: psychosomatic walk-in clinics and family practices<br><br>Mean age: 41.9 (SD = 13.8)<br><br>Female: 67.8%	N = 528<br><br>Depressed: 29.2% psychosomatic patients; 6.16% medical patients	Administration: self-report<br><br>Language: German<br><br>Cut-offs: 10 to 14	DSM-IV SCID	No COI declaration<br>Acknowledged funding from Pfizer<br>Not acknowledged
16. Kroenke et al. (2001)	Country: USA<br><br>Setting: Primary care<br><br>Mean age: 46 (SD=17)<br><br>Female: 66%	N = 580<br><br>7.1% MDD	Administration: Self-report<br><br>Language: English<br><br>Cut-offs: 9 to 15	DSM-IV SCID	a) No COI declaration<br>b) Acknowledged funding from Pfizer<br>c) N/A
22. Navinés et al. (2012)	Country: Spain<br><br>Setting: General hospital (patients with chronic HCV)<br><br>Mean age: 43.4 (SD = 10.2)<br><br>Female: 28.6%	N = 500<br><br>6.4% MDD	Administration: Self-report<br><br>Language: Spanish<br><br>Cut-offs: 10	DSM–IV SCID	`a) All authors declared that they had no COI.<br>b) Role of funding source declared<br>c) Not acknowledged
29. Thekkumpurath et al. (2010)	Country: UK<br><br>Setting: Hospital (cancer patients)<br><br>Mean age: 61<br><br>Female: 63%	N = 782<br><br>6.3% MDD (of the whole sample)	Administration: Not stated<br><br>Language: English<br><br>Cut-offs: 5 to 10	DSM-IV SCID	c) COI declaration: 'Supported by Cancer Research UK'<br>d) As in a)<br>e) Not acknowledged
33. Williams et al. (2005)	Country: USA	N = 316	Administration: Unclear	DSM-IV SCID	a) No COI declaration<br>b) Funding

For peer review only - http://bmjopen.bmj.com/site/about/guidelines.xhtml

	Setting: Secondary care (Post-stroke)<br><br>Mean age: Unclear<br><br>Female: Unclear	33.5% MDD	Language: English<br><br>Cut-offs: 10		acknowledged (academic institutions)<br>c) Not acknowledged

1. Adewuya et al. (2006)	Country: Nigeria<br><br>Setting: community (students)<br><br>Mean age: 24.8 (15-40)<br><br>Female: 41.2%	N = 512<br><br>2.5% MDD	Administration: Self-report<br><br>Language: English<br><br>Cut-offs: 8 to 12	DSM-IV MINI	a) No COI declaration<br>b) No funding declaration
2. Arroll et al. (2010)	Country: New Zealand<br><br>Setting: Primary care<br><br>Mean age: 49 (17-99)<br><br>Female: 61%	N = 2642<br><br>6.2% MDD	Administration: Not stated<br><br>Language: English<br><br>Cut-offs: 8,10,12,15	DSM-IV SCID	a) No COI declaration<br>b) Funding acknowledged (academic /health research institutions)
3. Azah et al. (2005)	Country: Malaysia<br><br>Setting: Primary care<br><br>Mean age: 38.7 (18-79)<br><br>Female: 61.7%	N = 180<br><br>16.6% MDD	Administration: Self-report<br><br>Language: Malay<br><br>Cut-offs: 5 to 12	DSM-IV CIDI	b) No COI declaration<br>c) Funding acknowledged (academic /health research institutions)
4. Chagas et al. (2013)	Country: Brazil	N = 84	Administration: self-report	DSM-IV SCID	a) COI declaration “None declared”

For peer review only

	Setting: Secondary care<br><br>Mean age: Not stated<br><br>Female: 52.7%	25.5% MDD	Language: Brazilian<br><br>Cut-offs: 7 to 10		b) Funding acknowledged (academic/health research institutions)
6. de Lima Osorio et al. (2009)	Country: Brazil<br><br>Setting: Primary care<br><br>Mean age: Unclear<br><br>Female: 100%	N = 177<br><br>34% MDD	Administration: research assistants<br><br>Language: Brazilian Portuguese<br><br>Cut-offs: 10 to 15	DSM-IV SCID	a) No COI declaration<br>b) Funding acknowledged (academic institutions)
7. Elderon et al. (2011)	Country: USA<br><br>Setting: Secondary care<br><br>Mean age: Unclear<br><br>Female: 18%	N = 1022<br><br>18.3% MDD	Administration: self-report<br><br>Language: English<br><br>Cut-offs: 10	C-DIS	a) COI declaration – 'No disclosures'<br>b) Funding acknowledged (academic institutions and industry – AHA Pharmaceuticals Roundtable) – 'The funding organisations had no role in the design or conduct of the study, collection, management, analysis or interpretation of data; or preparation, review or approval of the manuscript.'
8. Fann et al. (2005)	Country: US<br><br>Setting: Trauma hospital (inpatients with traumatic	N = 135<br><br>16.3% MDD	Administration: Telephone-administered<br><br>Language: English	DSM-IV SCID	b) No COI declaration<br>c) Funding acknowledged (academic

For peer review only - http://bmjopen.bmj.com/site/about/guidelines.xhtml

	brain injury)<br><br>Mean age: 42 (SD=17.9)<br><br>Female: 29.1%		Cut-offs: 10		institutions)
9. Fine et al. (2013)	Country: USA<br><br>Setting: Primary care (Ohio Army National Guard)<br><br>Mean age: 31 (17-60)<br><br>Female: 12%	N = 498<br><br>21.5% MDD	Administration: Telephone-administered<br><br>Language: English<br><br>Cut-offs: 10,15	DSM-IV SCID-I	a) COI – last author disclosed financial and consulting interests (Pfizer not one of them). All other authors declared that they have no COI.<br>b) Funding acknowledged – DoD Medical Research. ‘’The sponsor had no role in study design, data collection, analysis, interpretation of results, report writing or manuscript submission.
10. Gelaye et al. (2013)	Country: Ethiopia<br><br>Setting: General hospital<br><br>Mean age: 34.9 (SD=11.6)<br><br>Female: 63.1 %	N = 363<br><br>12.6% MDD	Administration: Researcher-administered<br><br>Language: Amharic<br><br>Cut-offs: 9 to 11	DSM-IV SCAN	c) No COI declaration<br>d) Funding acknowledged (academic /health research institutions)
11. Gilbody et al.	Country: UK	N = 96	Administration: Not	DSM-IV	a) COI declaration –

For peer review only - http://bmjopen.bmj.com/site/about/guidelines.xhtml

(2007)	Setting: Primary care<br><br>Mean age: 42.5 (SD 13.6)<br><br>Female: 77%	37.5 MDD	stated<br><br>Language: English<br><br>Cut-offs: 9 to 13	SCID	last author involved in the development of one of the instruments (CORE-OM), 'but does not gain financially from its use.<br>b) Funding acknowledged (academic /health research institutions)
12. Gjerdingen et al. (2009)	Country: USA<br><br>Setting: Community<br><br>Mean age: 29.3<br><br>Female: 100%	N = 438<br><br>4.6% MDD	Administration: Telephone or self-report<br><br>Language: English<br><br>Cut-offs: 10	DSM-IV SCID	c) No COI declaration<br>d) Funding acknowledged (academic /health research institutions)
14. Hyphantis et al. (2011)	Country: Greece<br><br>Setting: Hospital – rheumatology patients<br><br>Mean age: 54.2 (SD = 13.5)<br><br>Female: 74%	N = 213<br><br>32.4% MDD	Administration: Researcher administered<br><br>Language: Greek<br><br>Cut-offs: 4 to 16	DSM-IV MINI	c) No COI declaration<br>d) No funding acknowledgement
15. Khamseh et al. (2011)	Country: Iran<br><br>Setting: Outpatient diabetic clinic<br><br>Mean age: 56.1 (SD=9.6)	N = 185<br><br>43.2% MDD	Administration: Self-report<br><br>Language: Persian<br><br>Cut-offs: 10,13	DSM-IV SCID	c) COI declaration: The authors declared no competing interests<br>d) Funding acknowledged (academic /health

For peer review only - http://bmjopen.bmj.com/site/about/guidelines.xhtml

	Female: 51.8%				research institutions)
19. Liu et al. (2011)	Country: Taiwan<br><br>Setting: Primary care<br><br>Mean age: Not specified<br><br>Female: 60.9%	N = 1532<br><br>3.3% MDD	Administration: Self-report<br><br>Language: Chinese version<br><br>Cut-offs: 9 to 11	SCAN	a) a) No COI declaration<br>b) Funding acknowledged (academic /health research institutions)
20. Lotrakul et al. (2008)	Country: Thailand<br><br>Setting: Primary care<br><br>Mean age: 45.0 (SD = 14.30)<br><br>Female: 73.7%	N = 279<br><br>6.8% MDD	Administration: Self report<br><br>Language: Thai<br><br>Cut-offs: 7 to 15	DSM-IV MINI	c) No COI declaration<br>d) Funding acknowledged (academic /health research institutions)
23. Patel et al. (2008)	Country: India<br><br>Setting: Primary care<br><br>Mean age: 37.5 (18-83)<br><br>Female: 56.4%	N = 299<br><br>4.3% MDD	Administration: Face-to-face interview<br><br>Language: Not specified<br><br>Cut-offs: 7 to 15	CIS-R	a) COI declaration – No Declaration of Interest<br>b) Funding acknowledged (academic /health research institutions)
24. Phelan et al. (2010)	Country: USA<br><br>Setting: Primary care (elderly)<br><br>Mean age: 78 (SD=7)<br><br>Female: 62%	N = 71<br><br>12% MDD	Administration: Research assistant<br><br>Language: English<br><br>Cut-offs: 8 to 12	DSM-IV SCID	a) COI declaration – No competing interests<br>b) Funding acknowledged (academic /health research institutions). 'The funder had no role in the study design, methods,

For peer review only - http://bmjopen.bmj.com/site/about/guidelines.xhtml

					data collection, analysis or interpretation of data, nor any role in the preparation of the manuscript or decision to submit the manuscript for publication.
25. Rooney et al. (2013)	Country: UK<br><br>Setting: Secondary care (glioma)<br><br>Mean age: 54.2 (SD=12.3)<br><br>Female: 42.6%	N = 129<br><br>13.5% MDD	Administration: Self-report<br><br>Language: English<br><br>Cut-offs: 8 to 11	DSM-IV SCID	a) COI declaration "The authors declare that they have no COI"<br>b) Funding acknowledged (academic/health research institutions)
26. Sherina et al. (2012)	Country: Malaysia<br><br>Setting: Primary care<br><br>Mean age: 30.9 (18-81)<br><br>Female: 100%	N= 146<br><br>21.2% MDD	Administration: Self-report<br><br>Language: Malay<br><br>Cut-offs: 10	CIDI	a) COI declaration "The authors declare that they have no competing interests"<br>b) Funding acknowledged (academic/health research institutions)
27. Sidebottom et al. (2012)	Country: USA<br><br>Setting: Community (prenatal)<br><br>Mean age: 23 (SD=5.5)<br><br>Female: 100%	N = 745<br><br>3.6% MDD	Administration: Interview<br><br>Language: English<br><br>Cut-offs: 10	DSM-IV SCID	b) COI declaration "The authors declare that they have no financial COI"<br>b) Funding acknowledged (academic/health research institutions)
28. Stafford et al. (2007)	Country: Australia<br><br>Setting: Secondary care (cardiac procedures)	N = 193<br><br>18.1% MDD	Administration: Self-report<br><br>Language: English	DSM-IV MINI	b) No COI declaration<br>c) Funding acknowledged (academic/health

For peer review only - http://bmjopen.bmj.com/site/about/guidelines.xhtml

	Mean age: 64.14 (38-91)<br><br>Female: 19.2%		Cut-offs: 10		research institutions)
30. Thombs et al. (2008)	Country: US<br><br>Setting: Hospital (outpatients with coronary heart disease)<br><br>Mean age: 67 (SD = 11)<br><br>Female: 18%	N = 1024<br><br>22% MDD	Administration: Not stated<br><br>Language: English<br><br>Cut-offs: 7 to 10	DSM C-DIS	b) COI declaration "None disclosed"<br>b) Funding acknowledged (academic/health research institutions)
32. Watnick et al. (2005)	Country: USA<br><br>Setting: Secondary care (dialysis)<br><br>Mean age: 63 (SD=15)<br><br>Female: 32.3%	N = 62<br><br>19% MDD	Administration: Self-report<br><br>Language: English<br><br>Cut-offs: 10	DSM-IV SCID	b) No COI declaration<br>c) Funding acknowledged (academic/health research institutions)
34. Wittkampf et al. (2009)	Country: Netherlands<br><br>Setting: Primary care<br><br>Mean age: 49.8<br><br>Female: 66.7%	N = 664<br><br>12.3% MDD	Administration: Self-report<br><br>Language: Not specified<br><br>Cut-offs: 10 and 15	DSM-IV SCIDI	No COI declaration<br>b) Funding acknowledged (academic/health research institutions)
35. Zhang et al. (2013)	Country: Hong Kong<br><br>Setting: Secondary care (diabetic outpatients)<br><br>Mean age: 55.1 (SD=9.5)	N = 99<br><br>23.2% MDD	Administration: Self-report<br><br>Language: Chinese version<br><br>Cut-offs: 15	DSM-IV MINI	COI declaration – last author acknowledged financial COI. The other authors declare that they have no competing interests.<br>) Funding acknowledged

For peer review only - http://bmjopen.bmj.com/site/about/guidelines.xhtml

	Female: 40.8%				(academic/health research institutions)
36. Zuithoff et al. (2010)	Country: Netherlands<br><br>Setting: Primary care<br><br>Age (yrs): M = 51 (sd = 16.7)<br><br>Female: 63%	N = 1338<br><br>Depressed: 13%	Administration: Self-report<br><br>Language: Dutch	DSM-IV CIDI	b) COI declaration "The authors declare that they have no competing interests.<br>b) Funding acknowledged (academic/health research institutions)

Table 3: Quality assessment of included studies in the algorithm meta-analysis (Manea et al., 2014)

**Study**	**Patient selection:** Consecutive or random sample	**Patient selection:** Avoid case-control / avoid artificially inflated base rate	**Patient selection:** Avoided inappropriate exclusions	**Patient selection:** Overall risk of bias	**Index test:** PHQ-9 interpreted blind to reference test	**Index test:** If translated, appropriate translation	**Index test:** If translated, psychometric properties reported	**Index test:** Overall risk of bias
**Allegiant studies**								
Diez-Quevedo et al. (2001)	×	✓	×	High	?	✓	✓	Unclear
Gräfe et al. (2004)	✓	✓	✓	Low	?	✓	✓	Unclear

For peer review only

Lowe et al. (2004)	×	✓	✓	High	✓	✓	✓	Low
Muramatsu et al. (2007)	?	✓	?	Unclear	✓	✓	?	Unclear
Navines et al. (2012)	✓	✓	✓	Low	✓	✓	?	Unclear
Spitzer et al. (1999)	×	✓	✓	High	✓	n/a	n/a	Low
Thekkumpurath et al. (2010)	×	×	✓	High	✓	n/a	n/a	Low
**Non-allegiant studies**								
Arroll et al. (2010)	✓	✓	✓	Low	✓	n/a	n/a	Low
Ayalon et al. (2010)	?	✓	✓	Unclear	?	✓	?	Unclear
Eack et al. (2006)	?	✓	?	Unclear	?	n/a	n/a	Unclear
Fann et al. (2005)	✓	×	×	High	✓	n/a	n/a	Low
Gelaye et al. (2013)	?	×	?	High	✓	✓	?	Unclear
Gjerdingen et	✓	✓	✓	Low	?	n/a	n/a	Unclear

For peer review only - http://bmjopen.bmj.com/site/about/guidelines.xhtml

al. (2009)								
Henkel et al. (2004)	✓	✓	✓	Low	?	n/a	n/a	Unclear
Hyphantis et al. (2011)	✓	✓	✗	High	✓	?	?	Unclear
Inagaki et al. (2013)	✓	✗	✓	High	✓	?	?	Unclear
Khamseh et al. (2011)	✓	✓	?	Unclear	✓	✓	?	Unclear
Lamers et al (2008)	✓	✗	✗	High	✓	?	?	Unclear
Lotrakul et al. (2008)	✗	✓	?	High	✓	✓	?	Unclear
Persoons et al. (2003)	✓	✓	✓	Low	✓	✓	n/a	Low
Picardi et al. (2005)	✓	✓	✓	Low	✓	?	?	Unclear
Stafford et al. (2007)	✓	✓	✓	Low	✓	n/a	n/a	Low
Thombs et al. (2008)	✗	✓	?	Unclear	?	n/a	n/a	Unclear
Thomspon et	?	✓	✓	Unclear	?	n/a	n/a	Unclear

For peer review only

al. (2011)								
Turner et al. (2012)	✓	✓	✓	Low	✓	n/a	n/a	Low
Van Steenbergen-Wijenburg (2010)	?	✓	✓	Unclear	?	?	?	Unclear
Zuithoff et al. (2010)	✓	✓	✓	Low	✓	✓	?	Unclear

✓ = criterion met; × = criterion not met; ? = insufficient information to code whether criterion met; n/a = not applicable

Table 3: Quality assessment of included studies in the algorithm meta-analysis (Manea et al., 2014) (continued)

Study	**Reference test:** Reference test correctly classifies target condition	**Reference test:** Reference test interpreted blind to PHQ-9	**Reference test:** If translated, appropriate translation	**Reference test:** If translated, psychometric properties reported	**Reference test:** Overall risk of bias	**Flow / timing:** Interval of two weeks or less	**Flow / timing:** All participants receive same reference test	**Flow / timing:** All participants included in analysis?	**Flow / timing:** Overall risk of bias
**Allegiant studies**									
Diez-Quevedo et al. (2001)	✓	✓	✓	?	Unclear	✓	✓	✓	Low

For peer review only

Gräfe et al. (2004)	✓	?	n/a	n/a	Unclear	✓	✓	✓	Low
Lowe et al. (2004)	✓	✓	n/a	n/a	Low	✓	✓	✓	Low
Muramatsu et al. (2007)	✓	✓	✓	✓	Low	✓	✓	?	Unclear
Navines et al. (2012)	✓	✓	?	?	Unclear	✓	✓	✓	Low
Spitzer et al. (1999)	✓	✓	n/a	n/a	Low	✓	✓	×	High
Thekkumpurath et al. (2010)	✓	✓	n/a	n/a	Low	?	✓	×	High
**Non-allegiant studies**									
Arroll et al. (2010)	✓	✓	n/a	n/a	Low	✓	✓	✓	Low
Ayalon et al. (2010)	✓	?	✓	?	Unclear	?	✓	✓	Unclear
Eack et al. (2006)	✓	?	n/a	n/a	Unclear	?	✓	?	Unclear
Fann et al. (2005)	✓	?	n/a	n/a	Unclear	✓	✓	×	High
Gelaye et al.	✓	✓	✓	✓	Low	✓	✓	×	High

For peer review only - http://bmjopen.bmj.com/site/about/guidelines.xhtml

For peer review only

(2013)									
Gjerdingen et al. (2009)	✓	?	n/a	n/a	Unclear	✓	✓	×	High
Henkel et al. (2004)	✓	?	n/a	n/a	Unclear	✓	✓	×	High
Hyphantis et al. (2011)	✓	✓	?	?	Unclear	✓	✓	×	High
Inagaki et el. (2013)	✓	✓	✓	?	Unclear	✓	✓	×	High
Khamseh et al (2011)	✓	✓	✓	?	Unclear	✓	✓	?	Unclear
Lamers et al. (2008)	✓	✓	?	?	Unclear	?	✓	×	High
Lotrakul et al. (2008)	✓	✓	✓	✓	Low	?	✓	×	High
Persoons et al. (2003)	✓	✓	?	?	Unclear	✓	✓	✓	Low
Picardi et al. (2005)	✓	✓	✓	?	Unclear	✓	✓	×	High
Stafford et al. (2007)	✓	✓	n/a	n/a	Low	✓	✓	×	High
Thombs et al.	?	✓	n/a	n/a	Unclear	✓	✓	✓	Low

For peer review only - http://bmjopen.bmj.com/site/about/guidelines.xhtml

Table 4: Quality assessment of included studies in the summed item scoring method cut-off point 10 meta-analysis (Moriarty et al., 2015)

(2008)									
Thompson et al. (2011)	✓	?	n/a	n/a	Unclear	✓	✓	×	High
Turner et al. (2012)	✓	?	n/a	n/a	Unclear	?	✓	×	High
Van Steenbergen-Wijenburg (2010)	✓	×	?	?	High	✓	✓	×	High
Zuithoff et al. (2010)	✓	✓	?	?	Unclear	?	✓	✓	Unclear

✓ = criterion met; × = criterion not met; ? = insufficient information to code whether criterion met; n/a = not applicable

For peer review only

Study	Patient selection: Consecutive or random sample	Patient selection: Avoid case-control / avoid artificially inflated base rate	Patient selection: Avoided inappropriate exclusions	Patient selection: Overall risk of bias	Index test: PHQ-9 interpreted blind to reference test	Index test: Was a threshold pre-specified?	Index test: If translated, appropriate translation	Index test: If translated, psychometric properties reported	Index test: Overall risk of bias
**Allegiant studies**									
13. Gräfe et al. (2004)	✓	✓	✓	Low	?	✓	✓	✓	Unclear
16. Kroenke et al. (2011)	✓	✓	✓	Low	✓	✓	n/a	n/a	Low
22. Navinés et al. (2012)	✓	✓	✓	Low	✓	✓	✓	?	Unclear
29. Thekkumpurath et al. (2010)	✗	✗	✓	High	✓	✓	n/a	n/a	Low
33. Williams et al. (2005)	✓	✓	✓	Low	?	✓	n/a	n/a	Unclear
**Non-allegiant studies**									
1. Adewuya et	✓	✓	✗	Unclear	✓	✓	n/a	n/a	Low

For peer review only - http://bmjopen.bmj.com/site/about/guidelines.xhtml

al. (2006)									
2. Arroll et al. (2010)	✓	✓	✓	Low	✓	✓	n/a	n/a	Low
3. Azah et al. (2005)	✓	✗	?	High	✓	✓	✓	✓	Low
4. Chagas et al. (2013)	✓	✓	✓	Low	✓	✓	✓	✓	Low
6. de Lima Osorio et al. (2009)	✓	✗	✓	High	?	✗	n/a	n/a	High
7. Elderon et al. (2011)	✓	✓	✓	Low	✓	✓	n/a	n/a	Low
8. Fann et al. (2005)	✓	✗	✗	High	✓	✓	n/a	n/a	Low
9. Fine et al. (2013)	✓	✓	✓	Low	?	✓	n/a	n/a	Unclear
10. Gelaye et al. (2013)	?	✗	?	High	✓	✗	✓	?	High
11. Gilbody et al.	?	✓	?	Unclear	✓	✓	n/a	n/a	Low

For peer review only - http://bmjopen.bmj.com/site/about/guidelines.xhtml

(2007)									
12. Gjerdingen et al. (2009)	✓	✓	✓	Low	?	✓	n/a	n/a	Unclear
14. Hyphantis et al. (2011)	✓	✗	✓	High	✓	✓	?	?	Unclear
15. Khamseh et al. (2011)	✓	✓	?	Unclear	✓	✓	✓	?	Unclear
19. Liu et al. (2011)	✓	✓	?	Unclear	✓	✗	✓	?	High
20. Lotrakul et al. (2008)	✗	✓	?	Unclear	✓	✓	✓	?	Unclear
23. Patel et al. (2008)	✓	✓	✓	Low	✓	✓	?	?	Unclear
24. Phelan et al. (2010)	✗	✓	✓	High	✓	✗	n/a	n/a	High
25. Rooney et al. (2013)	✓	✓	✓	Low	?	✗	n/a	n/a	High
26. Sherina et al.	✓	✓	✗	High	✓	✓	✓	✓	Low

For peer review only - http://bmjopen.bmj.com/site/about/guidelines.xhtml

(2012)									
27. Sidebottom et al. (2012)	✓	✓	✓	Low	✓	✓	n/a	n/a	Low
28. Stafford et al. (2007)	✓	✓	✓	Low	✓	✓	n/a	n/a	Low
30. Thombs et al. (2008)	✗	✓	?	High	✓	?	n/a	n/a	Unclear
32. Watnick et al. (2005)	?	✗	✓	High	✓	✓	n/a	n/a	Low
34. Wittkampf et al. (2009)	✓	✓	✓	Low	✓	?	n/a	n/a	Unclear
35. Zhang et al. (2013)	✓	✓	?	Unclear	?	✓	?	?	Unclear
36. Zuithoff et al. (2010)	✓	✓	✓	Low	✓	✓	✓	?	Unclear

Table 4: Quality assessment of included studies in the summed item scoring method cut-off point 10 meta-analysis (Moriarty et al., 2015) (continued)

Study	Reference test: Reference test	Reference test: Reference test	Reference test: If translated,	Reference test: If translated, psychometric	Reference test: Overall risk of	Flow / timing: Interval of two	Flow / timing: All participants	Flow / timing: All participants	Flow / timing: Overall risk of

For peer review only

For peer review only - http://bmjopen.bmj.com/site/about/guidelines.xhtml

	correctly classifies target condition	interpreted blind to PHQ-9	appropriate translation	properties reported	bias	weeks or less	receive same reference test	included in analysis?	bias
**Allegiant studies**									
13. Gräfe et al. (2004)	✓	?	n/a	n/a	Unclear	✓	✓	✓	Low
16. Kroenke et al. (2011)	✓	✓	n/a	n/a	Low	✓	✓	✓	Low
22. Navinés et al. (2012)	✓	✓	?	?	Unclear	✓	✓	✓	Low
29. Thekkumpurath et al. (2010)	✓	✓	n/a	n/a	Low	?	✓	✓	Unclear
33. Williams et al. (2005)	✓	?	n/a	n/a	Unclear	?	✓	✓	Unclear
Non-allegiant studies									
1. Adewuya et al. (2006)	✓	✓	n/a	n/a	Low	✓	✓	✓	Low
2. Arroll et al.	✓	✓	n/a	n/a	Low	?	✓	✓	Unclear

For peer review only - http://bmjopen.bmj.com/site/about/guidelines.xhtml

(2010)									
3. Azah et al. (2005)	✓	✓	✓	✓	Low	✓	✓	✗	High
4. Chagas et al. (2013)	✓	✓	?	?	Unclear	✓	✓	✗	High
6. de Lima Osorio et al. (2009)	✓	?	n/a	n/a	Unclear	?	✓	✓	Unclear
7. Elderon et al. (2011)	✓	✓	n/a	n/a	Low	✓	✓	✓	Low
8. Fann et al. (2005)	✓	?	n/a	n/a	Unclear	✓	✓	✗	High
9. Fine et al. (2013)	✓	?	n/a	n/a	Unclear	?	✓	✓	Unclear
10. Gelaye et al. (2013)	✓	✓	✓	✓	Low	✓	✓	✗	High
11. Gilbody et al. (2007)	✓	✓	n/a	n/a	Low	?	✓	✓	Unclear
12. Gjerdingen	✓	?	n/a	n/a	Unclear	✓	✓	✗	High

For peer review only

et al. (2009)									
14. Hyphantis et al. (2011)	✓	✓	?	?	Unclear	✓	✓	✗	High
15. Khamseh et al. (2011)	✓	✓	✓	?	Unclear	✓	✓	?	Unclear
19. Liu et al. (2011)	✓	✓	✓	✓	Low	✓	✓	?	Unclear
20. Lotrakul et al. (2008)	✓	✓	✓	✓	Low	?	✓	✗	High
23. Patel et al. (2008)	✓	✓	✓	?	Unclear	?	✓	✗	High
24. Phelan et al. (2010)	✓	✓	n/a	n/a	Low	✓	✓	✓	Low
25. Rooney et al. (2013)	✓	?	n/a	n/a	Unclear	?	✓	✗	High
26. Sherina et al. (2012)	✓	✓	✓	✓	Low	✓	✓	✓	Low
27. Sidebottom	✓	✓	n/a	n/a	Low	✓	✓	✗	High

For peer review only - http://bmjopen.bmj.com/site/about/guidelines.xhtml

et al. (2012)									
28. Stafford et al. (2007)	✓	✓	n/a	n/a	Low	✓	✓	✗	High
30. Thombs et al. (2008)	?	✓	n/a	n/a	Unclear	✓	✓	✓	Low
32. Watnick et al. (2005)	✓	✓	n/a	n/a	Low	✓	✓	✓	Low
34. Wittkampf et al. (2009)	✓	✓	n/a	n/a	Low	?	✓	✗	High
35. Zhang et al. (2013)	✓	?	✓	✓	Unclear	✗	✓	✗	High
36. Zuithoff et al. (2010)	✓	✓	?	?	Unclear	?	✓	✓	Unclear

For peer review only

Table 5. Pooled estimates of diagnostic properties of the PHQ-9 at cut-off point 10 and using algorithm scoring method in the non-independent vs independent studies groups

Settings	No of studies	No of patients	Sensitivity (95% CI)	Specificity (95% CI)	Pooled positive likelihood ratio (95% CI)	Pooled negative likelihood ratio (95% CI)	Diagnostic odds ratio (95% CI)	Heterogeneity: I^2

For peer review only - http://bmjopen.bmj.com/site/about/guidelines.xhtml

**Manea et al, 2014 SR – RA group**	7	4,065	0.77 (0.70 – 0.84)	0.94 (0.90 – 0.97)	14.97 (8.39 – 26.71)	0.23 (0.17 - 0.31)	64.40 (34.15 – 121.43)	78.9%
**Manea et al, 2014 SR Independent studies**	21	9,900	0.48 (0.41 – 0.91)	0.94 (0.91 – 0.95)	8.26 (6.15 – 11.09)	0.54 (0.48 – 0.62)	15.05 (11.03 – 20.52)	68.1%
**Moriarty et al., 2015 SR – RA group**	5	6,188	0.87 (0.77 – 0.93)	0.87 (0.76 – 0.94)	7.24 (3.74 – 14.03)	0.14 (0.08 - 0.25)	49.31 (25.74 – 94.48)	55.1%
**Moriarty et al., 2015 SR Independent studies**	26	13,164	0.76 (0.67 – 0.83)	0.88 (0.85 – 0.91)	6.72 (5.06 – 8.92)	0.26 (0.19 - 0.37)	24.96 (14.81 – 42.08)	81.5%

Figure legends

Figure 1. PHQ-9 algorithm scoring method summary ROC plot for the diagnosis of major depressive disorder in allegiant studies and non-allegiant studies. Pooled sensitivity and specificity estimates using a bi-variate meta-analysis (*HSROC* hierarchical receiver-operating characteristic).

For peer review only - http://bmjopen.bmj.com/site/about/guidelines.xhtml

585 Figure 2. PHQ-9 summed items scoring method at cut-off point 10 summary ROC plot for diagnosis of major depressive disorder in allegiant studies and non-allegiant studies. Pooled sensitivity and specificity estimates using a bi-variate meta-analysis (*HSROC* hierarchical receiver-operating characteristic).

For peer review only

For peer review only - http://bmjopen.bmj.com/site/about/guidelines.xhtml

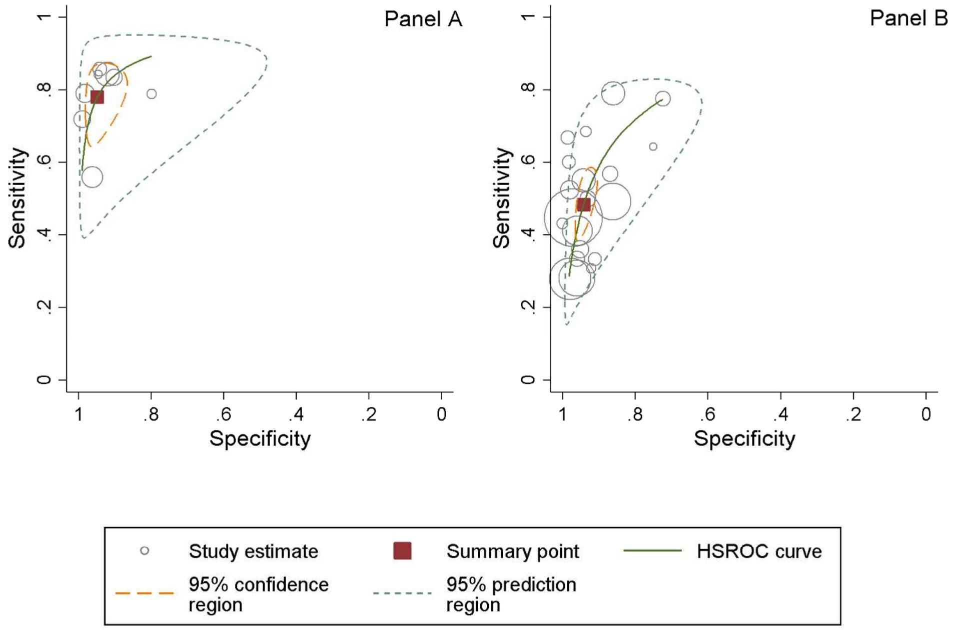

Figure 1. PHQ-9 algorithm scoring method summary ROC plot for the diagnosis of major depressive disorder in allegiant studies (Panel A) and non-allegiant studies (Panel B). Pooled sensitivity and specificity estimates using a bi-variate meta-analysis (HSROC hierarchical receiver-operating characteristic).

169x123mm (300 x 300 DPI)

For peer review only

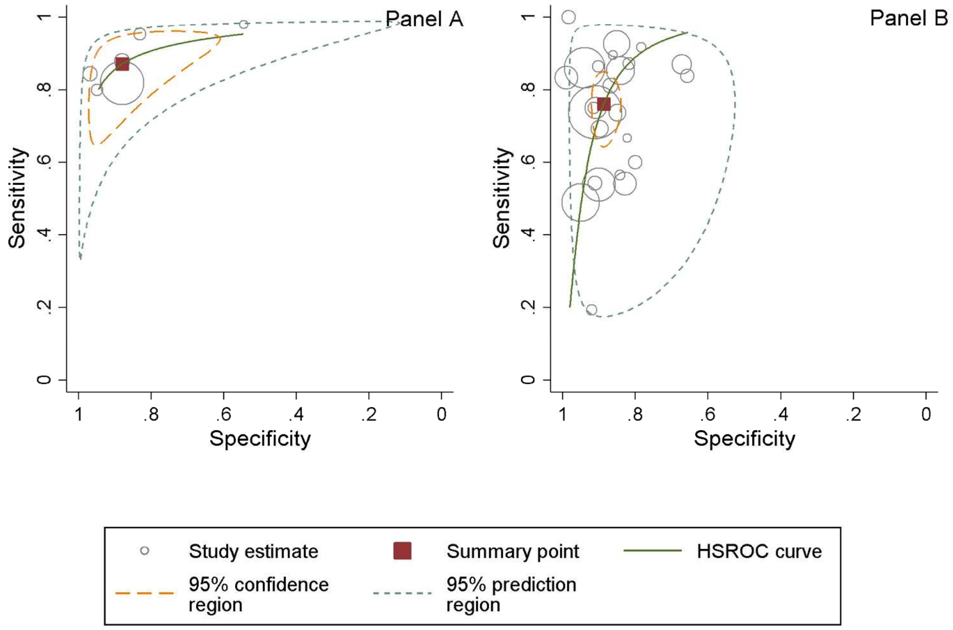

Caption : Figure 2. PHQ-9 summed items scoring method at cut-off point 10 summary ROC plot for diagnosis of major depressive disorder in allegiant studies (panel A) and non-allegiant studies (panel B). Pooled sensitivity and specificity using a bi-variate meta-analysis (HSROC hierarchical receiver-operating characteristic).

169x123mm (300 x 300 DPI)

Appendices to: Manea L, Boehnke JR, Gilbody S, Moriarty AS, McMillan D, Are there researcher allegiance effects in diagnostic validation studies of the PHQ-9? A systematic review and meta-analysis. Manuscript submitted for publication at BMJOpen.

Appendix 1: Search terms used in Embase, MEDLINE and PsycINFO

(phq adj5 “9”).ti,ab.

(phq adj5 item$).ti,ab.

(patient health questionnaire adj5 “9”).ti,ab.

(patient health questionnaire adj5 item$).ti,ab.

(prime md adj5 “9”).ti,ab.

(prime md adj5 item$).ti,ab.

For peer review only

Appendix 2

Figure 1: PRISMA flowchart - search and selection of included diagnostic accuracy studies for the systematic review of studies reporting diagnostic accuracy of the PHQ-9 at using the summed items scoring method (Manea et al, 2014)

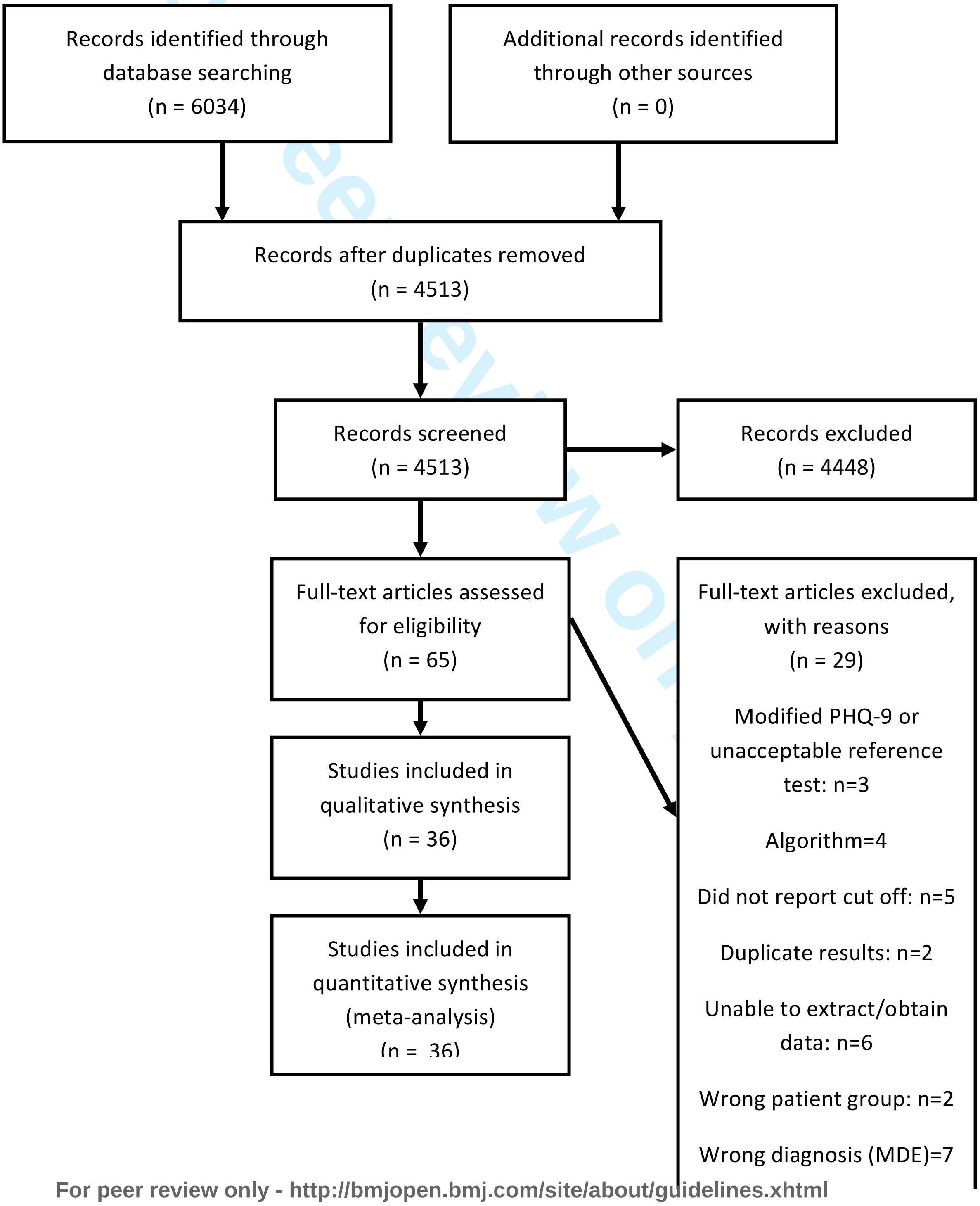

Figure 2: PRISMA flowchart - search and selection of included diagnostic accuracy studies for the systematic review of studies reporting diagnostic accuracy of the PHQ-9 at using the algorithm scoring method (Moriarty et al., 2015)

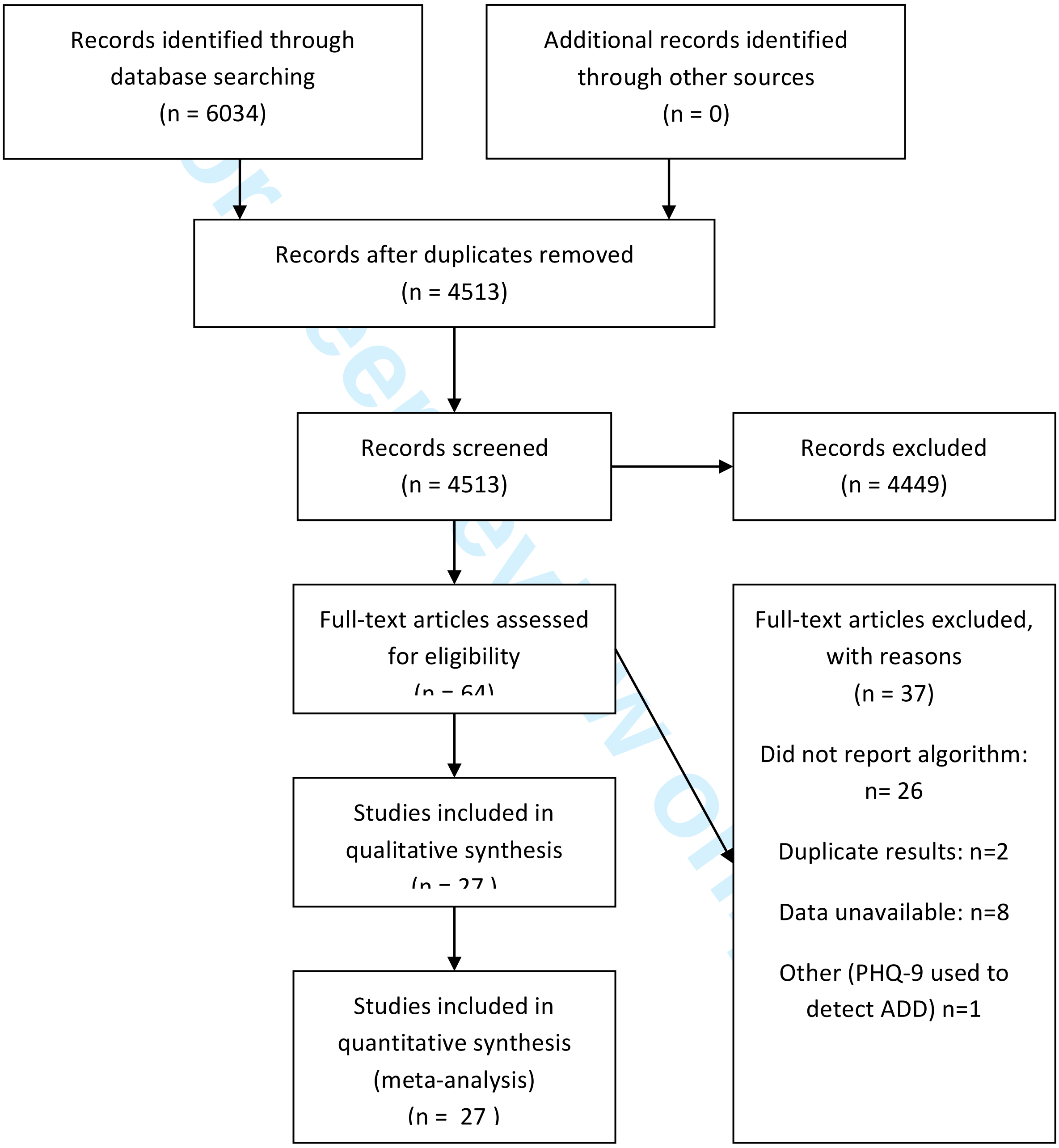

PRISMA 2009 Checklist

Section/topic	#	Checklist item	Reported on page #
**TITLE**			
Title	1	Identify the report as a systematic review, meta-analysis, or both.	1
**ABSTRACT**			
Structured summary	2	Provide a structured summary including, as applicable: background; objectives; data sources; study eligibility criteria, participants, and interventions; study appraisal and synthesis methods; results; limitations; conclusions and implications of key findings; systematic review registration number.	2
**INTRODUCTION**			
Rationale	3	Describe the rationale for the review in the context of what is already known.	4-5
Objectives	4	Provide an explicit statement of questions being addressed with reference to participants, interventions, comparisons, outcomes, and study design (PICOS).	5
**METHODS**			
Protocol and registration	5	Indicate if a review protocol exists, if and where it can be accessed (e.g., Web address), and, if available, provide registration information including registration number.	No
Eligibility criteria	6	Specify study characteristics (e.g., PICOS, length of follow-up) and report characteristics (e.g., years considered, language, publication status) used as criteria for eligibility, giving rationale.	5
Information sources	7	Describe all information sources (e.g., databases with dates of coverage, contact with study authors to identify additional studies) in the search and date last searched.	5
Search	8	Present full electronic search strategy for at least one database, including any limits used, such that it could be repeated.	Available online (see Manea et al., 2015; Moriarty et al., 2015)
Study selection	9	State the process for selecting studies (i.e., screening, eligibility, included in systematic review, and, if applicable, included in the meta-analysis).	5
Data collection process	10	Describe method of data extraction from reports (e.g., piloted forms, independently, in duplicate) and any processes for obtaining and confirming data from investigators.	6
Data items	11	List and define all variables for which data were sought (e.g., PICOS, funding sources) and any assumptions and simplifications made.	5-6
Risk of bias in individual studies	12	Describe methods used for assessing risk of bias of individual studies (including specification of whether this was done at the study or outcome level), and how this information is to be used in any data synthesis.	6

For peer review only

PRISMA 2009 Checklist

Summary measures	13	State the principal summary measures (e.g., risk ratio, difference in means).	6
Synthesis of results	14	Describe the methods of handling data and combining results of studies, if done, including measures of consistency (e.g., I^2) for each meta-analysis.	6

Section/topic	#	Checklist item	Reported on page #
Risk of bias across studies	15	Specify any assessment of risk of bias that may affect the cumulative evidence (e.g., publication bias, selective reporting within studies).	6, 21
Additional analyses	16	Describe methods of additional analyses (e.g., sensitivity or subgroup analyses, meta-regression), if done, indicating which were pre-specified.	6
**RESULTS**			
Study selection	17	Give numbers of studies screened, assessed for eligibility, and included in the review, with reasons for exclusions at each stage, ideally with a flow diagram.	Appendix
Study characteristics	18	For each study, present characteristics for which data were extracted (e.g., study size, PICOS, follow-up period) and provide the citations.	Tables 1 and 2
Risk of bias within studies	19	Present data on risk of bias of each study and, if available, any outcome level assessment (see item 12).	Tables 3 and 4
Results of individual studies	20	For all outcomes considered (benefits or harms), present, for each study: (a) simple summary data for each intervention group (b) effect estimates and confidence intervals, ideally with a forest plot.	N/A
Synthesis of results	21	Present results of each meta-analysis done, including confidence intervals and measures of consistency.	Table 5
Risk of bias across studies	22	Present results of any assessment of risk of bias across studies (see Item 15).	Tables 3 and 4
Additional analysis	23	Give results of additional analyses, if done (e.g., sensitivity or subgroup analyses, meta-regression [see Item 16]).	11 and 17
**DISCUSSION**			
Summary of evidence	24	Summarize the main findings including the strength of evidence for each main outcome; consider their relevance to key groups (e.g., healthcare providers, users, and policy makers).	17-21
Limitations	25	Discuss limitations at study and outcome level (e.g., risk of bias), and at review-level (e.g., incomplete retrieval of identified research, reporting bias).	21
Conclusions	26	Provide a general interpretation of the results in the context of other evidence, and implications for future research.	21-22
**FUNDING**			

For peer review only - http://bmjopen.bmj.com/site/about/guidelines.xhtml

PRISMA 2009 Checklist

Funding	27	Describe sources of funding for the systematic review and other support (e.g., supply of data); role of funders for the systematic review.	23

From: Moher D, Liberati A, Tetzlaff J, Altman DG, The PRISMA Group (2009). Preferred Reporting Items for Systematic Reviews and Meta-Analyses: The PRISMA Statement. PLoS Med 6(6): e1000097. doi:10.1371/journal.pmed1000097

For more information, visit: **www.prisma-statement.org**.

For peer review only

For peer review only - http://bmjopen.bmj.com/site/about/guidelines.xhtml
